# Exploring the geospatial epidemiology of breast cancer in Iran: identifying significant risk factors and spatial patterns for evidence-based prevention strategies

**DOI:** 10.1186/s12885-023-11555-1

**Published:** 2023-12-11

**Authors:** Mohsen Soleimani, Seyed Mohammad Ayyoubzadeh, Ahmad Jalilvand, Marjan Ghazisaeedi

**Affiliations:** 1 Department of Health Information Management and Medical Informatics, School of Allied Medical Sciences, Tehran University of Medical Sciences, Tehran, Iran; 2 Department of Pathology, School of Medicine, Zanjan University of Medical Sciences, Zanjan, Iran

**Keywords:** Breast cancer, Incidence rate, Geospatial epidemiology, Risk factors, Spatial patterns, Iran

## Abstract

**Background:**

Breast Cancer (BC) is a formidable global health challenge, and Iran is no exception, with BC accounting for a significant proportion of women’s malignancies. To gain deeper insights into the epidemiological characteristics of BC in Iran, this study employs advanced geospatial techniques and feature selection methods to identify significant risk factors and spatial patterns associated with BC incidence.

**Methods:**

Using rigorous statistical methods, geospatial data from Iran, including cancer-related, sociodemographic, healthcare infrastructure, environmental, and air quality data at the provincial level, were meticulously analyzed. Age-standardized incidence rates (ASR) are calculated, and different regression models are used to identify significant variables associated with BC incidence. Spatial analysis techniques, including global and local Moran's index, geographically weighted regression, and Emerging hotspot analysis, were utilized to examine geospatial patterns, identify clustering and hotspots, and assess spatiotemporal distribution of BC incidence.

**Results:**

The findings reveal that BC predominantly affects women (98.03%), with higher incidence rates among those aged 50 to 79. Isfahan (ASR = 26.1) and Yazd (ASR = 25.7) exhibit the highest rates. Significant predictors of BC incidence, such as marriage, tertiary education attainment rate, physician-to-population ratio, and PM2.5 air pollution, are identified through regression models.

**Conclusion:**

The study's results provide valuable information for the development of evidence-based prevention strategies to reduce the burden of BC in Iran. The findings underscore the importance of early detection, health education campaigns, and targeted interventions in high-risk clusters and adjacent regions. The geospatial insights generated by this study have implications for policy-makers, researchers, and public health practitioners, facilitating the formulation of effective BC prevention strategies tailored to the unique epidemiological patterns in Iran.

**Supplementary Information:**

The online version contains supplementary material available at 10.1186/s12885-023-11555-1.

## Introduction

Breast cancer (BC) constitutes a significant global burden of morbidity and mortality, particularly among women [1]. In 2020, BC represented 11.7% of new cancer diagnoses (2.35 million cases) and was the leading cause of cancer mortality in women, accounting for an estimated 6.9% of cancer deaths [2]. Approximately one in four women will be diagnosed with BC during their lifetime, with one in eight dying from the disease [3]. In Iran, the incidence of BC in women is rising precipitously, accounting for 28.1% of female malignancies. Substantial regional variations have been observed in this country, with markedly high incidence rates documented in Isfahan and Yazd provinces [4, 5]. As a critical focus for healthcare providers and researchers across gender and age strata, BC represents an urgent public health priority [6].

The etiology of BC is multifactorial, with proposed risk factors including genetic mutations, environmental exposures, lifestyle behaviors, racial and regional differences, and socioeconomic status (SES) [7–10]. Societal factors like income, education, occupation, employment, country of birth, language, religion, and migration have been associated with screening and diagnosis of BC [11]. In Iran, substantial geographic and sociocultural differences across 31 provinces significantly contribute to varying BC incidence patterns. Each province represents a distinct constellation of genetic predispositions, ethnic backgrounds, cultural practices, behaviors, and environmental conditions modulating risk of BC incidence [12]. For instance, northern humid versus central arid climates may impact risk through altered ultraviolet radiation and vitamin D deficiency. Furthermore, air pollution in industrial areas versus rural vicinities can increase carcinogen exposure prompting malignant transformations. Additionally, regional customs related to early marriage, multiparty, and sedentary lifestyles demonstrate variations affecting risk through hormonal and reproductive factors [13]. Analyzing BC incidence through a provincial lens augmented by geospatial methodologies elucidates location-specific influences on disparities, engendering knowledge to tailor prevention policies.

Concurrently, rigorous scientific inquiry is imperative to advance efficacious BC prevention, diagnosis, and treatment strategies with the potential to mitigate incidence and enhance survival [7, 14]. Epidemiological research, coupled with geographic information system (GIS) technology and feature selection methodologies, provides a pathway to profound insights into BC causation and prevention [15, 16]. GIS enables visualization of spatial patterns and identification of high-risk areas, while feature selection elucidates the most impactful risk factors to guide targeted public health interventions [15–19].

Despite being the most prevalent cancer among women in Iran, prior epidemiological research on BC utilizing population-level data has been limited, representing critical gaps in the literature. Existing research exhibits critical limitations, including reliance on pathology-based cancer registry data, restricting generalizability, and provincial rather than national geographic scope, constraining investigation of broader spatial trends. Moreover, advanced geospatial techniques remain underutilized, impeding recognition of geographic clusters, variations, and environmental associations. The relative impacts and distributions of proposed risk factors are ambiguous, and most studies exclusively emphasize females [5, 20–24]. These limitations preclude the formulation of tailored BC prevention policies and interventions for Iran.

This study aimed to address these gaps by conducting a comprehensive spatiotemporal analysis of BC incidence encompassing all provinces and genders in Iran from 2014–2018. The key goals were to map geographic and temporal patterns in BC rates, identify spatial clusters and emerging hotspots, investigate potential risk factors, pinpoint the most impactful multivariate predictors of incidence, and examine geographic variations in these predictor-BC relationships. By integrating diverse data sources and employing population-based cancer registry data with rigorous statistical techniques and cutting-edge geospatial analytics, this study sought to gain deeper insights into the intricate spatiotemporal dynamics underpinning BC epidemiology across Iran. The evidence synthesized provides a foundation to formulate tailored, geospatially targeted prevention and control strategies addressing the escalating BC burden.

## Materials and methods

### Study design

**Fig. 1:**
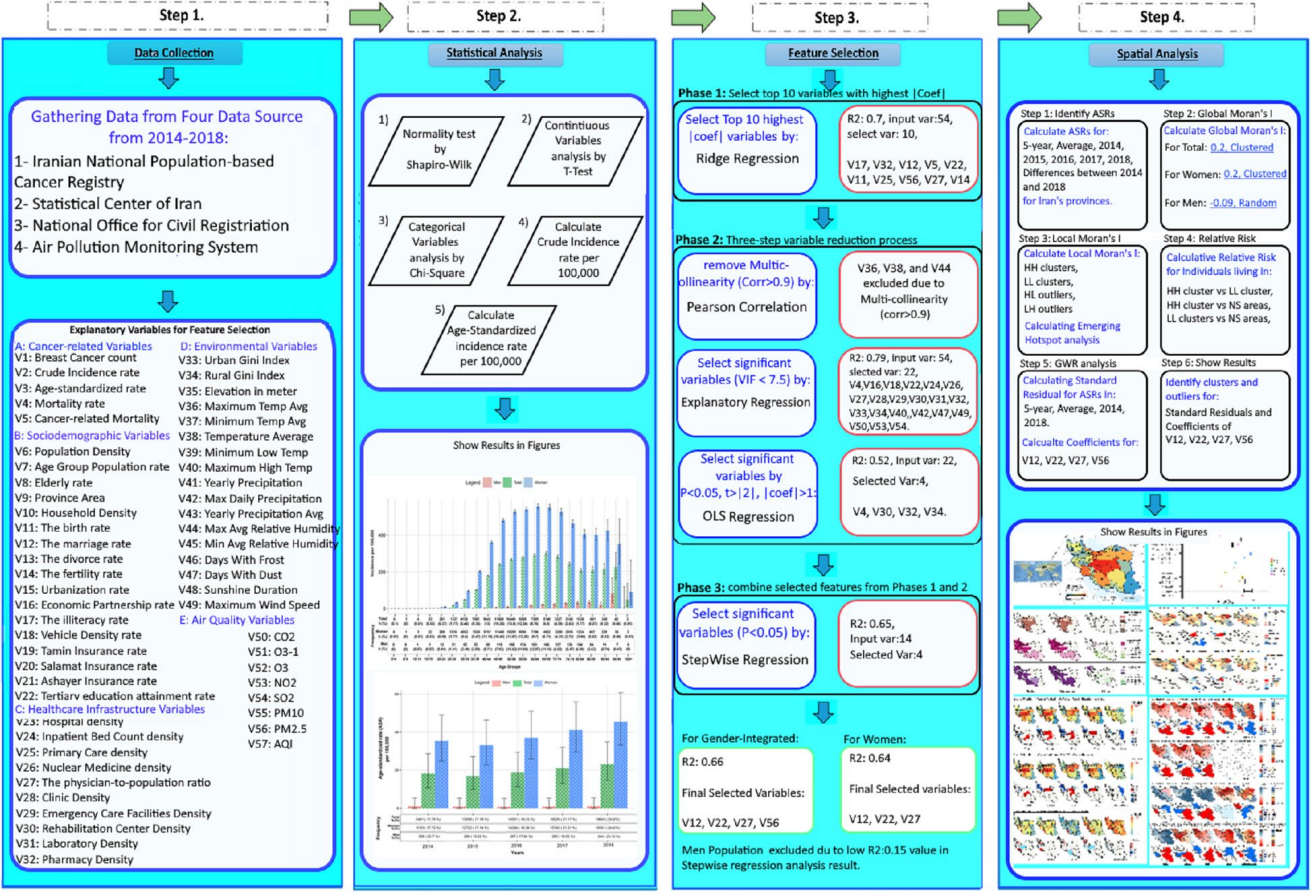
Methodology flowchart for descriptive and geospatial analysis of BC incidence in Iran

This study is a comprehensive descriptive and geospatial analysis that was conducted in Iran in 2023. The study utilized various data sources and applied statistical and geospatial methods to analyze and describe the incidence of BC in Iran. The research methodology involved four steps, including data collection, statistical analysis, feature selection, and spatial analysis, which are meticulously explained in subsequent sections of the study (Fig. 1).

### Study area

**Fig. 2:**
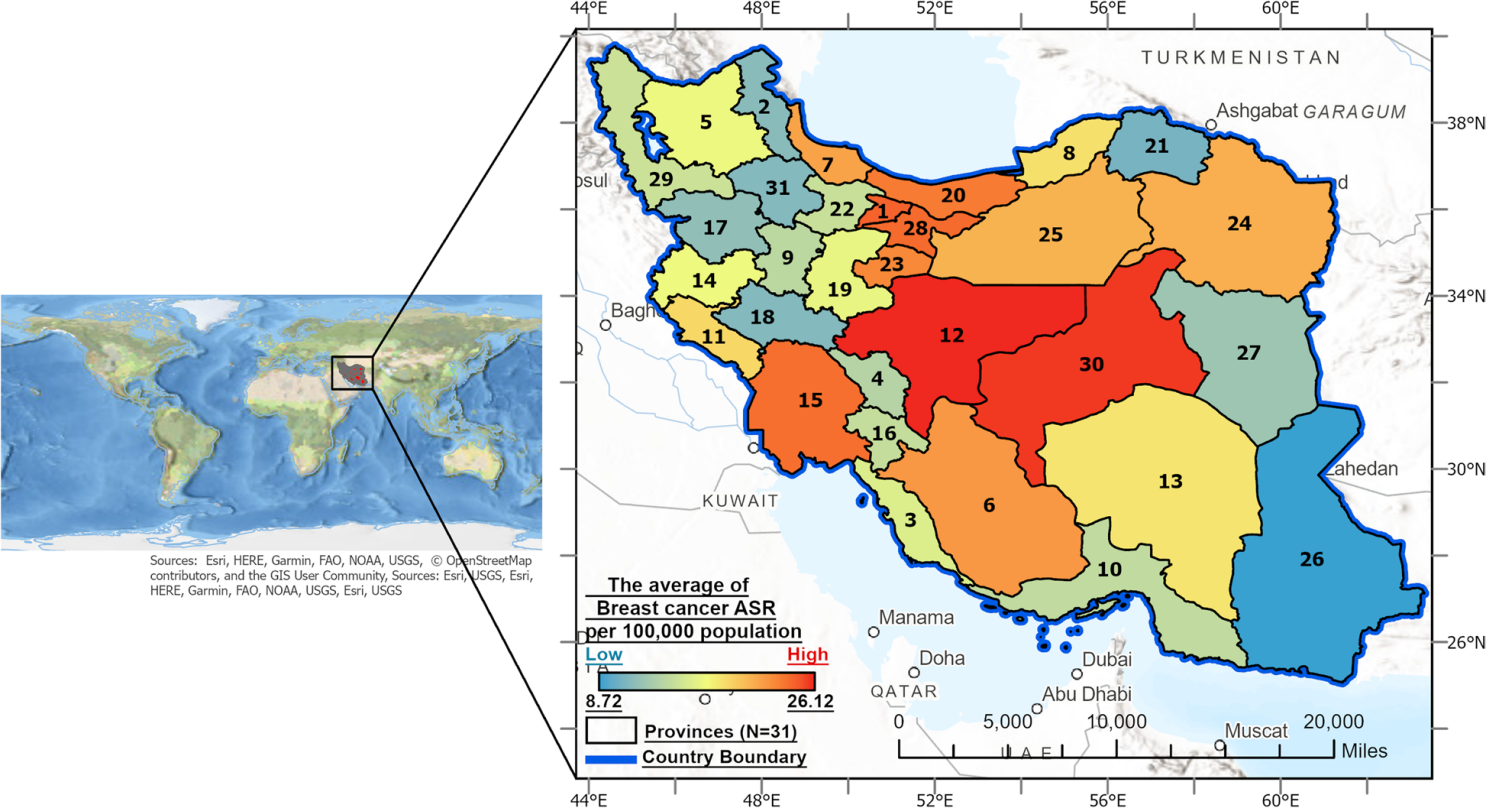
Geospatial epidemiology of BC in Iran: Average Age-Standardized Incidence Rates per 100,000 in gender-integrated population by Geographical Location, 2014–2018. Province Names (in Alphabetical Order):1- Alborz, 2- Ardabil, 3- Bushehr, 4- Chaharmahal And Bakhtiari, 5- East Azerbaijan, 6- Fars, 7- Gilan, 8- Golestan, 9- Hamedan, 10- Hormozgan, 11- Ilam, 12- Isfahan, 13- Kerman, 14- Kermanshah, 15- Khuzestan, 16- Kohgiluyeh And Boyer-Ahmad, 17- Kurdistan, 18- Lorestan,19- Markazi, 20- Mazandaran, 21- North Khorasan, 22- Qazvin, 23- Qom, 24- Razavi Khorasan, 25- Semnan, 26- Sistan And Baluchestan, 27- South Khorasan, 28- Tehran, 29- West Azerbaijan, 30- Yazd, 31- Zanjan

Iran is situated in the Middle East, bordered by the Caspian Sea to the north and the Persian Gulf to the south (Fig. 2). The country has a diverse population exceeding 85 million, with Persian as the official language. There are sizable Azeri, Kurdish, Arab, and Baloch minority communities, contributing to Iran's ethnic and religious diversity. Over two-thirds of the population lives in urban areas, concentrated primarily in the north and west, while the remaining population resides in rural regions. Iran's population is predominantly Shia Muslim. The economy depends heavily on hydrocarbon exports. Educational modernization has yielded a literacy rate surpassing 90%. While both public and private healthcare are available, rural communities suffer from poorer access. Rapid urbanization, industrialization, and climate change have severely impacted Iran's environment, with many cities experiencing severe air pollution, water shortages, droughts, deforestation, desertification, soil erosion, and biodiversity decline [12].

### Data collection

The objective of this study was to conduct a comprehensive analysis and description of diverse data sources in Iran from 2014 to 2018, utilizing geospatial methods, to investigate potential risk factors and patterns of BC incidence. Table 1 presents a detailed description of cancer-related factors, sociodemographic variables, healthcare infrastructure variables, environmental variables, and air quality variables at the provincial level, which was the main focus of this study. The study utilized the Iranian National Population-Based Cancer Registry (INPCR), administered by the Ministry of Health and Medical Education, to collect incidence data on BC. This registry collects cancer cases from multiple sources, such as pathology laboratories, hospitals, and clinics. The Statistical Center of Iran (SCI) and the National Office for Civil Registration (NOCR) provided data on population, healthcare infrastructure, and the environment. Air quality data were sourced from the Air Pollution Monitoring System of Iran (APMS), which is responsible for monitoring and regulating air pollution levels in the country. By collecting and analyzing data at the provincial level, this study offers a more comprehensive understanding of regional variations in BC incidence and their association with environmental factors. Further details on variables can be found in Supplementary file 1.

**Table 1:** Explanatory variables for feature selection in BC geospatial epidemiological analysis in Iran

Variable	Data Source
**Cancer-related Variables**	
V1: Breast Cancer count	INPCR
V2: The crude Incidence rate of BC in 100,000 population	-
V3: The age-standardized rate of BC in the 100,000 population	-
V4: The mortality rate in the 100,000 population	NOCR
V5: The cancer Mortality rate in the 100,000 population	NOCR
**Sociodemographic Variables**	
V6: Population Density	SCI
V7: Age Group Population rate in %	SCI
V8: Elderly rate in %	-
V9: Province Area in km2	SCI
V10: Household Density	SCI
V11: The birth rate in the 100,000 population	NOCR
V12: The marriage rate in the 100,000 population	NOCR
V13: The divorce rate in the 100,000 population	NOCR
V14: The fertility rate in 100,000 Women	SCI
V15: Urbanization rate in %	SCI
V16: Economic Partnership rate in %	SCI
V17: The illiteracy rate in 100,000	SCI
V18: Vehicle Density rate in 100,000 population	SCI
V19: Tamin Insurance rate in %	SCI
V20: Salamat Insurance rate in %	SCI
V21: Ashayer Insurance rate in %	SCI
V22: Tertiary education attainment rate in %	SCI
**Healthcare Infrastructure Variables**	
V23: Hospital density in 100,000 population	SCI
V24: Inpatient Bed Count density in 100,000 population	SCI
V25: Primary Care density in the 100,000 population	SCI
V26: Nuclear Medicine density in the 100,000 population	SCI
V27: The physician-to-population ratio in the 100,000 population	SCI
V28: Clinic Density in the 100,000 population rate in 100,000 population	SCI
V29: Emergency Care Facilities Density rate in the 100,000 population	SCI
V30: Rehabilitation Center Density in the 100,000 population	SCI
V31: Laboratory Density in the 100,000 population	SCI
V32: Pharmacy Density in the 100,000 population	SCI
**Environmental Variables**	
V33: Urban Gini Index	SCI
V34: Rural Gini Index	SCI
V35: Elevation in meter	SCI
V36: Maximum Temperature Average	SCI
V37: Minimum Temperature Average	SCI
V38: Temperature Average	SCI
V39: Minimum Low Temperature	SCI
V40: Maximum High Temperature	SCI
V41: Yearly Precipitation	SCI
V42: Maximum Daily Precipitation	SCI
V43: Yearly Precipitation Average	SCI
V44: Maximum Average Relative Humidity	SCI
V45: Minimum Average Relative Humidity	SCI
V46: Days With Frost	SCI
V47: Days With Dust	SCI
V48: Sunshine Duration	SCI
V49: Maximum Wind Speed	SCI
**Air Quality Variables**	
V50: Carbon Monoxide (CO2)	APMS
V51: Ozone (O3-1)	APMS
V52: Ozone (O3)	APMS
V53: Nitrogen Dioxide (No2)	APMS
V54: Sulfur Dioxide (SO2)	APMS
V55: Particulate matter of size ≤ 10 micron (PM10)	APMS
V56: Particulate matter of size ≤ 2.5 micron (PM2.5)	APMS
V57: Air Quality Index (AQI)	APMS

While this study utilizes BC data from Iran spanning 2014 to 2018, the most recent year for which data were available at the time of analysis, it must be acknowledged that more up-to-date cancer registry data may exist but have not yet been published. Quality assurance for cancer registry data is a time-intensive process. Therefore, there is frequently a lag between data collection and publication. However, missing data can be a common issue in large and complex datasets, and not all provinces may have complete data in the INPCR dataset, which can result in missing data. In the present study, an effective approach to address missing data is to substitute the respective variable's mean. It is crucial to recognize and identify any limitations that missing data may present to ensure the study's validity and rigor. The data were collected and processed in compliance with ethical principles and data protection laws.

This study utilized histopathologically confirmed primary BC cases (international classification of disease for oncology (ICD-O) code: C50.0) registered from 2014 in accredited INPCR adhering to international data quality benchmarks. Cases included complete demographic information on age, sex, geographical location, and study period for representativeness across Iran. Excluded were those diagnosed before 2014, cases reported in pathology-based cancer registry, duplicates, or cases with unknown geographical location and missing data. Data quality was ensured through accredited registries, verification of spatial coordinates, data validation checks, and consultation with registry managers. Ethical approval was obtained from the institutional review board of Tehran University of Medical Sciences (code: IR.TUMS.SPH.REC.1401.260) in alignment with research guidelines.

### Statistical analysis

The current study used a variety of statistical methodologies to determine the prevalence of BC across Iran's provinces. The Shapiro–Wilk test was used to determine the normality of the data, and T-tests were used to compare continuous variables reported as means and standard deviations. The chi-square test was used to compare categorical variables, which were given as absolute numbers and percentages. For each province and gender, the crude incidence rate per 100,000 was computed, which was then adjusted using the age-standardized incidence rate (ASR). Because it adjusts for age distribution discrepancies, ASR is an important statistic for comparing cancer incidence across populations and time. ASR identifies nonage-related differences, informs effective cancer prevention and control methods, and accounts for changes in age structures, making it critical for estimating cancer incidence accurately. The direct technique was used to alter the incidence rate, which was then normalized using the world health organization's new world standard population as the reference group [25]. For the entire country, age-specific incidence rates per 100,000 people were estimated, stratified by gender. All geographical and statistical analyses in this study made extensive use of ASR. All statistical analyses for this study were conducted using R statistical software (version 4.2.2) within the RStudio programming environment (version 2023.03) [26].

### Feature selection

Feature selection is integral in developing a robust predictive model. We employed a meticulous three-phase procedure to identify the most impactful predictors of ASR of BC within our diverse study population. In phase 1, Ridge regression was implemented via the glmnet package to quantify coefficients for all features, acknowledging potential multicollinearity. The top 10 variables were selected based on the magnitude of their coefficients, without exclusion at this initial stage. Comprehensive outputs are provided in Supplementary file 2 to promote reproducibility.

Phase 2 encompassed a three-step variable reduction process. First, the cor function in stats package was utilized to evaluate multicollinearity through Pearson correlation analysis. Variables exceeding a correlation of 0.9 were excluded, as conventions deem this indicative of significant collinearity. Next, the car package enabled explanatory regression with tenfold cross-validation to compute variance inflation factors (VIFs). Following recommended thresholds [27], variables with VIFs above 7.5 were eliminated. Finally, ordinary least squares (OLS) regression was implemented. Variables were retained based on OLS regression assumptions, with thresholds of *p* < 0.05, |t|> 2, and |coefficients|> 1. OLS regression requires uncorrelated predictors and assumes normally distributed residuals with constant variance. In contrast, explanatory regression necessitates linear relationships between predictors and outcome, alongside constant residual variance. However, these assumptions may not always hold in practice, and the techniques have limitations when selecting pivotal variables. To mitigate these constraints, we utilized a multi-step strategy harnessing the strengths of both Ridge and OLS regression in phase 3.

In Phase 3, the MASS package enabled stepwise regression to combine the selected features from Phases 1 and 2. This integration leveraged the complementary strengths of Ridge and OLS regression while upholding key assumptions. This meticulous, systematic approach provides a rigorous framework for feature selection, notwithstanding variability in order influencing results. Comprehensive outputs are provided in Supplementary file 2 to promote reproducibility. Feature selection was performed using R statistical software (version 4.2.2) within the RStudio programming environment (version 2023.03). Further details on R codes can be found in Supplementary file 3.

### Spatial analysis

A comprehensive spatiotemporal analysis of global spatial autocorrelation patterns in BC incidence rates across provinces had been undertaken using Moran's I index, an established method in spatial statistics. The primary aim of this investigation was to elucidate the presence of clustering phenomena and their dynamics within this health context. Moran's I index was utilized to scrutinize the spatial arrangement of BC incidence rates, enabling the detection of clustering tendencies between neighboring provinces. Through applying the local Moran's I statistic, statistically significant hotspots, cold spots, and spatial outliers among proximate provinces were identified, discerning nuanced geographical patterns in the data. Subsequently, Emerging Hot Spot Analysis was executed using the Getis-Ord Gi* statistic to locate regions exhibiting nascent or intensifying spatial clusters over time. This technique evaluates each time step concerning the prior period to identify areas where novel clustering trends are emerging or escalating. Furthermore, to explore the temporal dimension of clustering dynamics, hot spot trend analysis was conducted using the Mann–Kendall test. This statistical test determines statistically significant temporal trends within existing hot and cold spots, complementing the Emerging Hot Spot Analysis by investigating trajectories in the intensity of extant clusters. Notably, our analysis extended beyond solely identifying clusters by undertaking BC incidence trend mapping, a robust technique that delineates changes in incidence rates over time. This enables observing not only the clustering patterns but also their temporal evolution. Each methodology provides unique insights into the intricate spatiotemporal dynamics of BC incidence rates. To facilitate a more comprehensive understanding of these dynamics, we have employed a two-dimensional space–time cube visualization, enabling the examination of temporal trends and trajectory shifts. Spatial relationships have been conceptualized based on polygon contiguity, with row standardization applied to mitigate the influence of provinces with varying numbers of neighboring regions. This meticulous approach ensures the accuracy and reliability of our findings within the complex realm of spatiotemporal analysis in the context of BC incidence rates.

The relative risk (RR) of ASR of BC was calculated for populations residing in regions clustered into geographic areas to evaluate the risk of developing BC across different geographical regions. Geographically weighted regression (GWR) analysis was also utilized to investigate the spatiotemporal distribution of BC incidence and assess risks in diverse geographic areas. GWR accounts for spatial heterogeneity in relationships by yielding location-specific results. The residuals and regression coefficients obtained from GWR are critical for delineating regions with high or low BC incidence to guide targeted interventions [15]. The GWR standard residual was used to identify spatial trends, outliers, and significant findings. Standardized residuals are a key diagnostic for evaluating the fit and performance of a GWR model across geographic space. Mapping and analyzing patterns in the Standardized residuals is an important part of applying GWR. Standardized residuals in GWR refer to the residuals (differences between observed and predicted values) that have been standardized to have a mean of zero and standard deviation of one. In GWR, a separate regression model is estimated for each geographic location. This means that each location will have its own set of residuals. To make the residuals comparable across locations, they are standardized by subtracting the local mean and dividing by the local standard deviation. This puts the residuals from all locations on a common scale. Standardized residuals are useful for identifying outliers and influential observations in GWR. Large standardized residuals (e.g., > 2 or < -2) indicate observations that are poorly fit by the local model. Mapping the standardized residuals can help identify spatial patterns and clusters where the GWR model fits poorly. This may suggest additional spatial variables to include in the model. Global measures of model fit like R-squared are not applicable in GWR. Standardized residuals provide a local indicator of model fit at each location.

To enable assessment of geographic trends across both short-term and long-term time frames, maps were created for 5-year, average, 2014, 2018, and difference between 2014–2018 for ASRs and GWR residuals. The 5-year and average maps provide the complete picture of spatial variation over the full study period from 2014–2018, while the annual 2014 and 2018 maps give insight into shifts between the beginning and end of the timeframe. The difference maps highlight changes in incidence rates and model fit between these years. Examining multiple time points provides a more comprehensive understanding of how BC heterogeneity and model performance have evolved temporally across provinces. The expanded visualizations better capture the spatiotemporal dynamics. The geographic information system software ArcGIS Desktop version 10.8.2 and ArcGIS Pro version 3.0 were utilized to perform all spatial analyses in this study.

## Results

Out of 78,415 patients with BC, 98.05% were female and 1.95% were male. The ASR of BC in the total population, irrespective of gender, was estimated to be 98.5 per 100,000 (95% Confidence Interval (CI): 80–120) for the 5-year period from 2014 to 2018 in Iran. The ASR was higher in women (192.5; 95% CI: 166.2–221.7) than men (4.3; 95% CI: 1.2–10.3). The average ASR was 19.6 for gender-integrated population, 38.4 for women, and 0.82 per 100,000 for men. Isfahan, Yazd, and Alborz provinces had the highest average ASRs (26.1, 25.7, and 24.6, respectively), while Sistan and Baluchistan had the lowest at 8.7 per 100,000 (Fig. 2). Isfahan and Yazd also had the highest average ASRs in females (52.08 and 51.9 per 100,000), while Bushehr had the highest average ASR in male at 1.5 per 100,000. The ASR of BC per 100,000 population in each province is detailed in Supplementary file 4.

**Fig. 3:**
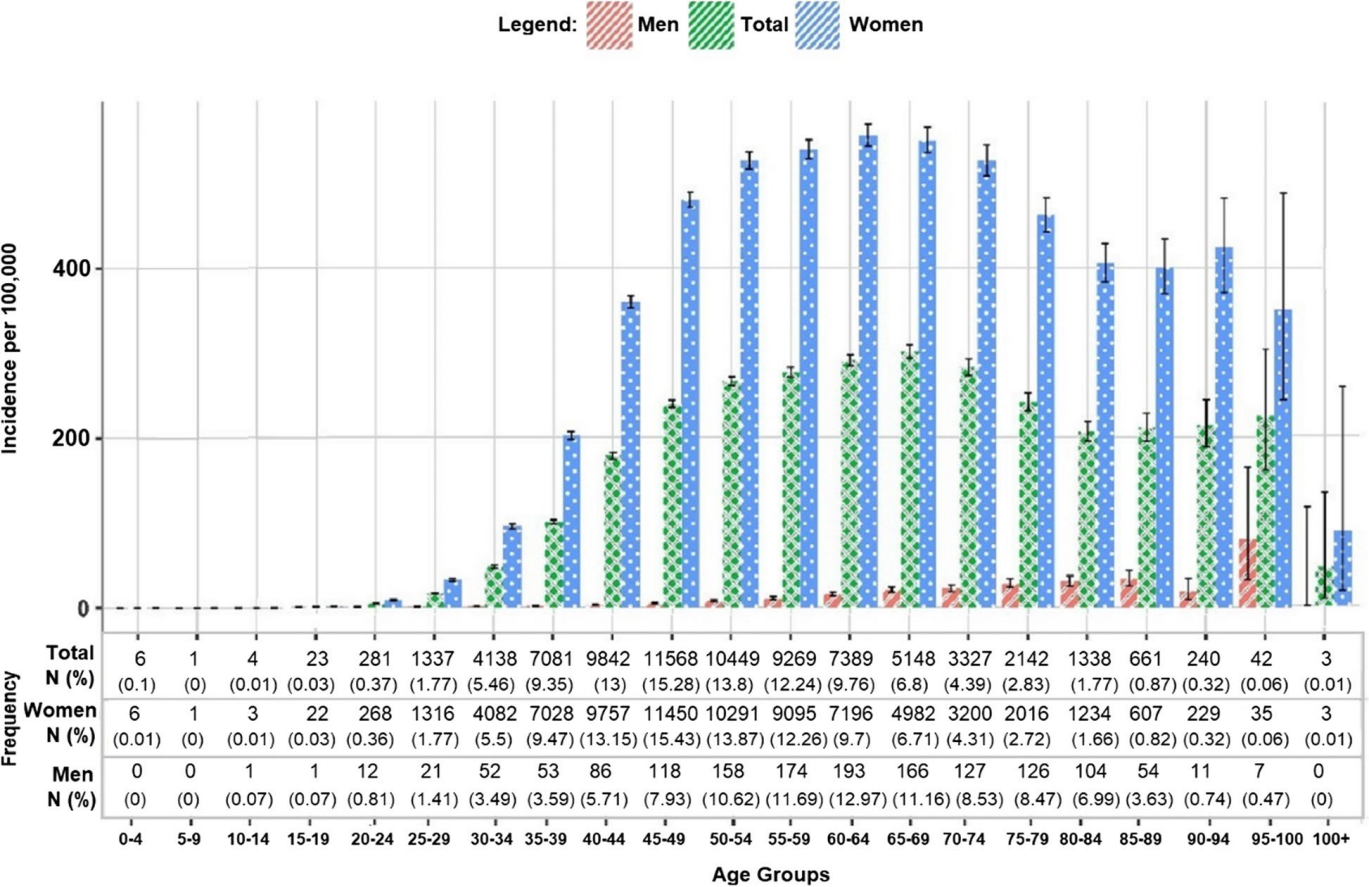
Age-specific breast cancer incidence rates per 100,000 by sex in Iran (2014–2018)

There was a strong correlation between age and BC incidence, with the highest rates among those aged 50–79 years (*p* < 0.05) (Fig. 3). The incidence rate per 100,000 women was consistently higher than for men across all age groups. The 65–69 age group had the highest incidence rate in the overall population at 301 cases per 100,000 (95% CI: 292.8–309.3). Women aged 60–64 years had the highest incidence at 556.6 per 100,000 (95% CI: 543.8–569.6). Men aged 95–99 years had the highest incidence at 80.4 per 100,000 (95% CI: 32.3–165.7). However, incidence rates for the 95–99 and over 100 age groups should be interpreted cautiously due to wide confidence intervals and small sample sizes. The incidence rate for men aged over 100 was unreliable due to a sample size of zero.

**Fig. 4:**
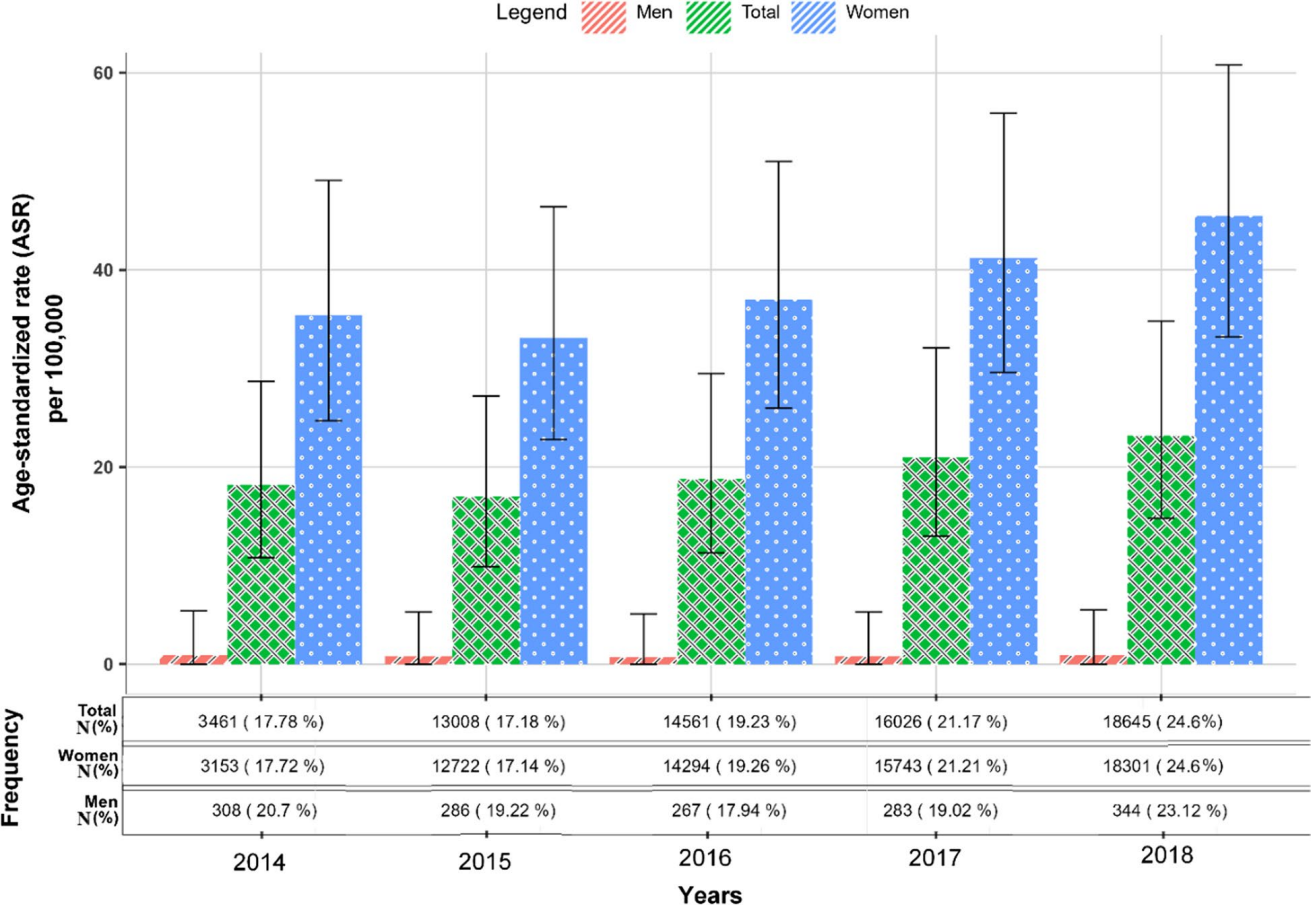
Age-standardized rate of breast cancer in Iran between 2014 and 2018, highlighting temporal trends and gender disparities in incidence

BC incidence increased over the study period, driven by a significant rise in rates among women while men's rates remained low and steady. The overall ASR increased from 18.2 (95% CI: 10.8–28.7) in 2014 to 23.2 per 100,000 (95% CI: 14.8–34.8) in 2018, indicating an alarming upward trend. Specifically, the ASR for women rose significantly from 35.4 (95% CI: 24.7–49.1) in 2014 to 45.5 per 100,000 (95% CI: 33.2–60.8) in 2018, a 28.5% increase. In contrast, the ASR for men remained relatively stable, ranging from 0.7 to 0.9 (Fig. 4).

**Fig. 5:**
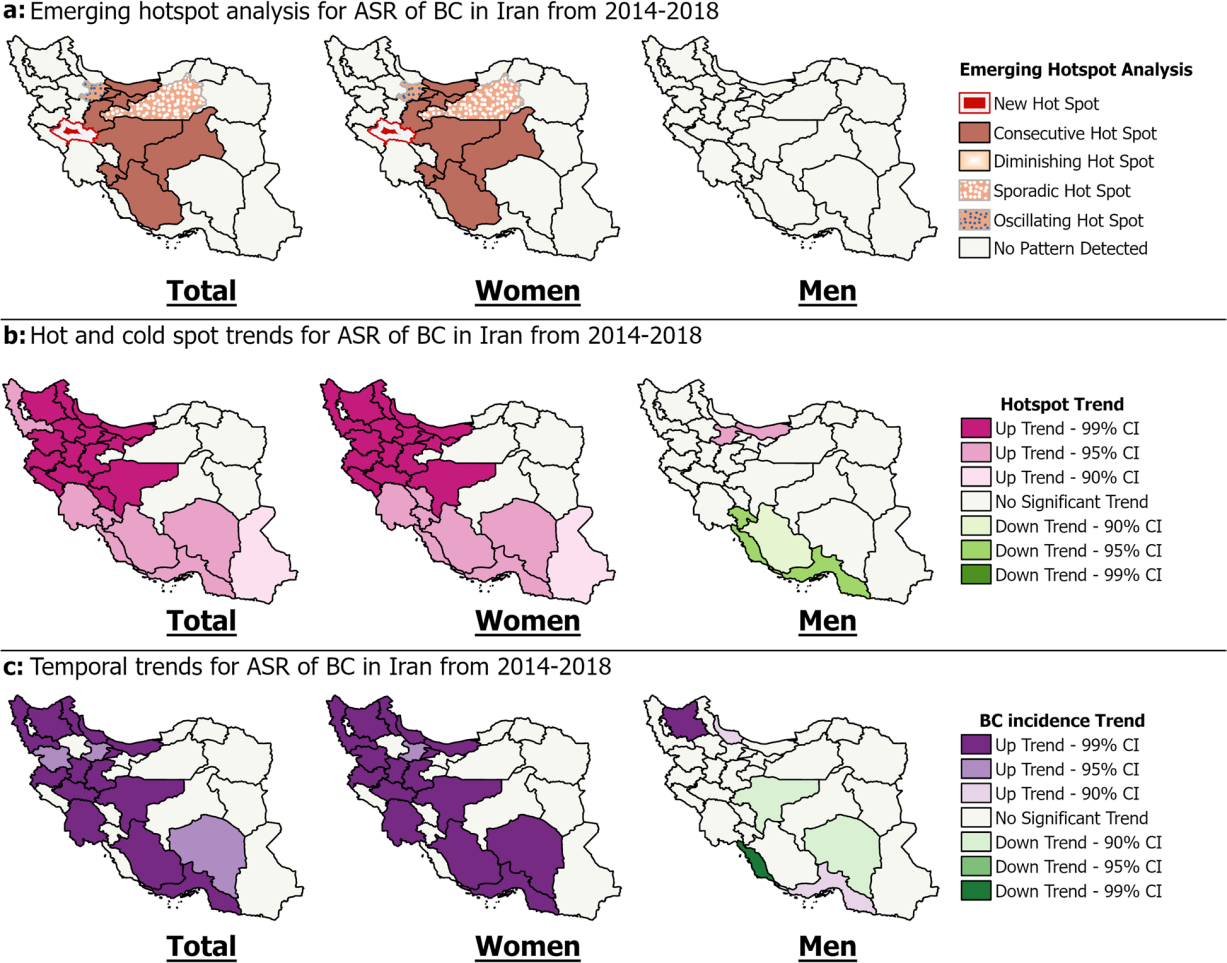
Spatiotemporal analysis of age-standardized rate of breast cancer by sex in Iran (2014–2018)

The spatiotemporal analysis revealed considerable geographical heterogeneity and temporal shifts in ASR of BC patterns among women across Iran's provinces from 2014–2018 (Fig. 5). The findings demonstrate substantial spatiotemporal variations in ASR of BC nationwide, overlaid on an overall upward trajectory. Several provinces emerged as persistent hotspots with high female incidence rates over time (Fig. 5a). For example, Isfahan and Tehran were classified as consecutive hotspots, indicating consistently high incidence. Lorestan was identified as a new hotspot, pointing to an emerging high-incidence area. The hot spot trend analysis using the Mann–Kendall test revealed statistically significant upward trends within existing hot spots (Fig. 5b). For instance, Isfahan province exhibited an intensifying hotspot, with a particularly sharp upward trend in ASR at 99% confidence, suggesting increasing cluster intensity over time. Meanwhile, provinces like North Khorasan showed no significant temporal cluster trends in ASR. Examining the temporal trajectories uncovered nuanced provincial-level shifts (Fig. 5c). Most provinces displayed statistically significant upward trends in female BC ASR from 2014–2018, with varying magnitudes. For men, while most provinces showed no significant hot/cold spot or ASR trends, exceptions like Qazvin and Mazandaran exhibited increasing hotspot intensity, and Ardabil an upward ASR trend over time.

**Fig. 6:**
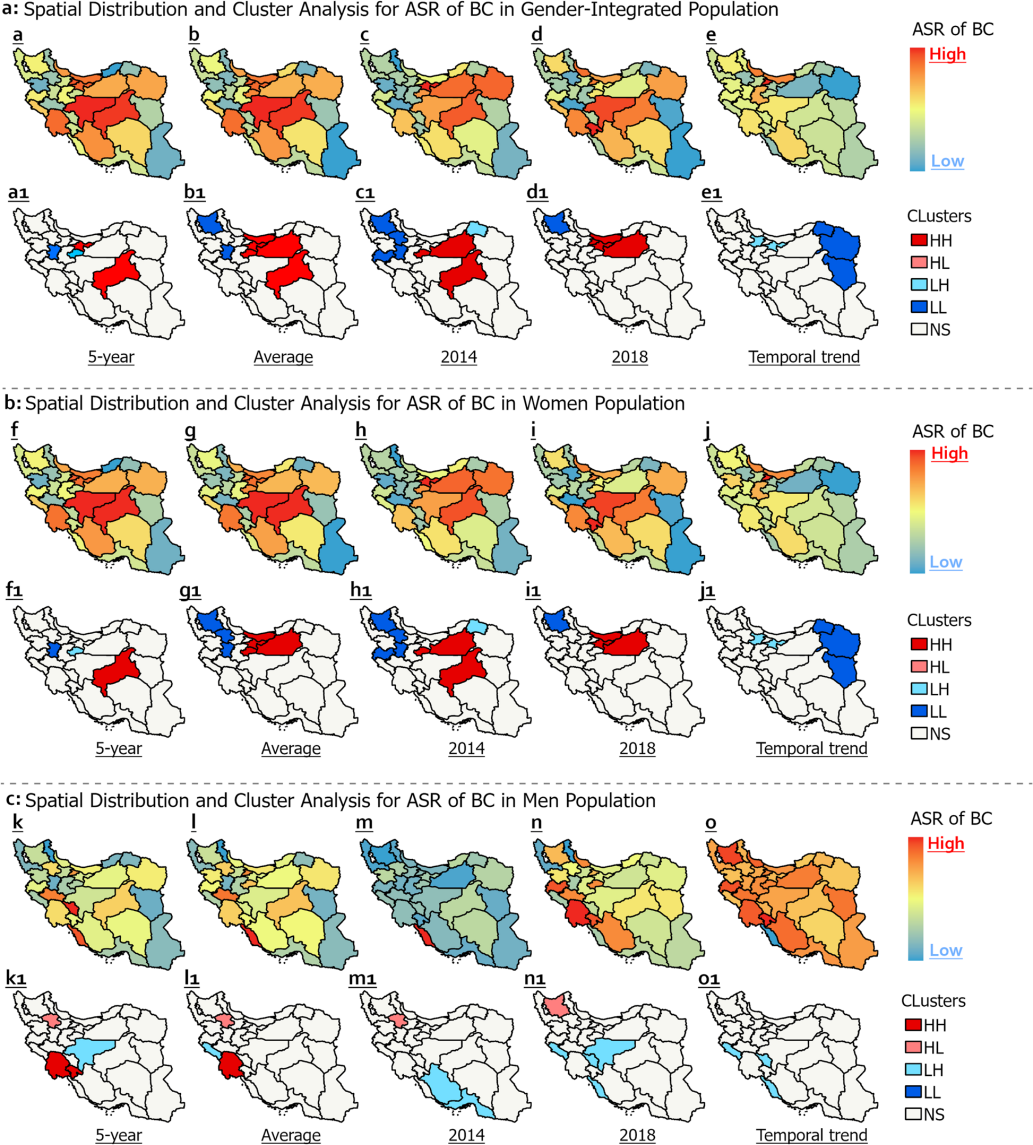
Geospatial and temporal analysis of age-standardized rate of breast cancer by sex in Iran (2014–2018)

In the entire population, BC rates clustered geographically, with central and northern Iran having higher incidence than other regions (Fig. 6a). Global Moran's I analysis revealed clustered BC patterns for the overall and female populations, while a random pattern was seen in men. Further details on these findings can be found in Supplementary file 5. Isfahan had the highest 5-year ASR (130.9), whereas Golestan had the lowest (35). Yazd and Tehran were high-high (HH) clusters, indicating higher rates than other provinces. From 2014–2018, Yazd, Semnan, Mazandaran, Tehran, and Qom were HH clusters. In 2014, Tehran had the highest ASR (27.4) and was an HH cluster along with Razavi Khorasan. In 2018, Yazd had the highest ASR (29.7) and Yazd, Semnan, and Qom were HH clusters. Alborz saw the largest ASR increase from 2014–2018 (+ 20.5).

In women, Isfahan Province had the highest 5-year ASR of 261 per 100,000, while Yazd Province was a HH cluster (Fig. 6b). Over five years, provincial ASRs ranged from 66.6 to 261 per 100,000 in women, with a national average of 153.5 per 100,000. Isfahan, Yazd, and Alborz Provinces exhibited the highest ASRs of 261, 260.2, and 245.6 per 100,000, respectively. Spatial analysis identified Semnan, Mazandaran, Tehran, and Qom as HH clusters, though most provinces lacked significant spatial patterns. Nevertheless, Tehran in 2014 and Alborz in 2018 had the highest ASRs. From 2014 to 2018, the ASR increased in 28 provinces and decreased in 3 provinces. Alborz showed the greatest increase at + 41.3 units, while Razavi Khorasan had the largest decrease at -11.8 units. Additionally, the spatial distribution of clusters changed over time, with Semnan exhibiting an HH cluster in 2014 and 2018.

In men, the ASR ranged from 1.7 in Ardabil to 7.8 per 100,000 in Chaharmahal and Bakhtiari province over the five years (Fig. 6c). Khuzestan, Chaharmahal and Bakhtiari provinces showed significant HH clusters for the 5-year ASR. Additionally, Khuzestan had an HH cluster for the average ASR from 2014–2018, which ranged from 0.32 in Ardabil to 1.5 in Bushehr. Regarding temporal trends, Bushehr had the largest decrease at -3.9 units, while Kohgiluyeh and Boyer-Ahmad had the greatest increase, rising from 0.4 per 100,000 in 2014 to 1.6 per 100,000 in 2018 (300% increase). Although some provinces exhibited sporadic ASR outliers from 2014–2018, no statistically significant spatial clustering was detected among men.

**Fig. 7:**
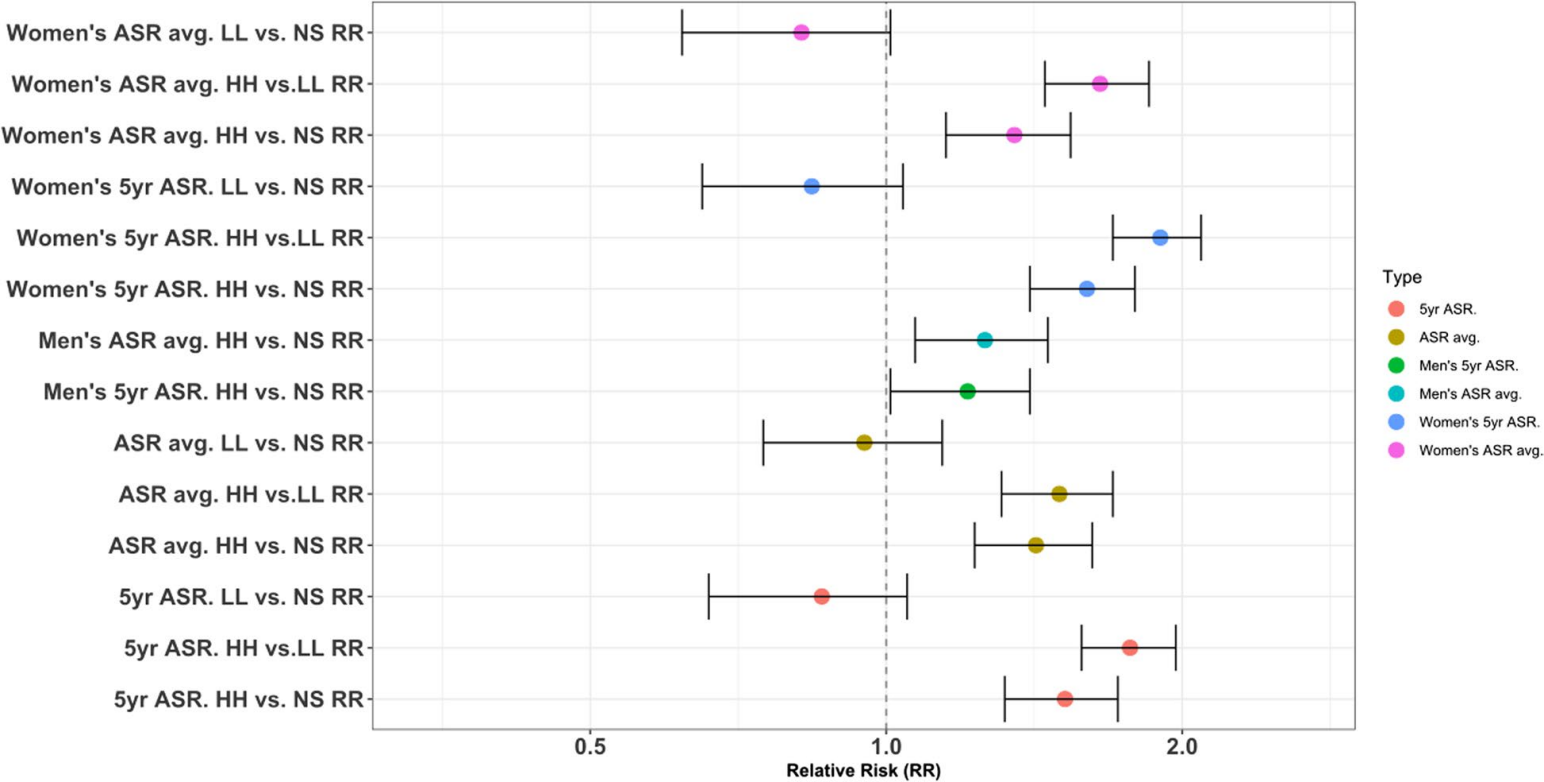
Breast cancer incidence relative risk in different spatial clusters in Iran (2014–2018). Abbreviations: ASR: Age-Standardized Incidence Rate, HH: High-High, LL: Low-Low, NS: Not Significant, avg: Average, yr: Year, RR: Relative Risk

Cluster analysis revealed individuals in HH clusters had significantly higher BC incidence compared to those in Low-Low (LL) clusters or non-significant (NS) provinces (*p* < 0.05) (Fig. 7). The 5-year ASR for the overall population was 1.77 times higher in HH versus LL clusters (95% CI: 1.58–1.97), and the average ASR was 1.5 times higher (95% CI: 1.31–1.7). For women, the 5-year and average ASRs were 1.89 (95% CI: 1.7–2.09) and 1.65 (95% CI: 1.45–1.85) times higher in HH clusters. BC incidence was also significantly higher in HH versus NS provinces. The 5-year ASR was 1.52 times higher (95% CI: 1.32–1.72) and the average ASR was 1.42 times higher (95% CI: 1.23–1.60) in HH clusters. For men, the 5-year and average ASRs were 1.21 (95% CI: 1.01–1.4) and 1.26 (95% CI: 1.07–1.46) times higher in HH versus NS provinces (Fig. 7).

Four regression models were employed for predicting ASR of BC across Iran’s provinces including Ridge, explanatory, OLS, and stepwise. The R^2^ values indicated the variance in ASR predicted by the input features (Table 2). All models significantly predicted ASRs for women and overall populations, with R^2^ from 0.516 to 0.799. However, models for men were not significant (R^2^ = 0.157 to 0.33). Ridge and explanatory regression produced the highest R^2^ values for women and overall (0.71 to 0.8), while OLS R^2^ ranged from 0.515 to 0.516. Stepwise regression identified four key predictors for the overall population including marriage, tertiary education, physician-to-population ratio, and PM2.5, while the top three predictors for women were marriage, tertiary education, and physician-to-population ratio. Stepwise regression R^2^ values ranged from 0.64–0.66 for women and overall populations. R^2^ in GWR model ranged from 0.55–0.56 for women and overall, indicating a moderate to strong goodness of fit in explaining outcome variance.

**Table 2:** Model specifications and feature selection for regression and geographically weighted regression in diverse datasets

Regression Model	Dataset	R^2^	Input feature (N)	output features (N)	Feature reduction N (%)
**Ridge**	Total	0.708	54	54	0 (0)
	Female	0.721	54	54	0 (0)
	Men	0.157	52	52	0 (0)
**Explanatory**	Total	0.788	54	22	32 (59)
	Female	0.799	54	22	32 (59)
	Men	0.343	52	30	22 (42)
**OLS**	Total	0.516	22	4	18 (81)
	Female	0.515	22	4	18 (81)
	Men	0.285	30	4	26 (86)
**Stepwise**	Total	0.658	14	4	10 (71)
	Female	0.637	14	3	11 (78)
	Men	0.155	14	4	10 (71)
**GWR**	Total	0.56	4	-	-
	Female	0.55	3	-	-

*N* Number, *OLS* Ordinary leaner regression, *GWR* Geographical weighted regression

**Fig. 8:**
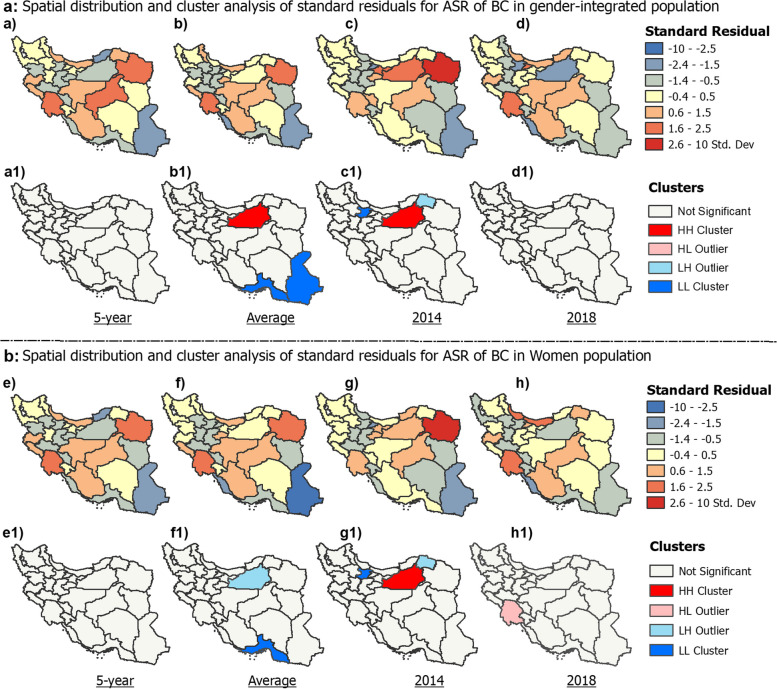
Geographically weighted regression model’s fit and spatial standard residual analysis for predicting ASR of breast cancer in Iran

GWR standard residuals and spatial cluster analysis assessed model goodness of fit and spatial variations in predicting ASRs of BC across provinces of Iran (Fig. 8). For the overall population, the GWR model included marriage state, physician-to-population ratio, educational level, and air pollution as predictor variables (Fig. 8a). For women, the model included marriage state, physician-to-population ratio, and educational level (Fig. 8b). Male breast cancer incidence was not modeled using GWR due to the low R-squared value (0.15) indicating poor model fit. Standardized residuals showed heterogeneity in predictor-ASR relationships by province and values outside the range of -2 to + 2 indicate locations poorly fit by the model. For the overall population, 5-year ASR standardized residuals ranged from -2.15 to + 2.38 SD, with the highest positive residual in Khuzestan, meaning the model underestimates the BC rate there. There are likely additional factors contributing to the high incidence rate that are not accounted for in the model. Provinces like Golestan with the lowest negative residuals have lower observed rates than predicted, suggesting model overestimation. This suggests factors included in the model are not strongly associated with BC incidence in these provinces. Semnan province displayed a HH cluster, meaning neighboring provinces also had highly positive residuals. This suggests geographic clustering of areas where the model underestimates breast cancer incidence. In contrast, Sistan and Baluchestan and Hormozgan provinces exhibited LL clustering, indicating geographic grouping of provinces where the model overestimates breast cancer rates. Similarly for women, Khuzestan had the highest positive residuals while provinces like Golestan had the lowest negative residuals. The large residuals in certain provinces indicate spatial heterogeneity and poor model fit, signaling a need for additional location-specific variables to improve performance.

No significant provincial clusters were detected in the 5-year standardized residual analysis, which represents the total ASR across the full 5-year study period. The lack of clustering implies there were no broad geographic patterns or spatial groupings among provinces with unusually high or low residuals for the complete 5-year ASR. This may be due to variable relationships between predictors and incidence rates over the full time period. The absence of residual clusters in the 5-year analysis underscores the complexity of modeling spatiotemporal breast cancer patterns over longer time frames. The lack of stable spatial clusters indicates substantial geographic heterogeneity in residual values for the 5-year ASR.

**Fig. 9:**
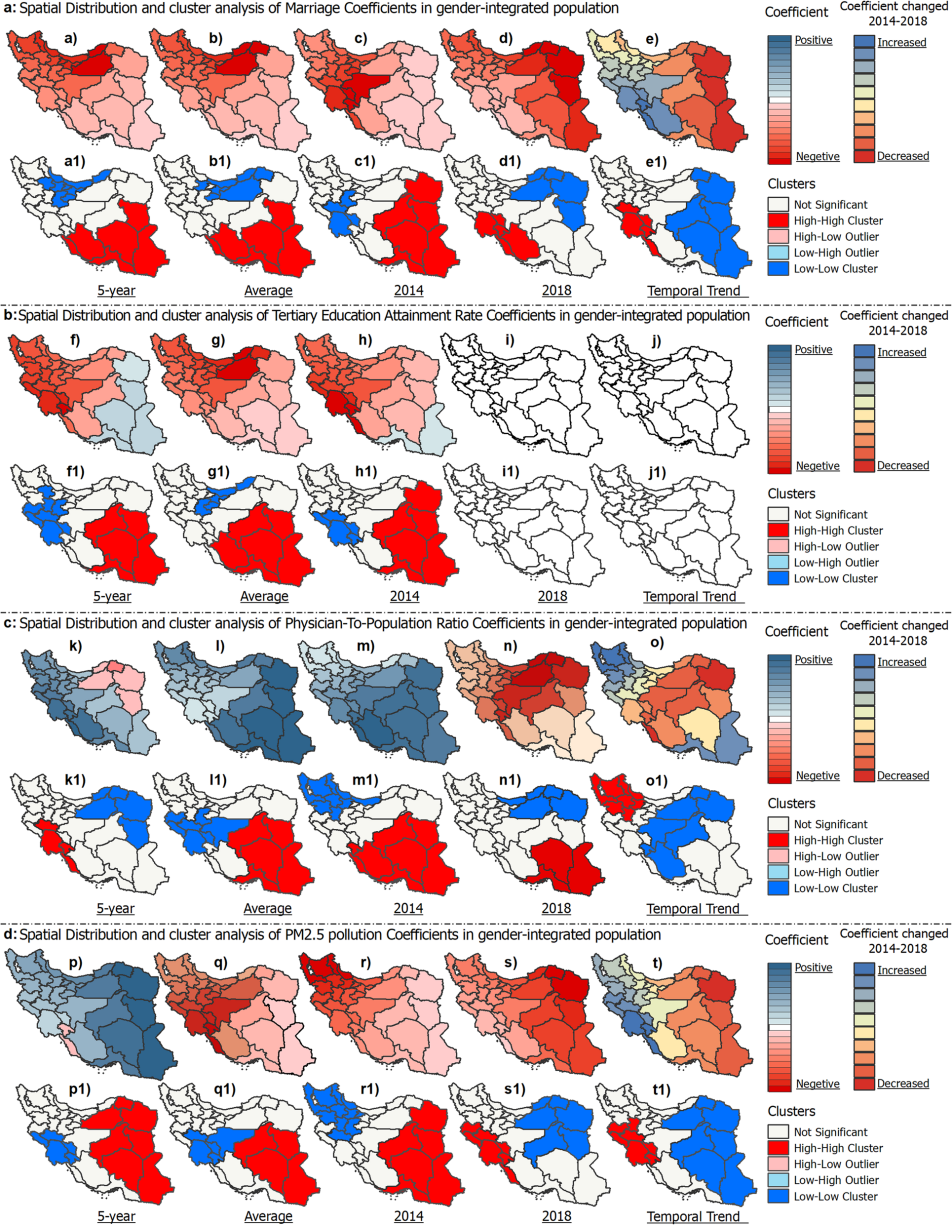
Spatiotemporal analysis of coefficients of multiple variables associated with ASR of breast cancer in gender-integrated population across provinces of Iran (2014–2018)

The coefficients for the predictors of marriage, education, healthcare access, and air pollution associated with ASRs of BC showed spatial variations across provinces in Iran from 2014–2018 (Fig. 9). For marriage, the coefficients ranged from -0.17 to -0.013 (Fig. 9a). The negative coefficients indicate that an increase in the number of marriages per 100,000 individuals was associated with a decrease in ASR. Tehran (-0.168) and Sistan and Baluchestan (-0.104) provinces exhibited significant LL and HH clustering of coefficients, respectively. This reflects differing magnitudes of the association between marriage rates and ASRs in the two provinces. For education, measured by tertiary education attainment, coefficients ranged from -0.011 to 0.018 (Fig. 9b). Several provinces showed HH clusters, indicating that increased education rates were associated with increased ASRs in those areas. For healthcare access, measured by physician-to-population ratio, coefficients ranged from -0.13 to 0.4 for 5-year ASR (Fig. 9c). The lowest coefficient was in North Khorasan (-0.09) and the highest was in Bushehr (+ 0.4). Khuzestan (LL cluster) and Sistan and Baluchestan (HH cluster) showed significant spatial clustering, reflecting geographic variations. For PM2.5 air pollution, coefficients ranged from -0.054 to 0.27 for 5-year ASR (Fig. 9d). Seven provinces had HH clusters and five provinces had LL clusters, indicating spatial variations in the magnitude and direction of the associations between PM2.5 and ASRs. Temporal changes were also seen in some provinces, including increased PM2.5 coefficients in Bushehr and decreased coefficients in Razavi Khorasan from 2014–2018.

**Fig. 10:**
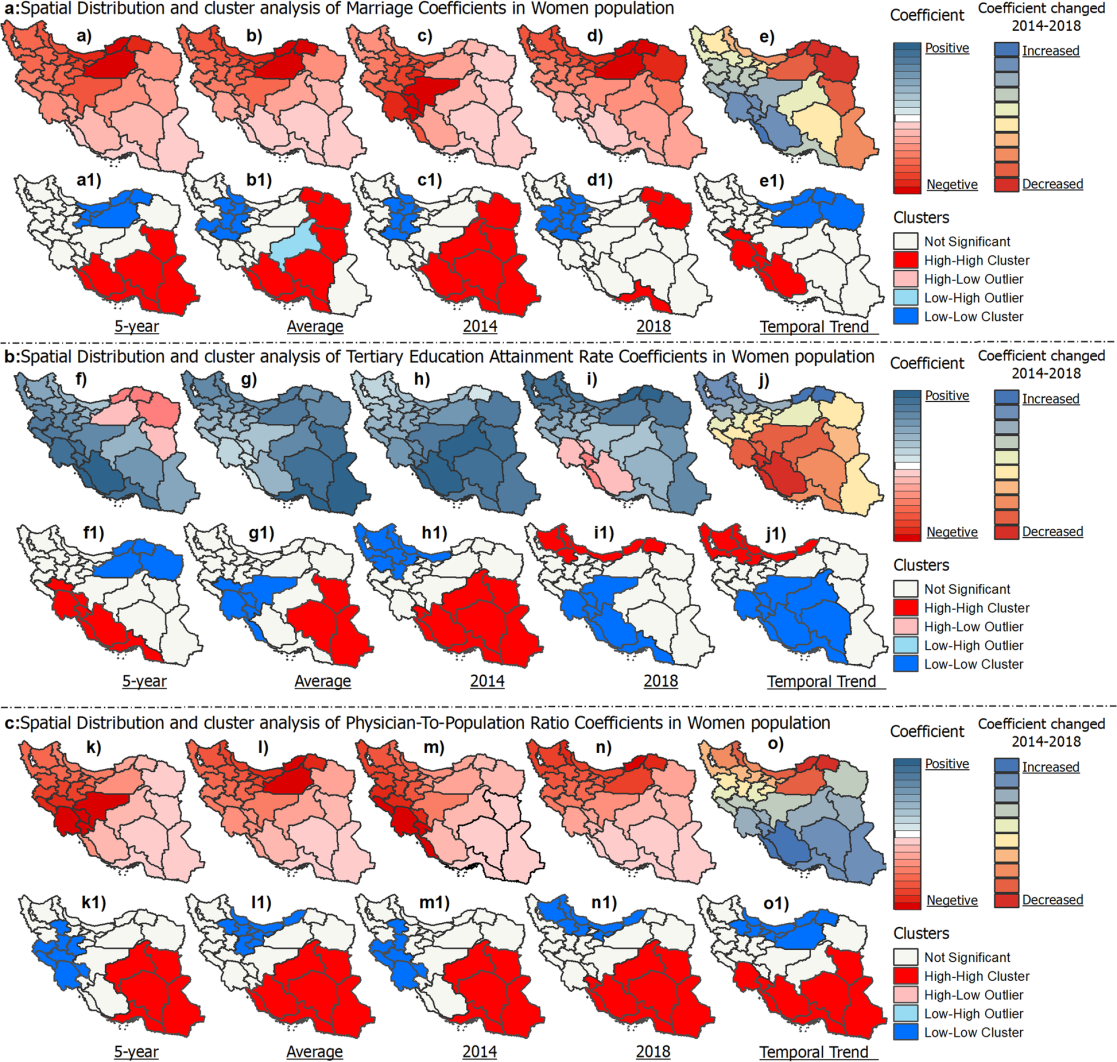
Spatiotemporal analysis of the coefficients of multiple variables associated with the ASR of breast cancer in women across the provinces of Iran (2014–2018)

The coefficients for marriage, education, and healthcare access associated with ASRs of BC in women showed spatial variations across Iran from 2014–2018 (Fig. 10). Marriage coefficients were mostly negative, ranging from -0.24 to -0.37 for 5-year ASR (Fig. 10a). Cluster analysis revealed HH clusters (lower magnitude associations) in six provinces and LL clusters (higher magnitude associations) in six provinces. Education coefficients ranged from -0.022 to 0.028 for 5-year ASR (Fig. 10b). Several provinces showed HH clusters, reflecting positive associations between education and ASRs. Average ASR education coefficients ranged from -0.011 to 0.002; seven provinces showed HH clusters and nine showed LL clusters. Healthcare access coefficients ranged from -0.59 to 0.72 for 5-year ASR, with most provinces showing positive correlations (Fig. 10c). Seven provinces exhibited HH clusters, indicating lower magnitude positive associations between physician density and BC incidence in women.

## Discussion

This spatial epidemiological study provides important new insights into the geographic distribution and regional determinants of BC incidence in Iran. By identifying high-risk clusters and elucidating key risk factors at the provincial level, our findings can facilitate targeted prevention, early detection, and control strategies tailored to the spatiotemporal variations observed. Furthermore, the policy insights from Iran's diverse subnational epidemiological profile may prove relevant for other populations confronting similarly high and increasing BC incidence globally.

### Incidence rate

This study found a 5-year ASR of BC in Iran of 98.5 overall, 192.5 in women, and 4.3 per 100,000 in men from 2014–2018, and disparate average annual rates of 19.6 for gender-integrated population, 38.4 for women, and 0.82 per 100,000 for men, consistent with higher global rates in women. Compared to neighboring countries, Iran's rates in women are higher than in Turkmenistan (32.9), Afghanistan (29.9) and Azerbaijan (34.7) but lower than in Iraq (52.7), in the United Arab Emirates (UAE) (58.5), Kuwait (50), Bahrain (44.2), and Turkey (46.6 per 100,000) [4, 28, 29]. The ASR of BC in Korea was higher than in Iran at 83 per 100,000, with invasive cancer and carcinoma in situ accounting for 68.5 and 14.5 cases, respectively [30]. Variations in BC incidence between developed and developing countries such as Iran likely stem from differences in risk factors, screening practices, and healthcare access [1]. The higher incidence rates observed in developed nations may be attributable to lifestyle determinants, whereas the lower rates in developing countries potentially reflect deficiencies in awareness, screening, timely diagnosis, and treatment facilities [3, 31]. Studies conducted in Ghana, Nigeria, and the UAE have identified barriers to BC screening including lack of awareness, limited geographic accessibility, fear, and cultural beliefs [32–34].

### Sex

Although primarily affecting women, BC also occurs in men, albeit at significantly lower incidence rates (38.4 in women vs 0.82 per 100,000 in men), affirming the importance of sex as a factor [35, 36]. This aligns with most prior studies [6–8, 35]. The biological differences between sexes play a vital role in BC development and progression. Women have more breast tissue and are exposed to estrogen, stimulating cell growth and raising BC risk. Women also have a higher prevalence of risk factors like sedentary lifestyles, late childbearing, and early menarche [8]. BC is rare but often diagnosed late in men, given less awareness and screening [6]. Risk factors include family history, genetic mutations, and radiation/estrogen exposure [8]. The stark variance in incidence highlights the need for sex-specific prevention and control strategies.

### Age-specific

BC incidence demonstrates regional variations in age distribution, with increasing rates among younger women globally. This study found peak incidence in Iran in ages 50–79, affirming a positive association between risk and age aligning with global data [7, 22, 37, 38]. A meta-analysis revealed the median age at diagnosis in Iran increased from 47.93 years in 2008 to 49.91 years in 2016 [37]. Iranian women had an average age of 50.9 years at diagnosis, consistent with prior studies [22]. The highest incidence rates in Iran were in ages 65–69 and 60–64 years, similar to patterns in Europe where the greatest rates occurred in women aged 65–69 [5, 39]. However, BC remains significant among younger women, with annual increases of 1.2% in European women aged 15–39 during 1990–2008, especially those under 35 [40]. While incidence peaks at older ages, rising rates among young women globally highlight the need for awareness and prevention across the lifespan.

BC incidence is rising among young women in Asia. In Korea, the peak incidence was aged 40–49 with a lower median diagnosis age than in Iran [30]. Japan saw bimodal peaks at 45–49 and 65–69 years [41]. Saudi Arabia's peak incidence was 70–74, though the median diagnosis age increased to 51 by 2017 [42]. In China and the US, incidence rose sharply with age, but China had an earlier peak age, more young patients, and more advanced stages [43]. Africa generally has lower rates than Iran, Europe, and North America [2]. In North America, the risk rises from 0.49% at 30 to 4.09% at 70 [44]. Rising incidence among younger women globally highlights the need for early screening, diagnosis, and targeted strategies across the life course to reduce the growing burden worldwide.

### Spatial incidence

This study found higher BC incidence rates in central and northern Iran compared to southern and eastern regions, with geographic variations between genders. Women clustered in central Iran while men clustered in the southwest. These spatial patterns align with previous research documenting higher incidence in central and northern regions [5, 22, 24, 45], especially Tehran, Isfahan, Mazandaran, and Yazd for women [24, 46]. Significant provincial disparities have been observed, with rates ranging from 5 to 72 per 100,000 across Iran [22]. Several factors may contribute: First, these regions may have more environmental carcinogens and other risk factors. Tehran, Semnan, and Mazandaran have high elderly population density, and various identified socioeconomic, reproductive, and lifestyle risk factors [23, 47]. Second, large industrial cities like Tehran, Yazd, and Mazandaran have well-established healthcare systems and screening enabling earlier detection and accurate reporting, potentially inflating rates compared to regions with limited access [48]. A US study found higher mammography facility density in white versus black neighborhoods, indicating access impacts reported rate, which was consistent [49].

Studies globally have found wide geographic variations in BC incidence and mortality rates which in line with this study. An Iraq study found higher incidence in Baghdad and Kirkuk provinces, with distinct high and low-risk districts but no clear urban–rural pattern [50]. A higher incidence was seen in Alaska, Southern, and Northern Plains among American Indian/Alaska Native women [10]. In Chile, higher crude mortality occurred in rural districts, though most deaths were urban [51]. In Colombia, lower mortality was associated with more forest area and fewer vehicles in spatial regression models [52]. Urban Israeli women had higher mortality than rural women, highlighting potential environmental factors and complex interactions [53]. Mexico analysis identified clustering of late-stage diagnoses in southern, central, and northern coastal regions beyond the main metro area [54]. In Spain, higher mortality in western provinces was possibly related to screening, reproductive factors, and age [55]. These studies demonstrate that geographic variations in BC epidemiology are complex and multifactorial, with relationships to healthcare access, environments, genetics, behaviors, and demographics. Tailored localized prevention and control initiatives should account for provincial disparities.

### Temporal trend

BC incidence rates in Iran exhibited an upward trajectory from 2014 to 2018, increasing from 18.2 to 23.2 per 100,000, mirroring rising regional and global trends [14]. The observed escalation in BC incidence in Iran, Central Asia, East Asia, and South Asia over recent decades underscores the necessity to comprehend these shifts within a broader context [13]. However, it is essential to consider that screening, diagnostic, and reporting enhancements such as expanded mammography and improved cancer registry practices can impact observed trends. Emerging hotspot analysis revealed heterogeneity in temporal provincial trends, with some provinces exhibiting disproportionate long-term BC burden potentially attributable to locale-specific risks. Consecutive hotspots like Alborz and Isfahan indicate enduring high-risk regions, while Lorestan’s designation as a new hotspot flags an emerging high-risk area necessitating research. Despite escalating BC incidence in Iran, screening and early diagnosis initiatives remain limited, with developing cancer registries and numerous advanced stage diagnoses, likely underestimating true incidence rates. The rising incidence despite constrained early detection resources underscores the urgent need to prioritize primary prevention targeting lifestyle and environmental risks, as well as strengthening healthcare systems for screening, diagnosis, treatment, and surveillance to mitigate the growing burden.

Examining specific regional examples reveals significant variations in BC incidence trends. In China, for instance, the ASR increased by 3.3% annually from 2000 to 2015, with a projected 11% increase by 2030 [56]. Iran experienced an annual BC incidence rise of 5.13% from 2004 to 2009 [46], and a dramatic increase in incidence from 15.96 to 40.72 per 100,000 women from 2003 to 2017 [5]. Neighboring countries like Turkey and Iraq also exhibit rising BC incidence rates [28, 29]. In Pakistan, the estimated adult BC incidence in Lahore was 76.8 per 100,000 from 2010 to 2019 [57]. Similarly, Korea witnessed a 5.6% annual increase in BC incidence from 1999 to 2019 [30]. While BC incidence rates in Africa are currently lower than in other regions, they have been rising rapidly since 1990 [14]. This increase may partially reflect lifestyle changes and improved registration, although underestimation is likely due to inadequate healthcare systems [58]. Europe's incidence increased since the 1950s but has slowed, as screening and awareness have increased detection while mortality declined in highly developed nations [59]. However, variations exist across Europe. In the Americas, patterns vary. The U.S. saw sharp rises until plateauing in the 2000s, attributable to increased screening starting in the 1950s [60]. Rates temporarily declined from 1999–2004 due to decreased hormone therapy use, before slightly rising again [61]. American Indian/Alaska Native incidence was stable from 1999–2015, while non-Hispanic whites declined and then increased [10]. Latin America and the Caribbean show slower increases than the U.S. and Europe [14, 62]. These consistent increases in BC incidence both globally and regionally underscore the urgency of addressing modifiable determinants and strengthening healthcare systems to facilitate prevention, early detection, and treatment, thus mitigating the growing burden of BC.

### Risk factors

BC has a complex, multifactorial etiology influenced by diverse genetic, lifestyle, hormonal, and environmental factors [63]. Lower SES is associated with higher mortality due to comorbidities, unhealthy lifestyles, less screening, and later diagnosis [64]. SES factors like income, education, occupation, and employment as well as ethno-cultural factors impact screening participation and diagnosis rates [11]. Poorer geographic access to facilities/screening and lower SES relate to later-stage diagnosis [65]. In Iran, higher BC incidence is seen in wealthier provinces, potentially reflecting urbanization, delayed childbearing, more screening, and better registries [23, 47]. Environmental exposures like light pollution and metals may contribute [7, 15], along with smoking, inactivity, obesity [7, 8], and reproductive factors like delayed childbearing, contraceptives, and reduced breastfeeding. Genetic factors like BRCA mutations significantly predispose to BC, highlighting the value of hereditary screening [8, 9]. A comprehensive analysis found family history confers around a twofold risk in Asian women, indicating key genetic factors across populations [66] This study identified marriage rates, education, physician density, and air pollution as key factors, aligning with prior evidence [15, 36, 67, 68]. However, other unexamined factors like diet, activity, stress, and healthcare access may also contribute. Incorporating more variables could elucidate spatial, cultural, and individual influences.

#### Marriage state

Prior studies have examined the association between marriage and BC incidence with conflicting results [13, 69–73]. Marriage has traditionally been linked to greater hormone exposure, potentially raising BC incidence risk [71]. However, this study found higher marriage rates associated with lower incidence in Iran overall. Tehran showed a stronger negative association, while Sistan and Baluchistan exhibited weaker negative associations, indicating geographic variations. The complex interplay between marriage and BC likely involves cultural and contextual factors warranting further research to elucidate protective mechanisms in the Iranian population. Leveraging these factors through culturally appropriate interventions could aid prevention. The protective effect of marriage against BC may stem from enhanced social and emotional support, influencing prognosis [73], though an Iranian meta-analysis found age at marriage 18–29 years increased risk [13]. Unmarried patients lack spousal support and may delay treatment [74]. In contrast, married patients benefit from financial stability and encouragement, potentially promoting timely treatment and improved survival [74]. Married patients more often receive optimal radiation, chemotherapy, and surgery [72, 73]. However, the marriage benefit varies by age, with more pronounced effects in older patients. Younger patients experience relationship strain and survival impacts from aggressive treatment and fertility concerns [75]. While mechanisms are unclear, marriage seems to confer social and economic benefits influencing BC outcomes in Iran, underscoring the need to consider cultural factors in prevention and control strategies.

#### Physician-to-population ratio

This study found physician availability has a complex association with BC outcomes. Regions with higher physician density have greater screening access and earlier diagnosis, resulting in more treatable stages and higher reported incidence. In contrast, lower physician regions have limited screening, potentially delaying diagnosis and lowering observed incidence despite advanced disease. Primary care physicians play key roles in prevention through education, counseling, and facilitating informed decisions [76]. However, physician-outcome associations vary geographically, indicating contextual influences on healthcare access [77]. Comprehensive strategies improving early detection through enhanced healthcare access, education, screening programs, and targeted physician supply investments in underserved areas are needed. Addressing healthcare resources, infrastructure, and access inequities through multifaceted policies is crucial to optimize BC outcomes.

#### Educational level

This study found a complex association between educational attainment and BC incidence in Iran, with higher education levels correlating with increased incidence in high-risk clusters yet decreased incidence in low-risk clusters. Prior meta-analyses have reported similarly contradictory results, with one study finding higher BC risk among more educated women but another showing no significant association [68]. Education may also improve outcomes, reducing incidence and mortality in some settings [78]. Greater educational attainment often enables higher income, improved healthcare access, and increased screening utilization. However, it may also associate with augmented BC risk factors like obesity, potentially offsetting benefits [67, 68]. Studies conducted in Pakistan, UAE, and Brazil indicate higher knowledge levels correlate with more education, while lower literacy relates to increased mortality [31, 34, 79]. Cultural attitudes and beliefs surrounding BC likely contribute to the varied geographic associations observed [16]. Overall, education appears to be an important yet multidimensional factor in BC epidemiology. Targeted interventions should leverage the benefits of education while also addressing lifestyle risks and cultural barriers to optimize prevention efforts and patient care. A nuanced understanding of education's complex role can inform localized public health strategies.

#### Air pollution

The relationship between PM2.5 air pollution and BC incidence is likely complex, with potential modulation by regional variations, lifestyle factors, genetics, and healthcare access. In the present study, certain Iranian provinces with high PM2.5 levels exhibited increased BC rates, while others showed decreased or inconsistent correlations. While PM2.5 contains recognized carcinogenic components, the precise biological mechanisms underlying its effects appear to be multifaceted and require further elucidation through research. Prior epidemiological studies have established associations between air pollution exposure and increased BC risk, with this relationship modulated by factors such as genetics, ethnicity, diet, and cultural context. Particulate matter components PM10 and PM2.5, in addition to gaseous pollutants like NO2 and SO2, have been linked to heightened BC incidence and mortality, particularly in highly polluted Chinese cities [80, 81]. PM2.5, NO2, and traffic-related emissions may act as mammary carcinogens [15, 82]. A study in Mexico found significant geospatial clustering of BC cases near pollution sources including refineries and industrial facilities [83]. However, the associations with PM are complex; specific components like nickel and vanadium, rather than overall PM levels, may drive the increased risk [73–75]. Genetic variations, ethnicity, dietary patterns, and cultural differences also appear to influence the effects of pollution [82]. Overall, the body of evidence supports ambient air pollution as a risk factor for BC, underscoring the need for mitigation strategies such as emissions control policies, green initiatives, and protective equipment to reduce exposures.

### Feature selection

Accurate feature selection is critical for identifying the most relevant variables from large cancer data sets [19]. Different approaches exist, including filter methods based on statistical measures like correlation, wrapper methods evaluating model performance with different feature combinations, and embedded methods incorporating selection into model-building [18]. This study utilized regression as a wrapper method for feature selection in BC modeling. Regression techniques can assess the contribution of each variable to model performance, facilitating systematic selection of the most impactful features. Employing robust feature selection maximizes model accuracy while minimizing overfitting, supporting generalizability and clinical applicability. Regression techniques offer advantages for feature selection but also limitations. Advantages include quantifying feature contributions, accommodating nonlinear relationships, and managing multicollinearity. However, they assume linearity and correct model specification, which may not hold and can be computationally intensive [84].

### Spatial analysis

This study utilized spatial analysis techniques including global and local spatial autocorrelation, spatial clustering, Emerging hotspot analysis, and GWR to examine BC incidence patterns and associated risk factors in Iran. These methods align with previous spatial epidemiology research [22, 24, 46] and leverage advancements in geospatial technology to investigate how spatial factors like healthcare access and demographics influence disease distribution [15, 65]. Global and local Moran's I tests assessed spatial autocorrelation to identify high or low-incidence clusters [22]. Emerging hotspot analysis provided profound insights into the intricate spatiotemporal variations in BC epidemiology across Iran's provinces. GWR modeling captured small-area variations missed by global regressions [15, 17]. Results revealed provincial differences in ASRs of BC, with some provinces exhibiting higher rates than expected and others lower. While GWR has limitations like assuming spatial consistency of relationships [17], it remains valuable for studying spatial variations in incidence. These findings demonstrate the importance of accounting for spatial heterogeneity in cancer analysis, to which GWR can be a useful approach.

### Limitations

This study has several limitations that should be considered. First, data availability presented challenges. The use of aggregated cancer registry data meant individual-level clinical parameters could not be assessed. Access to more granular data on screening, diagnostics, treatment, and outcomes could strengthen the analysis of geographic patterns. Additionally, under-reporting in rural areas may underestimate the true incidence. Efforts to improve cancer surveillance systems could enable more robust epidemiological research. Second, the ecological design precludes causal inference due to ecological fallacy. Individual-level case–control or cohort studies are needed to clarify biomedical and behavioral risk factors. The cross-sectional nature also limited causal assessment, highlighting the need for longitudinal designs tracking exposures and outcomes over time. Third, generalizability may be restricted given the sole focus on Iran's context. Replication across diverse populations would clarify generalizable versus setting-specific findings. However, detailed descriptions of the study population and setting were provided to assist readers in assessing applicability to their contexts. Fourth, the analysis was limited to available variables in the datasets. Incorporating additional parameters such as detailed environmental exposures, healthcare system factors, and genetic markers could strengthen future modeling. The availability of high-dimensional omics data could also enable more advanced bioinformatic analysis. Finally, this study utilized observational data, restricting causal conclusions. Experimental designs manipulating potential risk factors would provide higher-level evidence on etiology and prevention. However, observational methods remain valuable for elucidating real-world patterns and generating hypotheses for future experimental testing.

#### Recommendation

Future research could expand on this study's findings by investigating additional risk factors for BC incidence using various feature selection and spatial analysis techniques. For instance, lifestyle, environmental, and genetic variables could be examined. Integrating more variables may enable more accurate identification of high-risk regions and potential BC determinants, while comparing efficacy and limitations of approaches. Moreover, an in-depth investigation of the risk factors identified here, particularly in high-risk areas, could better elucidate relationships with BC incidence to develop more reliable predictive models. Such efforts would assist developing targeted interventions to reduce the BC burden in high-risk regions by providing valuable insights to policymakers and public health officials.

## Conclusion

This study identified high-risk spatial clusters of BC incidence in certain provinces of Iran, with significant inter-regional heterogeneities in BC incidence rates. Key determinants associated with elevated risk encompassed marriage density, educational attainment, physician density, and PM2.5 air pollution. Early detection of BC enables more efficacious therapeutic interventions, underscoring the necessity for health literacy campaigns regarding BC risks and screening modalities. Provinces exhibiting elevated incidence should be targeted for tailored interventions addressing the elucidated risk factors. Provinces with lower incidence necessitate optimization of early diagnostic and therapeutic approaches. The findings of this study provide perspectives to inform evidence-based health policies and initiatives aimed at mitigating Iran's BC burden. Policymakers should consider high-risk clusters and contiguous areas in formulating comprehensive geographical prevention strategies. Further research and concerted efforts are warranted to address modifiable risk factors and the global BC burden.

### Supplementary Information

**Additional file 1.**

**Additional file 2.**

**Additional file 3.**

**Additional file 4.**

**Additional file 5.**

## Data Availability

The datasets examined in this study can be accessed from four distinct repositories. However, it should be noted that the availability of two repositories, namely the Iran National Population-Based Cancer Registry (INPCR) and the Air Pollution Monitoring System of Iran (APMS), are subject to regional restrictions. These restrictions determine that access to the data is limited to users within Iran only. The data concerning BC counts was obtained from the INPCR, which operates under the administration of the Ministry of Health and Medical Education in Iran. The INPCR's website can be found at [https://cancer.behdasht.gov.ir/]. Unfortunately, users outside of Iran may encounter difficulties in accessing this valuable data resource due to regional limitations. For data related to population, healthcare infrastructure, and the environment, two repositories were utilized. The first repository is The Statistical Center of Iran (SCI), which provides comprehensive information and can be accessed globally at [https://www.amar.org.ir/]. The second repository, the National Office for Civil Registration (NOCR), offers global access to this dataset, allowing researchers worldwide to benefit from the information. It can be accessed at [https://www.sabteahval.ir/]. Furthermore, the air quality data was sourced from the APMS. This repository is available at [https://aqms.doe.ir/]. However, only researchers within Iran can utilize this repository to analyze and assess the air quality in the country. It is crucial to acknowledge that the limitations on accessing certain repositories are beyond the control of the researchers conducting this study. These limitations stem from regional restrictions that affect the availability of data to a global audience.

## Abbreviations

BC: Breast cancer
SES: Socioeconomic status
GIS: Geographical information system
INPCR: Iran national population-based cancer registry
SCI: Statistical center of Iran
NOCR: National office for civil registration
APMS: Air pollution monitoring system of Iran
ICD-O: International classification of disease for oncology
CO2: Carbon monoxide
O3: Ozone
NO2: Nitrogen dioxide
SO2: Sulfur dioxide
PM10: Particulate matter of size ≤ 10 microns
PM2.5: Particulate matter of size ≤ 2.5 micron
AQI: Air quality index
ASR: Age-standardized incidence rate
VIF: Variance inflation factor
OLS: Ordinary least squares
RR: Relative risks
GWR: Geographically weighted regression
SR: Standard residuals
CI: Confidence interval
HH: High-high
LL: Low-low
HL: High-low
LH: Low–high
NS: Not significant
N: Number
UAE: United Arab Emirates
