# Supplementary material for: Exploring the geospatial epidemiology of breast cancer in Iran: identifying significant risk factors and spatial patterns for evidence-based prevention strategies
Source: BMC Cancer. 2023 Dec 11;23:1219. doi: 10.1186/s12885-023-11555-1 (PMC10712175; doi:10.1186/s12885-023-11555-1)
Supplement: Supplementary file 1 — Additional file 1. [file 12885_2023_11555_MOESM1_ESM.docx]

Explanatory Variables and Data Sources for Feature Selection in Breast Cancer Geospatial Epidemiological Analysis in Iran

| Category | Variable | Measure and Unit | Data Source |
| --- | --- | --- | --- |
| Cancer-related Variables | BC count (V1) | The annual incidence of newly diagnosed cases of breast cancer within a population over one year. | INPCR |
|  | The crude Incidence rate in 100,000 population (V2) | The incidence rate of newly diagnosed cases of breast cancer per 100,000 individuals within the population at risk. (Number of new cases/Total population) * 100000 | - |
|  | The age-Standard rate in the 100,000 population (V3) | Σ (standardized rate * proportion of the population in each age group), Σ represents the sum over all age groups. | - |
|  | The mortality rate in the 100,000 population (V4) | The mortality rate of deaths from a specific cause within a defined population in one year. (Number of deaths/Total population) *100000 | NOCR |
|  | The cancer Mortality rate in the 100,000 population (V5) | The cancer mortality rate within a specific population during one year represents the frequency of deaths caused by cancer. (Number of cancer deaths/Total population) *100000 | NOCR |
| SocioDemographic Variables | Population Density (V6) | Total population/neighborhood area (km2) = persons/km2. | SCI |
|  | Age Group Population rate in % (V7) | The proportion of a specific age group within the total population, is expressed as a ratio or percentage. (Age group) population/total population * 100 (%) | SCI |
|  | Elderly rate in % (V8) | The proportion of elderly individuals (usually defined as those aged 65 and older) within the total population, is expressed as a ratio or percentage. = elderly  (65+ years) population/total population * 100 (%) | - |
|  | Province Area in km2 (V9) | The geographic extent or land area of a province or administrative region, typically measured in square kilometers or other appropriate units of area. Province area/Total number of square kilometers | SCI |
|  | Household Density (V10) | The spatial density of household units, expressed as the number of households per square kilometer (km^2), within a specific geographic area. | SCI |
|  | The birth rate in the 100,000 population (V11) | The frequency of live births, expressed as the number of births per 1,000 individuals in a specific population, occurring within one year. (Number of live births/Total population) * 100000 | NOCR |
|  | The marriage rate in the 100,000 population (V12) | The frequency of marriages within a specific population (+10 years) over one year, expressed as the number of marriages per 1,000 individuals in the population, (Number of marriages/Total population) * 100000 | NOCR |
|  | The divorce rate in the 100,000 population (V13) | The frequency of divorces within a specific population over one year, (Number of divorces/Total population) * 100000 | NOCR |
|  | The fertility rate in 100,000 Women (V14) | The number of live births per 100,000 women of reproductive age (typically between 15 and 49 years old) in a specific geographic area over one year, (Number of live births/Number of women aged 15-49) * 100,000 | SCI |
|  | Urbanization rate in % (V15) | The proportion of the total population living in urban areas, expressed as a percentage, (Number of people living in urban areas/Total population) x 100 | SCI |
|  | Economic Partnership rate in % (V16) | The percentage of the population aged 10 years and older who are employed in the private sector or are self-employed, (Number of people employed in the private sector or self-employed/Total population) x 100 | SCI |
|  | The illiteracy rate in 100,000 (V17) | The ratio of illiteracy in the total population aged 6 years and older = illiterate population/population (6+) * 100000 | SCI |
|  | Vehicle Density rate in 100,000 population (V18) | The number of registered vehicles (including cars, trucks, and motorcycles) per 100,000 population within one year, (Number of registered vehicles/Total population) * 100,000 | SCI |
|  | Tamin Insurance rate in % (V19) | The proportion of a province's population covered by Tamin insurance, a social security system in Iran, expressed as a percentage of the total population, (Number of people covered by Tamin insurance/Total population) x 100 | SCI |
|  | Salamat Insurance rate in % (V20) | The proportion of a country's population covered by Salamat insurance, a health insurance scheme in Iran, expressed as a percentage of the total population, Number of people covered by Salamat insurance/Total population) x 100 | SCI |
|  | Ashayer Insurance rate in % (V21) | The proportion of a country's population covered by Ashayer insurance, a social security system in Iran specifically designed for nomads and tribal people, expressed as a percentage of the total population, (Number of people covered by Ashayer insurance/Total population) x 100 | SCI |
|  | Tertiary education attainment rate in % (V22) | The proportion of a country's population who have completed at least a tertiary education degree is expressed as a percentage of the total population. | SCI |
| Healthcare Infrastructure Variables | Hospital density in 100,000 population (V23) | The density of hospitals, expressed as the number of hospitals per 100,000 people in a specific geographic area, over a specified period, (Number of hospitals/Total population) * 100,000. | SCI |
|  | Inpatient Bed Count density in 100,000 population (V24) | The density of hospital inpatient beds, expressed as the number of beds per 100,000 people in a specific geographic area, over a specified period, (Number of inpatient beds/Total population) * 100,000 | SCI |
|  | Primary Care density in the 100,000 population (V25) | The density of primary care facilities, such as family doctors or general practitioners, expressed as the number of facilities per 100,000 people in a specific geographic area, over a specified period, (Number of primary care facilities/Total population) * 100,000 | SCI |
|  | Nuclear Medicine density in the 100,000 population (V26) | The density of nuclear medicine facilities, expressed as the number of facilities per 100,000 people in a specific geographic area, over a specified period, (Number of nuclear medicine facilities/Total population) * 100,000 | SCI |
|  | The physician-to-population ratio in the 100,000 population (V27) | The density of physicians, encompassing general practitioners and specialists, is expressed as the number of physicians per 100,000 people in a specific population or geographic area, over a specified period | SCI |
|  | Clinic Density in the 100,000 population rate in 100,000 population (V28) | The density of clinics, expressed as the number of clinics per 100,000 people in a specific population or geographic area, over a specified period, (Number of Clinics/Total population) * 100,000 | SCI |
|  | Emergency Care Facilities Density rate in the 100,000 population (V29) | The density of emergency care facilities, expressed as the number of facilities per 100,000 people in a specific population or geographic area, over a specified period, (Number of Emergency Care Facilities/Total population) * 100,000 | SCI |
|  | Rehabilitation Center Density in the 100,000 population (V30) | "The density of rehabilitation centers, expressed as the number of facilities per 100,000 people in a specific population or geographic area, over a specified period, (Number of Rehabilitation Centers/Total population) * 100,000 | SCI |
|  | Laboratory Density in the 100,000 population (V31) | The density of laboratories, expressed as the number of facilities per 100,000 people in a specific population or geographic area, over a specified period, (Number of Laboratories/Total population) * 100,000 | SCI |
|  | Pharmacy Density in the 100,000 population (V32) | The density of pharmacies, expressed as the number of facilities per 100,000 people in a specific population or geographic area, over a specified period, (Number of Pharmacies/Total population) * 100,000 | SCI |
| Environmental Variables | Urban Gini Index (V33) | Gini coefficient, a measure of income inequality in urban areas, is calculated as 2(A)/n(n-1), where A represents the area between the Lorenz curve (a cumulative distribution of income) and the perfect equality line, and n is the total number of income earners. The Gini coefficient ranges from 0 (representing perfect equality) to 1 (representing perfect inequality). | SCI |
|  | Rural Gini Index (V34) | A measure of income inequality in rural areas, same as V33 | SCI |
|  | Elevation in meter (V35) | The elevation or altitude of a specific location above average sea level, measured in meters. | SCI |
|  | Maximum Temperature Average (V36) | The average maximum temperature, expressed in degrees Celsius, averaged over a specific period (°C) | SCI |
|  | Minimum Temperature Average (V37) | "The average minimum temperature, expressed in degrees Celsius, averaged over a specific period (°C). | SCI |
|  | Temperature Average (V38) | The average temperature, expressed in degrees Celsius, averaged over a specific period (°C). | SCI |
|  | Minimum Low Temperature (V39) | The minimum temperature recorded over a specific period, measured in degrees Celsius (°C). | SCI |
|  | Maximum High Temperature (V40) | The maximum temperature recorded over a specific period, measured in degrees Celsius (°C). | SCI |
|  | Yearly Precipitation (V41) | The annual precipitation, including both rainfall and snowfall, measured in a given area or region | SCI |
|  | Maximum Daily Precipitation (V42) | The maximum daily precipitation, including both rainfall and snowfall, is measured in millimeters within 24 hours in a given area or region (mm). | SCI |
|  | Yearly Precipitation Average (V43) | The average precipitation, including both rainfall and snowfall, is measured in millimeters over a specific period, typically a year or a month, in a given area or region (mm). | SCI |
|  | Maximum Average Relative Humidity (V44) | The maximum level of relative humidity, expressed as a percentage, averaged over a specific period (e.g., a month or a year) in a particular location or geographic area (%), (Sum of all hourly relative humidity readings in a period)/(Number of hourly readings in that period | SCI |
|  | Minimum Average Relative Humidity (V45) | The minimum level of relative humidity, expressed as a percentage, averaged over a specific period (e.g., a month or a year) in a particular location or geographic area, (Sum of all hourly relative humidity readings in a period)/(Number of hourly readings in that period) | SCI |
|  | Days With Frost (V46) | The count of days with a minimum temperature below the freezing point of water, typically 0°C (32°F), within a specific location or geographic area over a certain period. | SCI |
|  | Days With Dust (V47) | The count of days with a high level of suspended dust particles in the air within a specific location or geographic area over a certain period. | SCI |
|  | Sunshine Duration (V48) | The duration of visible sunlight during daylight hours in a specific location, measured as the period between sunrise and sunset, excluding periods when the sun is obscured by clouds or other factors | SCI |
|  | Maximum Wind Speed (V49) | The highest recorded wind speed over a certain period in a specific location, measured in meters per second | SCI |
| Air Quality Variables | Carbon Monoxide (CO2) (V50) | The concentration of carbon monoxide in the air in a specific location, measured in parts per million (ppm), is calculated as the mass of CO in the air divided by the volume of air sampled (ppm) | APMS |
|  | Ozone (O3_1) (V51) | The concentration of ozone at a specific height above the ground in a specific location, typically measured in parts per billion (ppb), calculated as the volume of ozone divided by the volume of air sampled | APMS |
|  | Ozone (O3) (V52) | The concentration of ozone (O3) at ground level in a specific location, typically measured in parts per billion (ppb), (mass of ozone at ground level)/(volume of air sampled) in parts per billion (ppb) | APMS |
|  | Nitrogen Dioxide (No2) (V53) | The measurement of nitrogen dioxide concentration in the ambient air, expressed in parts per billion (ppb) | APMS |
|  | Sulfur Dioxide (SO2) (V54) | The concentration of sulfur dioxide in the air, is measured in parts per billion (ppb) or micrograms per cubic meter (μg/m3). | APMS |
|  | Particulate matter of size ≤10  micron (PM10) (V55) | The concentration of particulate matter with a size of 10 microns or smaller in the air, is measured in micrograms per cubic meter (μg/m3). | APMS |
|  | Particulate matter of size ≤2.5 micron (PM2.5) (V56) | The concentration of particulate matter with a size of 2.5 microns or smaller in the air, measured in micrograms per cubic meter (µg/m3). | APMS |
|  | Air Quality Index (AQI) (V57) | Air Quality Index (AQI) is a scale used to communicate air quality levels to the public, based on the concentrations of multiple pollutants. It is represented as a number between 0 and 500, with higher values indicating worse air quality. | APMS |
