# Supplementary material for: Exploring the geospatial epidemiology of breast cancer in Iran: identifying significant risk factors and spatial patterns for evidence-based prevention strategies
Source: BMC Cancer. 2023 Dec 11;23:1219. doi: 10.1186/s12885-023-11555-1 (PMC10712175; doi:10.1186/s12885-023-11555-1)
Supplement: Supplementary file 2 — Additional file 2. [file 12885_2023_11555_MOESM2_ESM.docx]

# Detailed Results of Feature Selection in Geospatial Epidemiological Analysis of Breast Cancer in Iran

##
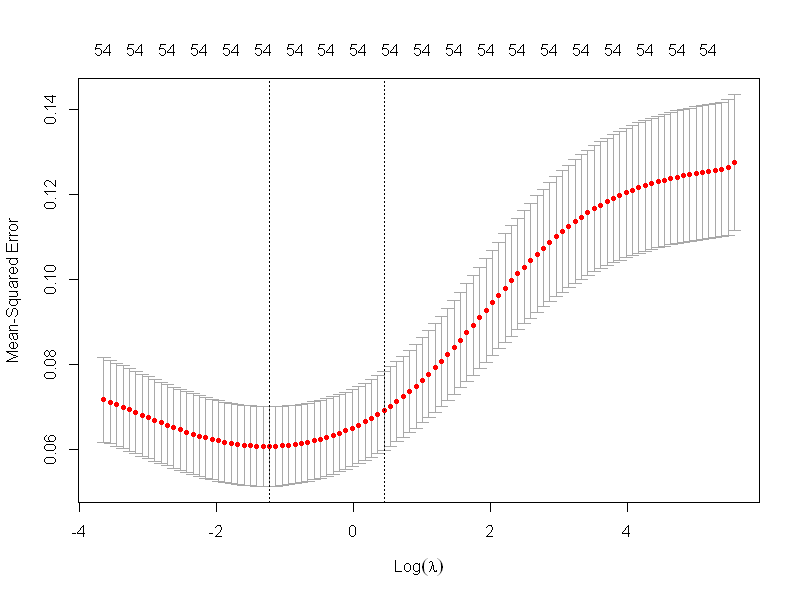

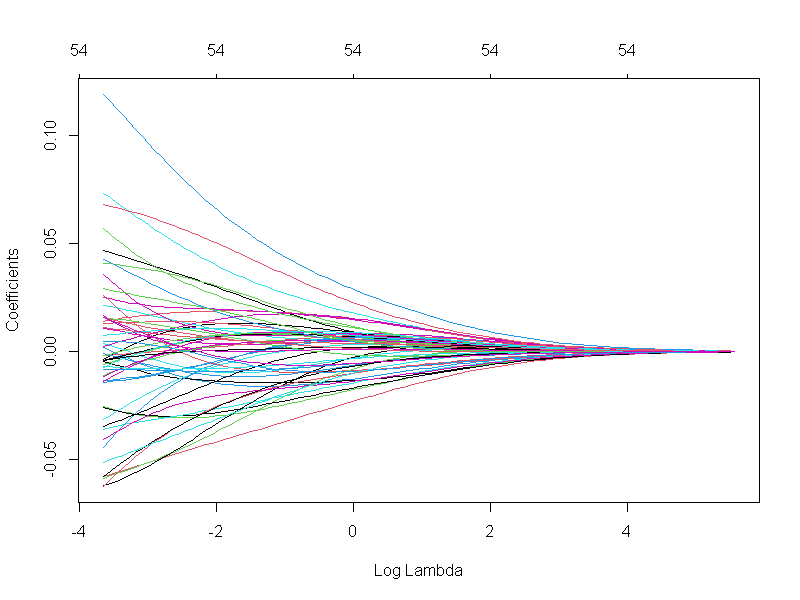
A: Feature Selection analysis results for gender-integrated population

### **A-1:** Ridge Regression Analysis Results for gender-integrated population


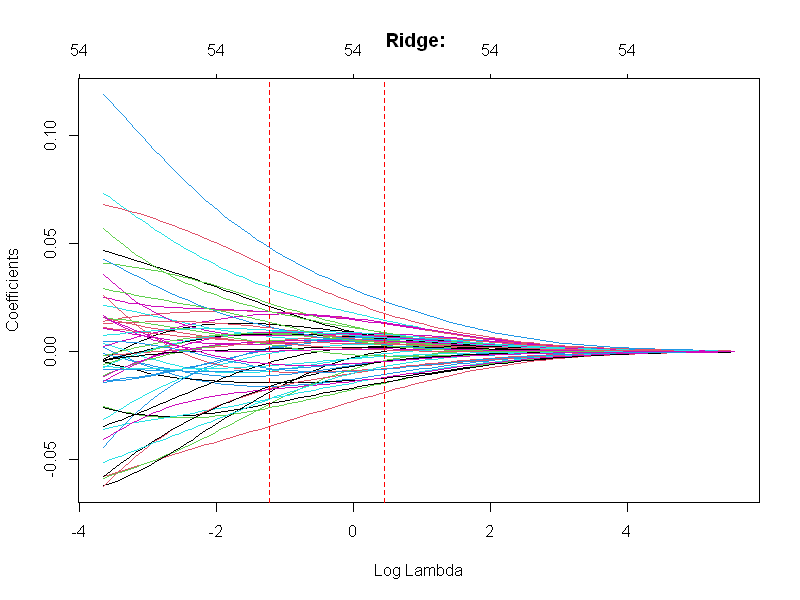


"Max Lambda: 259.875"

"Min Lambda: 0.026"

Minimum MSE for CV: 0.0600

Lambda for Corresponding Min. MSE : 0.2424

Min MSE for 1st Error: 0.0748

Corresponding Lambda for 1 Std Err : 2.4806

Log (Lambda Min. MSE): -1.4173

Log (Lambda 1 Std Error MSE): 0.9085

Test RMSE: 0.2126

Train RMSE: 0.1972

Train R_squared: 0.7075

Test R_squared: 0.7296

Top 10 variables with high coefficients: Literacy, Pharmacy, Marriage, Cancer. Mortality, Graduates. Density, Birth. Rate, Primary. Care, PM2_5, Physician, Fertility. Rate


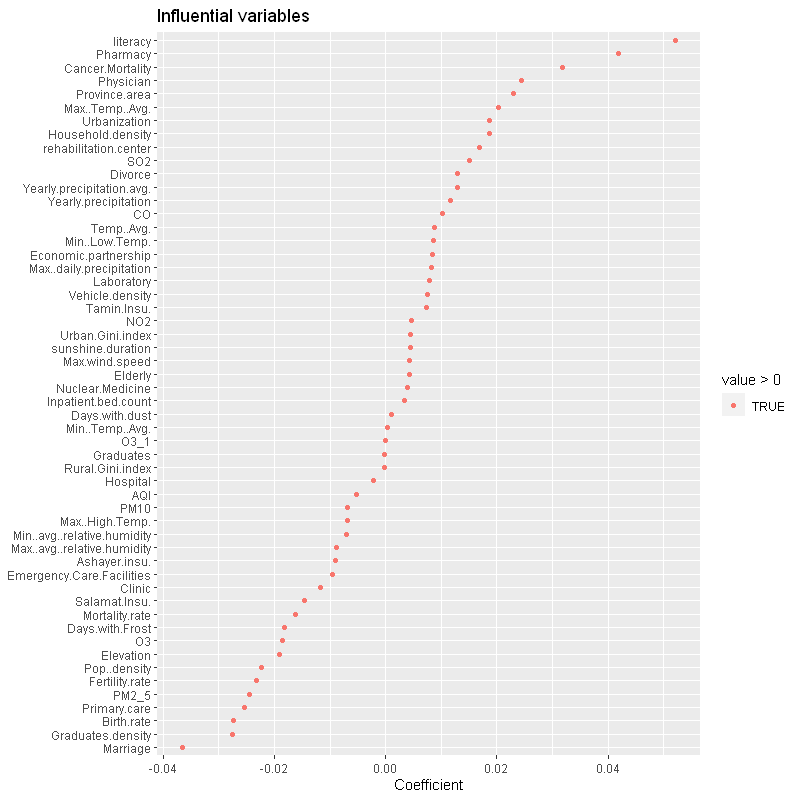


### **A-2:** Explanatory Regression Analysis Results for gender-integrated population


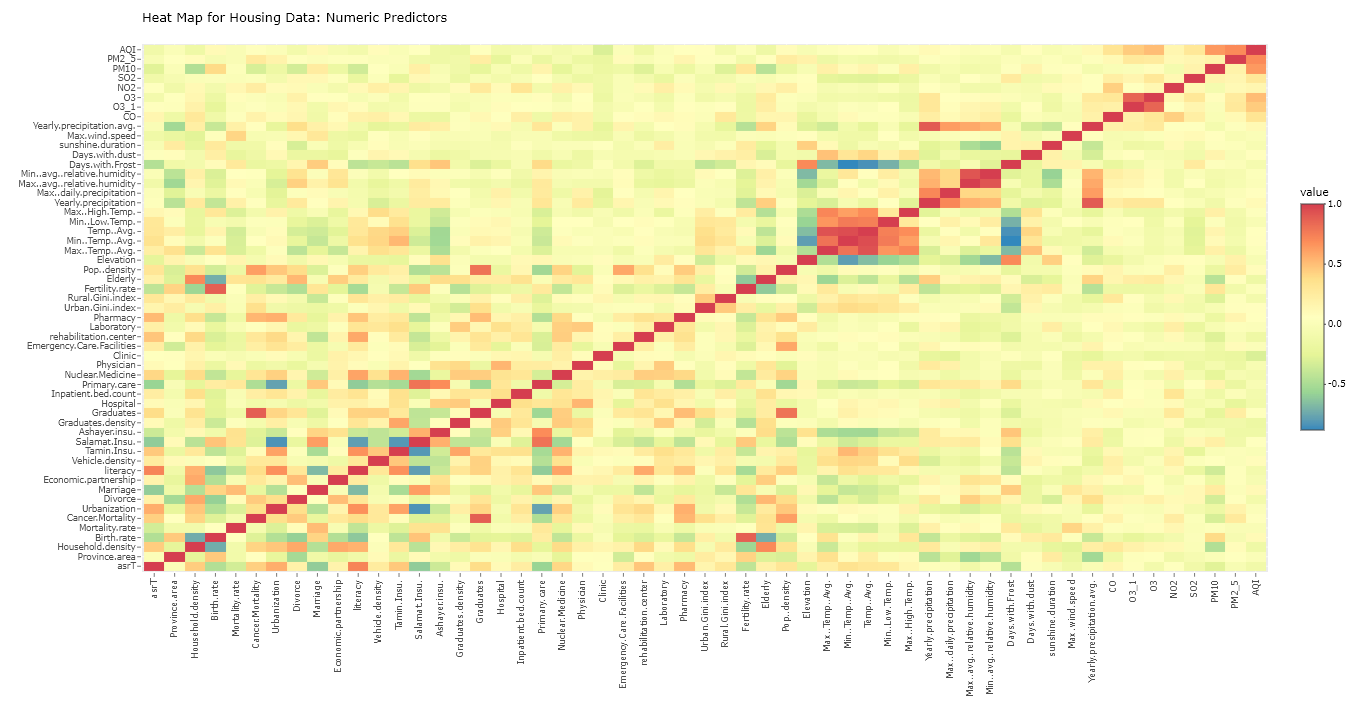


Train RMSE: 2.786787

Test RMSE: 4.385147

Train R-squared: 0.7880308

Test R-squared: 0.5576736

| Variable | coef | tval | pval | VIF |
| --- | --- | --- | --- | --- |
| Mortality. Rate | 0.00114535 | 0.26198296 | 0.75471598 | 3.47210463 |
| Economic. Partnership | -0.04814102 | -0.19308197 | 0.77314949 | 6.30697117 |
| Vehicle. Density | 0.00043097 | 0.59794176 | 0.56811033 | 4.5504663 |
| Graduates. Density | -0.00134357 | -0.62454866 | 0.55923823 | 6.27497384 |
| Inpatient bed count | 0.01075115 | 1.06429504 | 0.31658902 | 2.22036068 |
| Nuclear. Medicine | -0.31214334 | -0.57891792 | 0.59954309 | 4.32193208 |
| Physician | 0.0485918 | 1.1083145 | 0.29565196 | 3.31726827 |
| Clinic | 0.05162158 | 0.4012971 | 0.66381142 | 1.75455843 |
| Emergency Care Facilities | 0.09435794 | 0.35807657 | 0.72943972 | 3.29073553 |
| Rehabilitation. Center | 0.03515496 | 0.27864674 | 0.6868838 | 2.99227606 |
| Laboratory | 0.10470794 | 0.35162634 | 0.60072147 | 5.94290382 |
| Pharmacy | 0.0249058 | 1.50342873 | 0.16475443 | 3.12562167 |
| Urban Gini index | 15.5658776 | 0.8903469 | 0.39447811 | 5.17894008 |
| Rural Gini Index | 2.10188331 | 0.11805744 | 0.78047277 | 3.0808645 |
| Max High Temp. | -0.20810902 | -0.80334803 | 0.44073099 | 7.121608 |
| Max daily precipitation | -0.00041649 | -0.01889178 | 0.75577029 | 4.55389308 |
| Days with dust | -0.00777034 | -0.87596996 | 0.39928255 | 2.77283114 |
| Sunshine duration | 0.00063225 | 0.83260584 | 0.41105754 | 2.51382488 |
| Max wind.speed | 0.036904 | 0.7576823 | 0.50404048 | 2.3233558 |
| CO | -0.0030091 | -0.09790425 | 0.7945002 | 3.06780622 |
| NO2 | -0.00219925 | -0.05317579 | 0.78522405 | 2.25357639 |
| SO2 | 0.03893986 | 1.03707571 | 0.33242373 | 2.26228038 |

### **A-3:** OLS Regression Analysis Results for gender-integrated population

Train RMSE: 4.211989

Test RMSE: 5.053276

Train R-squared: 0.5159018

Test R-squared: 0.3905401

| Variable | coef | tval | pval | VIF |
| --- | --- | --- | --- | --- |
| Mortality rate | -0.01140001 | -2.830817 | 0.0067772778 | 1.639914 |
| rehabilitation center | 0.31812700 | 2.761809 | 0.0200582469 | 1.412047 |
| Pharmacy | 0.05989252 | 4.073533 | 0.0003061824 | 1.438072 |
| Rural Gini index | 44.96837747 | 2.657986 | 0.0122979671 | 1.639059 |

### **A-4:** Stepwise Regression Analysis Results for both genders dataset

Call:

lm (formula = y ~ Cancer. Mortality + Fertility. Rate + Graduates. Density + literacy + Marriage + Pharmacy + Physician + PM2_5 + Primary. Care, data = x)

Residuals:

| Min | 1Q | Median | 3Q | Max |
| --- | --- | --- | --- | --- |
| -8.2827 | -2.1787 | -0.3981 | 1.6136 | 13.3418 |

Coefficients:

|  | Estimate | Std. Error | t-value | Pr (>\|t\|) | Sig. Codes |
| --- | --- | --- | --- | --- | --- |
| (Intercept) | -1.931e+01 | 1.297e+01 | -1.489 | 0.13866 |  |
| Cancer. Mortality | 5.923e-04 | 3.359e-04 | 1.763 | 0.07993 | . |
| Fertility. Rate | -1.433e+00 | 8.232e-01 | -1.741 | 0.08385 | . |
| Graduates. Density | -3.141e-03 | 1.054e-03 | -2.981 | 0.00337 | ** |
| Literacy | 5.457e-01 | 1.303e-01 | 4.189 | 4.85e-05 | *** |
| Marriage | -7.707e-03 | 2.574e-03 | -2.994 | 0.00324 | ** |
| Pharmacy | 2.851e-02 | 1.170e-02 | 2.437 | 0.01602 | * |
| Physician | 7.216e-02 | 2.745e-02 | 2.629 | 0.00948 | ** |
| PM2_5 | -2.587e-02 | 9.219e-03 | -2.806 | 0.00570 | ** |
| Primary. Care | -7.320e-02 | 2.969e-02 | -2.466 | 0.01484 | * |

Significant. Codes: 0 ‘***’ 0.001 ‘**’ 0.01 ‘*’ 0.05 ‘.’ 0.1 ‘ ’ 1

Residual standard error: 3.664 on 145 degrees of freedom

Multiple R-squared: 0.6575, Adjusted R-squared: 0.6363

F-statistic: 30.93 on 9 and 145 DF, p-value: < 2.2e-16

## B: Feature Selection analysis results for Women

###
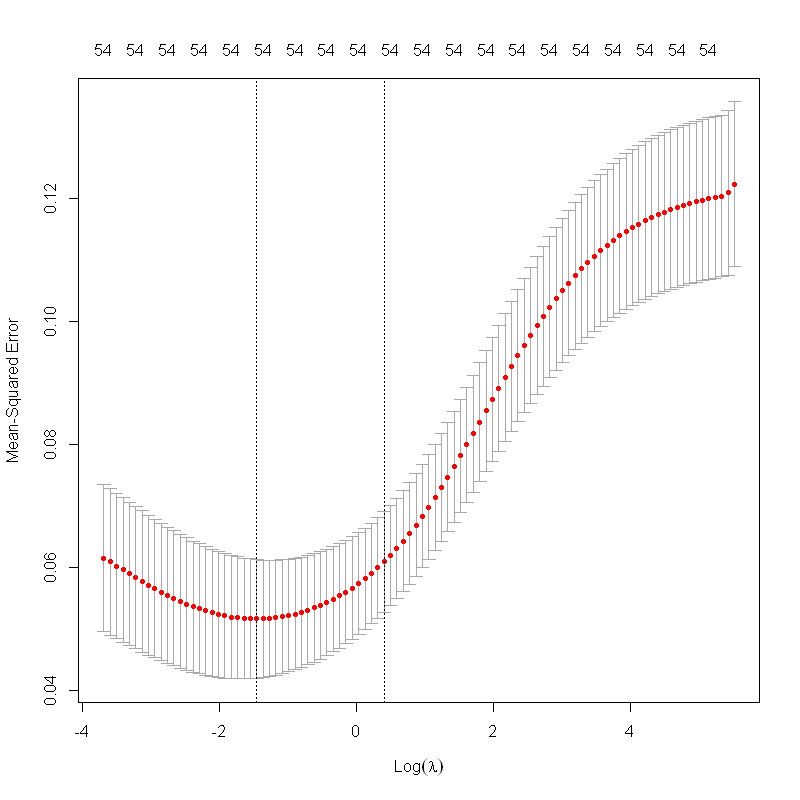
**B-1:** Ridge Regression Analysis Results for Women


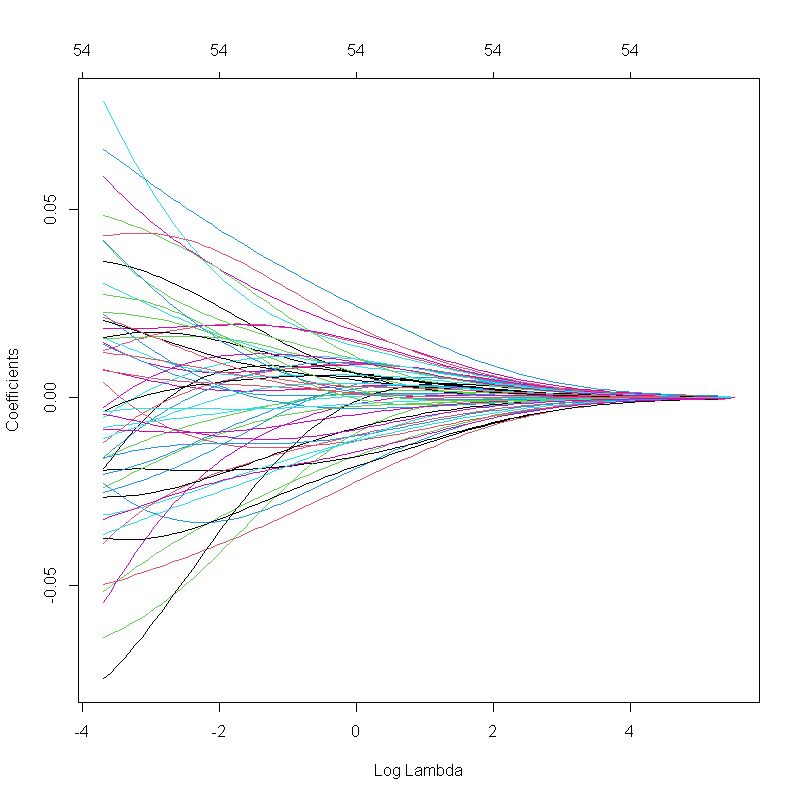


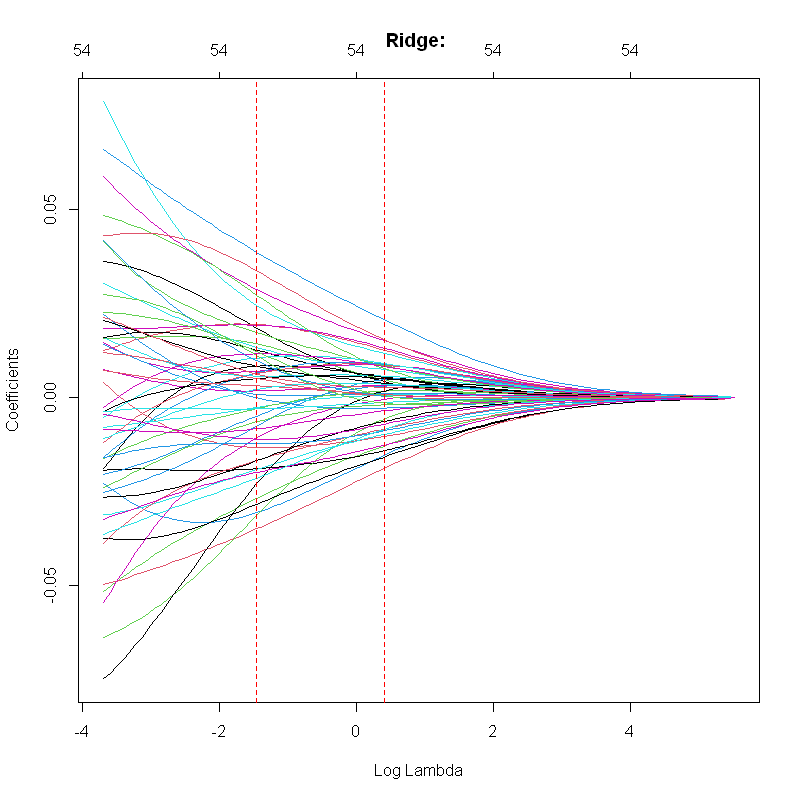


"Max Lambda: 249.913"

"Min Lambda: 0.025"

Minimum MSE for CV: 0.0539

Lambda for Corresponding Min. MSE : 0.2807

Min MSE for 1st Error: 0.0641

Corresponding Lambda for 1 Std Err : 1.8046

Log (Lambda Min. MSE): -1.2704

Log (Lambda 1 Std Error MSE): 0.5903

Test RMSE: 0.2717

Train RMSE: 0.1891

Train R_squared: 0.7212981

Test R_squared: 0.641872

Top 10 variables with high coefficients: literacy, Marriage, Pharmacy, Mortality. Rate, Graduates. Density, Urbanization, Primary. Care, Birth. Rate, Physician, Cancer. Mortality


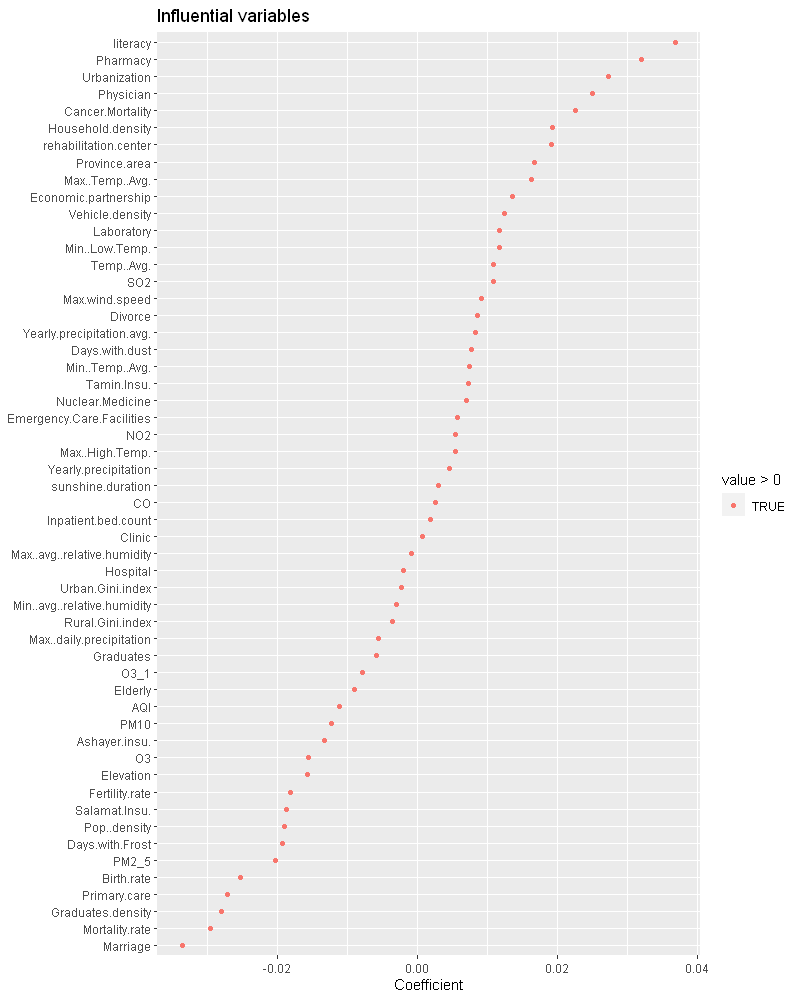


### **B-2:** Explanatory Regression Analysis Results for Women


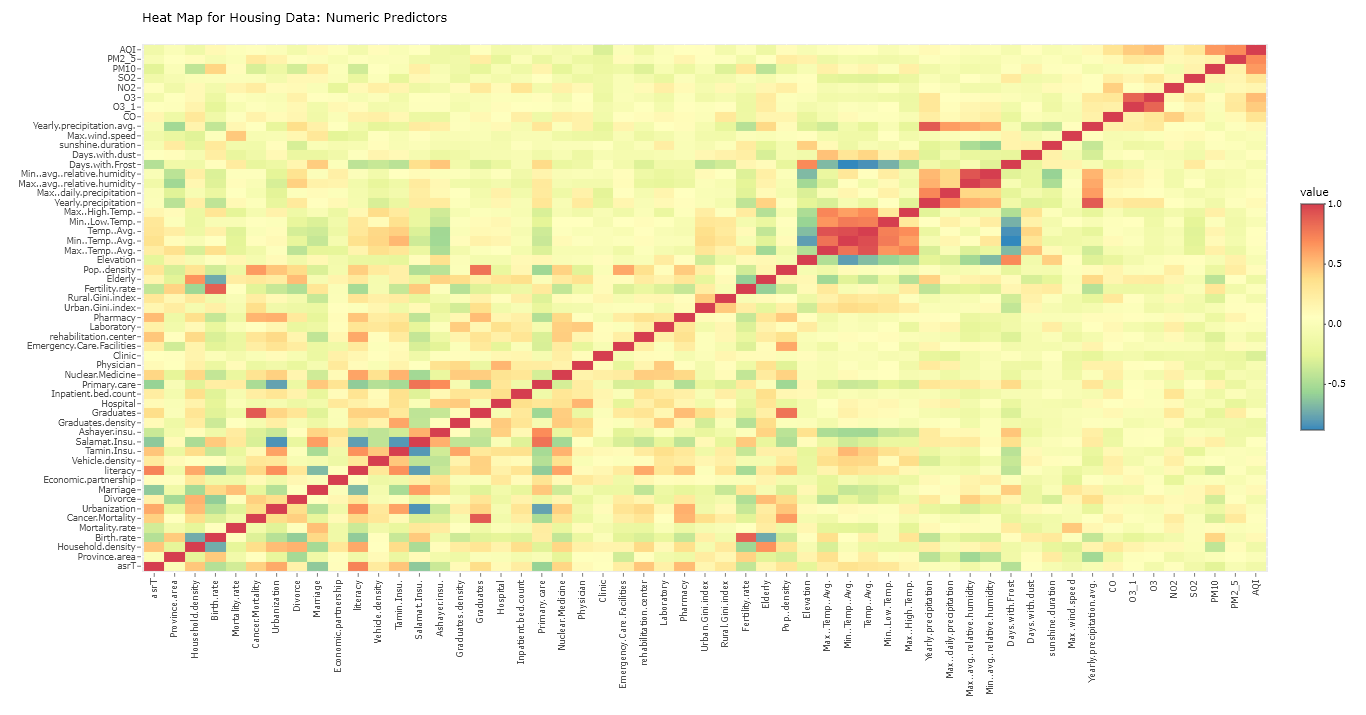


Train RMSE: 5.45154

Test RMSE: 9.450683

Train R-squared: 0.7987081

Test R-squared: 0.4958768

| Variable | coef | tval | pval | VIF |
| --- | --- | --- | --- | --- |
| Mortality Rate | 0.00244529 | 0.40547161 | 0.61491174 | 3.50897778 |
| Economic Partnership | 0.18271765 | 0.51904537 | 0.62377513 | 4.45938828 |
| Vehicle Density | 0.0016778 | 1.16870483 | 0.25699656 | 4.25241794 |
| Graduates Density | -0.00331845 | -0.80724839 | 0.4397203 | 6.30359449 |
| Inpatient bed Count | 0.0202097 | 0.97847097 | 0.36609398 | 2.24447435 |
| Nuclear Medicine | -1.14255627 | -1.06297119 | 0.3392895 | 4.13682063 |
| Physician | 0.11886381 | 1.35402309 | 0.22852843 | 3.28834094 |
| Clinic | 0.20400453 | 0.78893442 | 0.46412234 | 1.68504841 |
| Emergency Care Facilities | 0.33927651 | 0.64791995 | 0.53481792 | 3.13146564 |
| Rehabilitation Center | 0.1795281 | 0.73351853 | 0.44366766 | 2.97752537 |
| Laboratory | -0.14303547 | -0.23924176 | 0.75490885 | 5.38208694 |
| Pharmacy | 0.05168742 | 1.58693097 | 0.14809987 | 3.2045568 |
| Urban Gini index | 16.5280976 | 0.50655926 | 0.60216312 | 4.93319941 |
| Rural Gini index | 8.87377377 | 0.25640678 | 0.78134156 | 3.34379395 |
| Max daily precipitation | -0.01871352 | -0.42520858 | 0.66266634 | 4.05367253 |
| Days with dust | -0.00812493 | -0.48190648 | 0.65246581 | 2.58578247 |
| sunshine duration | 0.00036913 | 0.24732103 | 0.72609789 | 2.28455308 |
| Max wind speed | 0.05585646 | 0.57786303 | 0.59413289 | 2.41409975 |
| CO | 0.04246862 | 0.56381075 | 0.60783163 | 3.16590527 |
| NO2 | -0.00725461 | -0.14832974 | 0.70126415 | 2.49594991 |
| SO2 | 0.05618899 | 0.76224205 | 0.47819584 | 2.28872741 |
| PM10 | 0.02040987 | 0.33414075 | 0.66110341 | 6.92280508 |

### **B-3:** OLS Regression Analysis Results for Women

Train RMSE: 8.471836

Test RMSE: 10.52738

Train R-squared: 0.514597

Test R-squared: 0.3409793

| Variable | coef | tval | pval | VIF |
| --- | --- | --- | --- | --- |
| Mortality rate | -0. -0.01787979 | -3.045927 | 0.0040062582 | 1.659449 |
| rehabilitation center | 0.64006647 | 2.764728 | 0.0376768399 | 1.498718 |
| Pharmacy | 0.12089893 | 4.153612 | 0.0002705194 | 1.443709 |
| Rural Gini index | 80.16589042 | 2.256093 | 0.0392105641 | 2.003855 |

### **B-4:** Stepwise Regression Analysis Results for Women

Call:

Lm (formula = y ~ Graduates. Density + literacy + Marriage + Pharmacy +

Physician + Primary. Care, data = x)

Residuals:

| Min | 1Q | Median | 3Q | Max |
| --- | --- | --- | --- | --- |
| -17.1863 | -4.3503 | -0.8197 | 3.6221 | 28.7082 |

Coefficients:

|  | Estimate | Std. Error | t-value | Pr (>\|t\|) | Signif. codes |
| --- | --- | --- | --- | --- | --- |
| (Intercept) | -61.620876 | 24.143944 | -2.552 | 0.01172 | * |
| Graduates. Density | -0.005029 | 0.001966 | -2.558 | 0.01154 | * |
| literacy | 1.258859 | 0.256135 | 4.915 | 2.33e-06 | *** |
| Marriage | -0.016827 | 0.005253 | -3.203 | 0.00166 | ** |
| Pharmacy | 0.073092 | 0.021857 | 3.344 | 0.00105 | ** |
| Physician | 0.138770 | 0.055761 | 2.489 | 0.01393 | * |
| Primary. Care | -0.147722 | 0.059031 | -2.502 | 0.01342 | * |

---

Signif. Codes: 0 ‘***’ 0.001 ‘**’ 0.01 ‘*’ 0.05 ‘.’ 0.1 ‘ ’ 1

Residual standard error: 7.501 on 148 degrees of freedom

Multiple R-squared: 0.6369, Adjusted R-squared: 0.6222

F-statistic: 43.27 on 6 and 148 DF, p-value: < 2.2e-16

## C: Feature Selection analysis results for Men

### **C-1:**
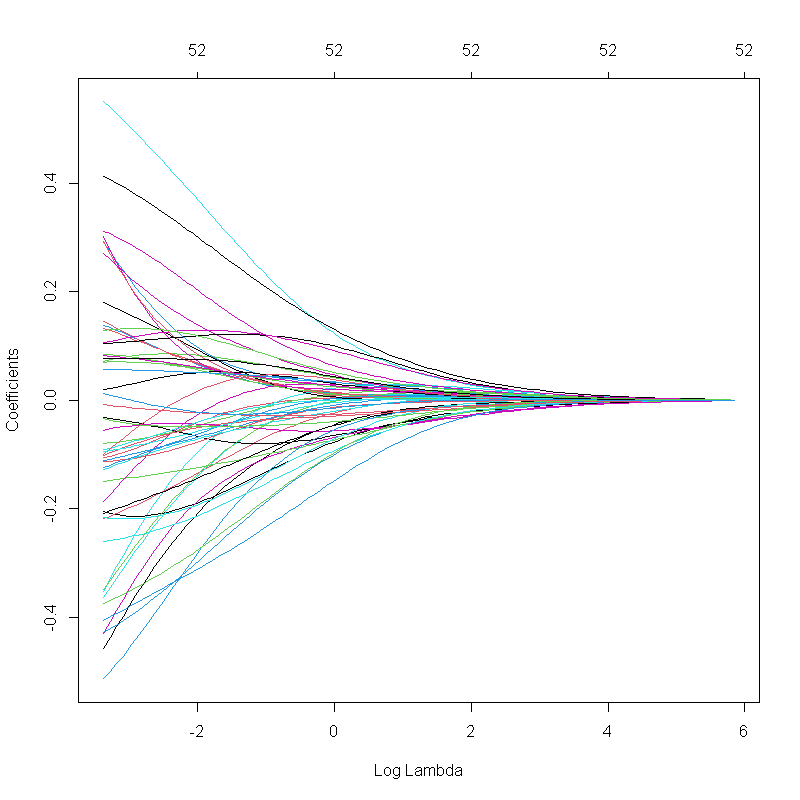

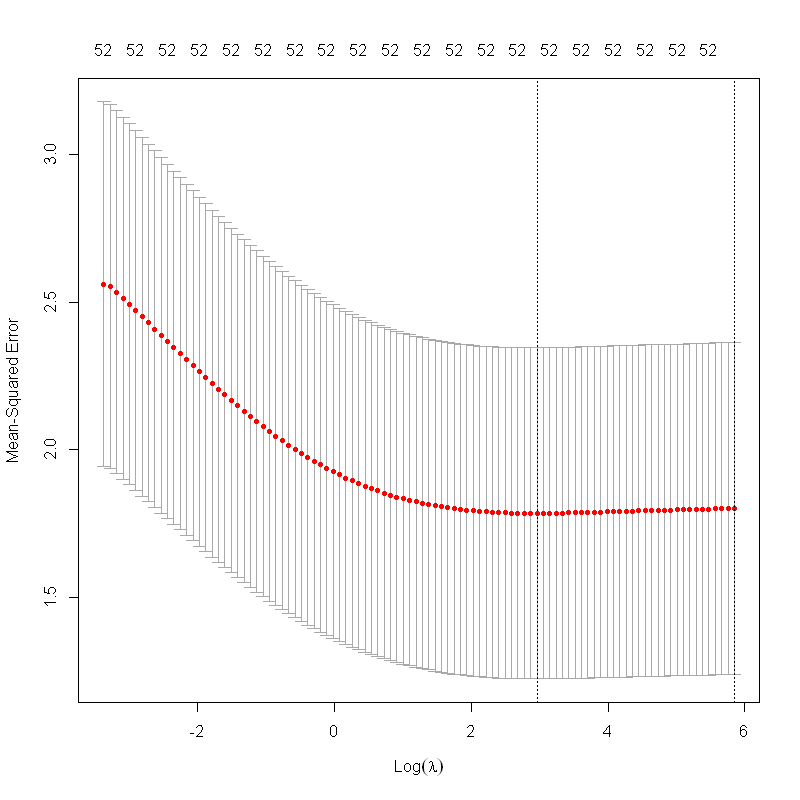
Ridge Regression Analysis Results for Men


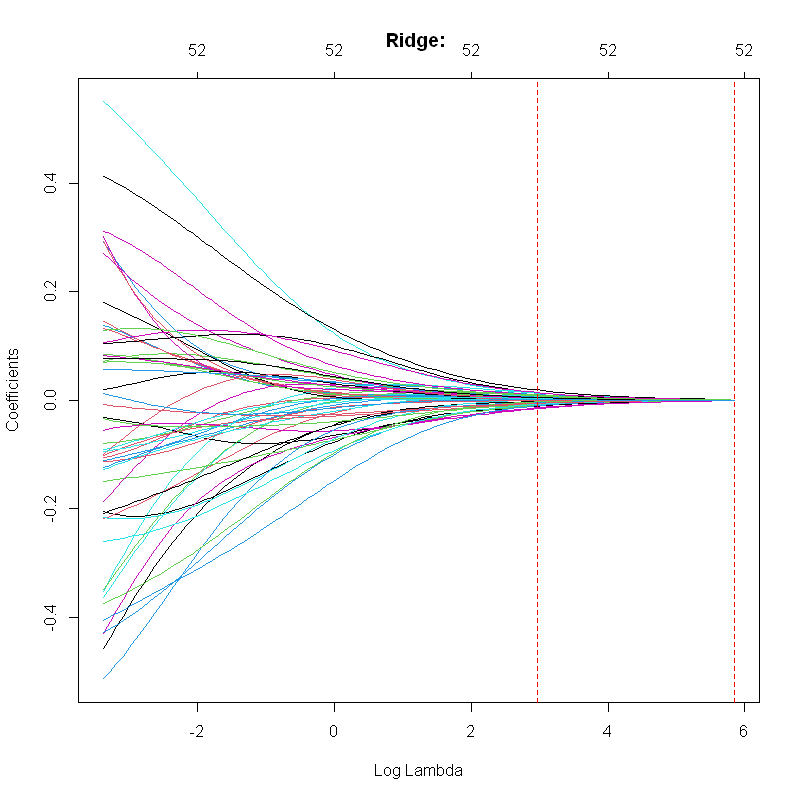


"Max Lambda: 346.010 "

"Min Lambda: 0.035 "

Minimum MSE for CV: 1.7763

Lambda for Corresponding Min. MSE : 10.0864

Min MSE for 1st Error: 1.8148

Corresponding Lambda for 1 Std Err : 346.0096

Log (Lambda Min. MSE): 2.3112

Log (Lambda 1 Std Error MSE): 5.8465

Test RMSE: 0.6965

Train RMSE: 1.2766

Train R_squared: 0.1566

Test R_squared: 0.0004

Top 10 variables with high coefficients: Pharmacy, Max wind speed, Primary. Care, NO2, Inpatient bed. Count, Salamat Insu., Emergency Care. Facilities, Urbanization, Ashayer insu., Clinic


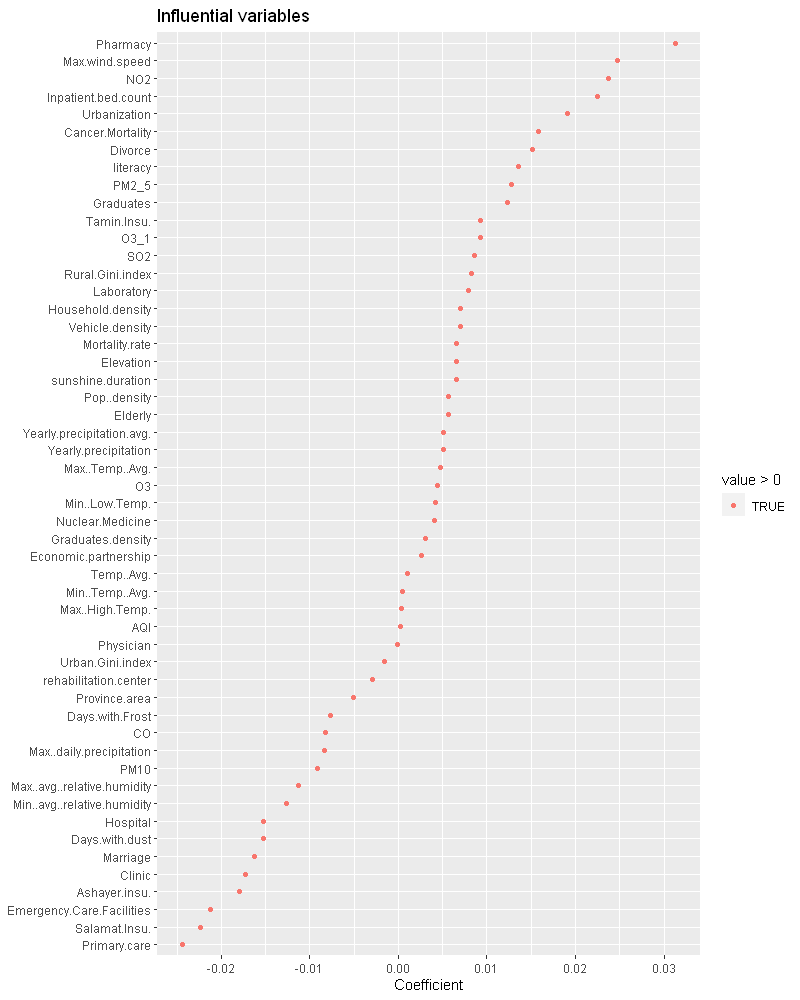


### **C-2:** Explanatory Regression Analysis Results for Men


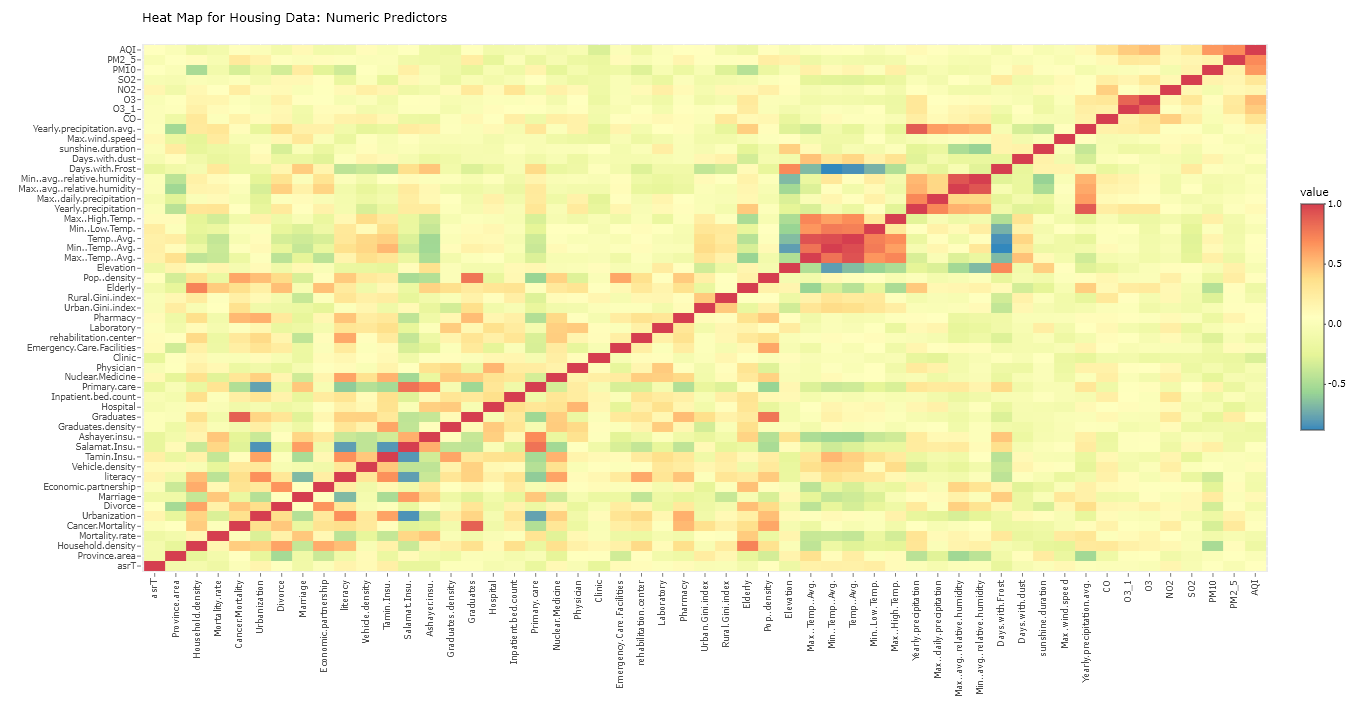


Train RMSE: 0.4433342

Test RMSE: 0.6500229

Train R-squared: 0.3425539

Test R-squared: 0.07067559

| Variable | coef | tval | pval | VIF |
| --- | --- | --- | --- | --- |
| Mortality rate | 0.01977387 | 0.1907675 | 0.73984702 | 4.11212919 |
| Cancer Mortality | -0.10189265 | -0.86043325 | 0.44274133 | 6.02955811 |
| Marriage | -0.18649853 | -1.56782976 | 0.15250326 | 6.59742865 |
| Economic partnership | 0.06395015 | 0.65003463 | 0.54123771 | 4.57128489 |
| Vehicle density | 0.11597233 | 1.28168687 | 0.22387442 | 3.69739461 |
| Graduates density | -0.03142505 | -0.35813558 | 0.68748605 | 3.32197086 |
| Hospital | -0.14287674 | -1.27544462 | 0.21847322 | 5.69846353 |
| Inpatient bed count | -0.01584847 | -0.29049811 | 0.70026781 | 1.89297428 |
| Nuclear Medicine | 0.09557927 | 1.09882609 | 0.32148892 | 3.47717335 |
| Physician | 0.0038379 | 0.02806119 | 0.75710716 | 2.80334584 |
| Clinic | -0.13137925 | -2.24868858 | 0.03305656 | 1.5797818 |
| Emergency Care Facilities | 0.03124807 | 0.46076267 | 0.63266628 | 2.01894649 |
| rehabilitation center | 0.0414251 | 0.59586065 | 0.56674049 | 2.51777678 |
| Laboratory | -0.00393078 | -0.04243854 | 0.89677872 | 5.00040543 |
| Pharmacy | 0.09890011 | 1.2398692 | 0.23721988 | 2.89858337 |
| Urban Gini index | -0.03111211 | -0.33538911 | 0.70779061 | 3.83779651 |
| Rural Gini index | 0.0141016 | 0.18135062 | 0.70641795 | 2.93185349 |
| Pop density | -0.10283273 | -0.92875641 | 0.37588996 | 5.69005794 |
| Min Low Temp | 0.06462839 | 0.73693605 | 0.45445016 | 3.8300549 |
| Max High Temp | -0.09492253 | -0.85857876 | 0.40608036 | 5.88033735 |
| Max daily precipitation | -0.13039982 | -1.48988492 | 0.16616426 | 3.58320945 |
| Days with dust | -0.11301905 | -1.70617554 | 0.09975851 | 2.24172131 |
| sunshine duration | 0.01022129 | -0.1024661 | 0.75621912 | 2.0952179 |
| Max wind speed | 0.01564012 | 0.27279857 | 0.78569232 | 1.83196255 |
| CO | -0.06196829 | -0.85846931 | 0.42365448 | 2.51965183 |
| O3 | 0.00417801 | 0.04690358 | 0.7485973 | 2.29360974 |
| NO2 | 0.10641819 | 1.65196009 | 0.1301614 | 1.81690438 |
| SO2 | -0.0611485 | -0.93281675 | 0.37761934 | 2.08378982 |
| PM10 | -0.00426572 | -0.05591953 | 0.74983359 | 2.83846592 |
| PM2_5 | 0.01638489 | 0.24982072 | 0.72742577 | 2.01932537 |

### **C-3:** OLS Regression Analysis Results for the Men's Dataset

Train RMSE: 0.4620892

Test RMSE: 0.5570395

Train R-squared: 0.2854902

Test R-squared: 0.0856951

| Variable | coef | tval | pvalMean | VIF |
| --- | --- | --- | --- | --- |
| Clinic | -0.047433436 | -2.737122 | 0.009482112 | 1.472785 |
| Min.Low.Temp. | 0.013963864 | 2.047669 | 0.067675571 | 1.834252 |
| Days.with.dust | -0.001999833 | -2.040996 | 0.047760568 | 1.522253 |

### **C-4:** Stepwise Regression Analysis Results for Men

Call:

Lm (formula = y ~ Clinic + Days.with.dust + Min.Low.Temp. + Salamat.Insu., data = x)

Residuals:

| Min | 1Q | Median | 3Q | Max |
| --- | --- | --- | --- | --- |
| -1.0777 | -0.3041 | -0.0584 | 0.2500 | 3.1907 |

Coefficients:

|  | Estimate | Std. Error | T value | Pr(>\|t\|) | Signif. codes |
| --- | --- | --- | --- | --- | --- |
| (Intercept) | 1.6920951 | 0.1880182 | 9.000 | 9.39e-16 | *** |
| Clinic | -0.0435343 | 0.0133599 | -3.259 | 0.00139 | ** |
| Days with dust | -0.0011555 | 0.0007533 | -1.534 | 0.12715 |  |
| Min Low Temp | 0.0126034 | 0.0049635 | 2.539 | 0.01213 | * |
| Salamat Insu | -0.0078235 | 0.0028055 | -2.789 | 0.00598 | ** |

---

Signif. Codes: 0 ‘***’ 0.001 ‘**’ 0.01 ‘*’ 0.05 ‘.’ 0.1 ‘ ’ 1

Residual standard error: 0.5118 on 150 degrees of freedom

Multiple R-squared: 0.1547, Adjusted R-squared: 0.1321

F-statistic: 6.862 on 4 and 150 DF, p-value: 4.239e-05
