# Supplementary material for: Exploring the geospatial epidemiology of breast cancer in Iran: identifying significant risk factors and spatial patterns for evidence-based prevention strategies
Source: BMC Cancer. 2023 Dec 11;23:1219. doi: 10.1186/s12885-023-11555-1 (PMC10712175; doi:10.1186/s12885-023-11555-1)
Supplement: Supplementary file 3 — Additional file 3. [file 12885_2023_11555_MOESM3_ESM.docx]

# Library -----------------------------------------------------------------

library(MASS)

library(stringr)

library(epiR)

library(openxlsx)

library(tidyverse)

library(scales)

library(gridExtra)

library(ggplot2)

library(ggpattern)

library(cowplot)

library(viridis)

library(GGally)

library(glmnet)

library(rsample)

library(reshape2)

library(plotly)

library(caret)

library(ggcorrplot)

library(rsq)

library(car)

library(caret)

library(dplyr)

library(tidyr)

#library(raster)

library(sp)

# Load Data ---------------------------------------------------------------

data<-data.frame(read.xlsx("RF.xlsx",sheet = "PerYear"), stringsAsFactors = F)

#row.names(data)<-paste(data$Province,"-",data$Year,"-",data$Sex)

# Air Clean ---------------------------------------------------------------

Air1<-Air[,c(22,5:13,18,41)]

view(Air1[1:50,])

Air1[is.na(Air1)]<-0

Air1$Year<-str_sub(Air1$Date_Shamsi,1,4)

Air1$Year<-str_replace(Air1$Year,"1393","2014")

Air1$Year<-str_replace(Air1$Year,"1394","2015")

Air1$Year<-str_replace(Air1$Year,"1395","2016")

Air1$Year<-str_replace(Air1$Year,"1396","2017")

Air1$Year<-str_replace(Air1$Year,"1397","2018")

Air1$Year<-str_replace(Air1$Year,"1398","2019")

Air1$Year<-str_replace(Air1$Year,"1399","2020")

Air1$Year<-str_replace(Air1$Year,"1400","2021")

Air1$Year<-str_replace(Air1$Year,"1401","2022")

Air1<-Air1 %>%

group_by(StateName_En,Year) %>%

summarise(CO=round(mean(CO),2),O3_1=round(mean(O3_1),2),O3=round(mean(O3),2),NO2=round(mean(NO2),2),

SO2=round(mean(SO2),2),PM10=round(mean(PM10),2),PM2_5=round(mean(PM2_5),2),AQI=round(mean(AQI),2))

Air1$StateName_En<-str_replace(Air1$StateName_En,"AZ-East","East Azerbaijan")

Air1$StateName_En<-str_replace(Air1$StateName_En,"AZ-West","West Azerbaijan")

Air1$StateName_En<-str_replace(Air1$StateName_En,"Chahar Mahal","Chaharmahal and Bakhtiari")

Air1$StateName_En<-str_replace(Air1$StateName_En,"Ghazvin","Qazvin")

Air1$StateName_En<-str_replace(Air1$StateName_En,"Kerman Shah","Kermanshah")

Air1$StateName_En<-str_replace(Air1$StateName_En,"Khorasan Razavi","Razavi Khorasan")

Air1$StateName_En<-str_replace(Air1$StateName_En,"Khorasan Jonoubi","South Khorasan")

Air1$StateName_En<-str_replace(Air1$StateName_En,"Khorasan Shomali","North Khorasan")

Air1$StateName_En<-str_replace(Air1$StateName_En,"Khouzestan","Khuzestan")

Air1$StateName_En<-str_replace(Air1$StateName_En,"Kohgolie va Boyer Ahman","Kohgiluyeh and Boyer-Ahmad")

Air1$StateName_En<-str_replace(Air1$StateName_En,"Kordestan","Kurdistan")

Air1$StateName_En<-str_replace(Air1$StateName_En,"Markazy","Markazi")

Air1$StateName_En<-str_replace(Air1$StateName_En,"Sistan va blouchestan","Sistan and Baluchestan")

Air1$StateName_En<-str_replace(Air1$StateName_En,"YAZD","Yazd")

Air1$Merg<-paste(Air1$StateName_En,Air1$Year)

# AIrQuality Data ---------------------------------------------------------

Air<-read.csv("Air1.csv",header = TRUE,sep = ",")

data$Merg<-paste(data$Province,data$Year)

data<-merge(data,Air[,4:12],by="Merg",all.x = TRUE)

data$Merg<-NULL

data[is.na(data)]<-0

# Split Data --------------------------------------------------------------

Total<-subset(data,data$Sex=="T")

Women<-subset(data,data$Sex=="F")

Men<-subset(data,data$Sex=="M")

# ASR Total per province---------------------------------------------------------------

Total$per0.4<-(Total$X0.4/Total$X0.4Pop)*100000

Total$per5.9<-(Total$X5.9/Total$X5.9Pop)*100000

Total$per10.14<-(Total$X10.14/Total$X10.14Pop)*100000

Total$per15.19<-(Total$X15.19/Total$X15.19Pop)*100000

Total$per20.24<-(Total$X20.24/Total$X20.24Pop)*100000

Total$per25.29<-(Total$X25.29/Total$X25.29Pop)*100000

Total$per30.34<-(Total$X30.34/Total$X30.34Pop)*100000

Total$per35.39<-(Total$X35.39/Total$X35.39Pop)*100000

Total$per40.44<-(Total$X40.44/Total$X40.44Pop)*100000

Total$per45.49<-(Total$X45.49/Total$X45.49Pop)*100000

Total$per50.54<-(Total$X50.54/Total$X50.54Pop)*100000

Total$per55.59<-(Total$X55.59/Total$X55.59Pop)*100000

Total$per60.64<-(Total$X60.64/Total$X60.64Pop)*100000

Total$per65.69<-(Total$X65.69/Total$X65.69Pop)*100000

Total$per70.74<-(Total$X70.74/Total$X70.74Pop)*100000

Total$per75.79<-(Total$X75.79/Total$X75.79Pop)*100000

Total$per80.84<-(Total$X80.84/Total$X80.84Pop)*100000

Total$per85.89<-(Total$X85.89/Total$X85.89Pop)*100000

Total$per90.94<-(Total$X90.94/Total$X90.94Pop)*100000

Total$per95.99<-(Total$X95.99/Total$X95.99Pop)*100000

Total$per100<-(Total$X100./Total$X100.Pop)*100000

Total$per<-round(as.data.frame(epi.conf(as.matrix(cbind(Total$Number,Total$TotalPop)), ctype = "inc.rate",

method = "exact", design = 1, conf.level = 0.95) * 100000),1)

Total$asr0.4<-(Total$per0.4/100000)*8856.9

Total$asr5.9<-(Total$per5.9/100000)*8687

Total$asr10.14<-(Total$per10.14/100000)*8597

Total$asr15.19<-(Total$per15.19/100000)*8467

Total$asr20.24<-(Total$per20.24/100000)*8217.1

Total$asr25.29<-(Total$per25.29/100000)*7927.2

Total$asr30.34<-(Total$per30.34/100000)*7607.3

Total$asr35.39<-(Total$per35.39/100000)*7147.5

Total$asr40.44<-(Total$per40.44/100000)*6587.7

Total$asr45.49<-(Total$per45.49/100000)*6037.9

Total$asr50.54<-(Total$per50.54/100000)*5368.1

Total$asr55.59<-(Total$per55.59/100000)*4548.4

Total$asr60.64<-(Total$per60.64/100000)*3718.7

Total$asr65.69<-(Total$per65.69/100000)*2959

Total$asr70.74<-(Total$per70.74/100000)*2209.2

Total$asr75.79<-(Total$per75.79/100000)*1519.5

Total$asr80.84<-(Total$per80.84/100000)*909.7

Total$asr85.89<-(Total$per85.89/100000)*439.8

Total$asr90.94<-(Total$per90.94/100000)*150

Total$asr95.99<-(Total$per95.99/100000)*40

Total$asr100<-(Total$per100/100000)*5

Total$asr<-Total$asr0.4+Total$asr5.9+Total$asr10.14+Total$asr15.19+Total$asr20.24+Total$asr25.29+Total$asr30.34+

Total$asr35.39+Total$asr40.44+Total$asr45.49+Total$asr50.54+Total$asr55.59+Total$asr60.64+Total$asr65.69+

Total$asr70.74+Total$asr75.79+Total$asr80.84+Total$asr85.89+Total$asr90.94+Total$asr95.99+Total$asr100

Total$asr<-as.data.frame(round(as.data.frame(epi.conf(as.matrix(cbind(Total$asr,100000)), ctype = "inc.rate", method = "exact", design = 1,

conf.level = 0.95) * 100000),1))

Total$asrT<-Total$asr$est

Total$asrL<-Total$asr$lower

Total$asrU<-Total$asr$upper

Total$asrT[Total$Province=="Golestan" & Total$Year=="2015"]<-17.6

Total$asrT[Total$Province=="Golestan" & Total$Year=="2016"]<-19.4

Total$asrT[Total$Province=="Golestan" & Total$Year=="2018"]<-21.6

Total$asrT[Total$Province=="Qom" & Total$Year=="2014"]<-22.9

Total$asrT[Total$Province=="Qom" & Total$Year=="2015"]<-22.9

Total$asrT[Total$Province=="Kurdistan" & Total$Year=="2018"]<-14.4

# ASR Men per province-----------------------------------------------------------------

Men$per0.4<-(Men$X0.4/Men$X0.4Pop)*100000

Men$per5.9<-(Men$X5.9/Men$X5.9Pop)*100000

Men$per10.14<-(Men$X10.14/Men$X10.14Pop)*100000

Men$per15.19<-(Men$X15.19/Men$X15.19Pop)*100000

Men$per20.24<-(Men$X20.24/Men$X20.24Pop)*100000

Men$per25.29<-(Men$X25.29/Men$X25.29Pop)*100000

Men$per30.34<-(Men$X30.34/Men$X30.34Pop)*100000

Men$per35.39<-(Men$X35.39/Men$X35.39Pop)*100000

Men$per40.44<-(Men$X40.44/Men$X40.44Pop)*100000

Men$per45.49<-(Men$X45.49/Men$X45.49Pop)*100000

Men$per50.54<-(Men$X50.54/Men$X50.54Pop)*100000

Men$per55.59<-(Men$X55.59/Men$X55.59Pop)*100000

Men$per60.64<-(Men$X60.64/Men$X60.64Pop)*100000

Men$per65.69<-(Men$X65.69/Men$X65.69Pop)*100000

Men$per70.74<-(Men$X70.74/Men$X70.74Pop)*100000

Men$per75.79<-(Men$X75.79/Men$X75.79Pop)*100000

Men$per80.84<-(Men$X80.84/Men$X80.84Pop)*100000

Men$per85.89<-(Men$X85.89/Men$X85.89Pop)*100000

Men$per90.94<-(Men$X90.94/Men$X90.94Pop)*100000

Men$per95.99<-(Men$X95.99/Men$X95.99Pop)*100000

Men$per100<-(Men$X100./Men$X100.Pop)*100000

Men$per<-round(as.data.frame(epi.conf(as.matrix(cbind(Men$Number,Men$TotalPop)), ctype = "inc.rate", method = "exact", design = 1,

conf.level = 0.95) * 100000),1)

Men$asr0.4<-(Men$per0.4/100000)*8856.9

Men$asr5.9<-(Men$per5.9/100000)*8687

Men$asr10.14<-(Men$per10.14/100000)*8597

Men$asr15.19<-(Men$per15.19/100000)*8467

Men$asr20.24<-(Men$per20.24/100000)*8217.1

Men$asr25.29<-(Men$per25.29/100000)*7927.2

Men$asr30.34<-(Men$per30.34/100000)*7607.3

Men$asr35.39<-(Men$per35.39/100000)*7147.5

Men$asr40.44<-(Men$per40.44/100000)*6587.7

Men$asr45.49<-(Men$per45.49/100000)*6037.9

Men$asr50.54<-(Men$per50.54/100000)*5368.1

Men$asr55.59<-(Men$per55.59/100000)*4548.4

Men$asr60.64<-(Men$per60.64/100000)*3718.7

Men$asr65.69<-(Men$per65.69/100000)*2959

Men$asr70.74<-(Men$per70.74/100000)*2209.2

Men$asr75.79<-(Men$per75.79/100000)*1519.5

Men$asr80.84<-(Men$per80.84/100000)*909.7

Men$asr85.89<-(Men$per85.89/100000)*439.8

Men$asr90.94<-(Men$per90.94/100000)*150

Men$asr95.99<-(Men$per95.99/100000)*40

Men$asr100<-(Men$per100/100000)*5

Men$asr<-Men$asr0.4+Men$asr5.9+Men$asr10.14+Men$asr15.19+Men$asr20.24+Men$asr25.29+Men$asr30.34+

Men$asr35.39+Men$asr40.44+Men$asr45.49+Men$asr50.54+Men$asr55.59+Men$asr60.64+Men$asr65.69+

Men$asr70.74+Men$asr75.79+Men$asr80.84+Men$asr85.89+Men$asr90.94+Men$asr95.99+Men$asr100

Men$asr<-round(as.data.frame(epi.conf(as.matrix(cbind(Men$asr,100000)), ctype = "inc.rate", method = "exact", design = 1,

conf.level = 0.95) * 100000),1)

Men$asrT<-Men$asr$est

Men$asrL<-Men$asr$lower

Men$asrU<-Men$asr$upper

Men$asrT[Men$Province=="Golestan" & Men$Year=="2016"]<-0.9

Men$asrT[Men$Province=="Golestan" & Men$Year=="2018"]<-0.9

Men$asrT[Men$Province=="Golestan" & Men$Year=="2018"]<-0.9

Men$asrT[Men$Province=="Qom" & Men$Year=="2014"]<-1.1

Men$asrT[Men$Province=="Qom" & Men$Year=="2015"]<-1.1

Men$asrT[Men$Province=="Kurdistan" & Men$Year=="2018"]<-1

Men$asrT[Men$Province=="Chaharmahal and Bakhtiari" & Men$Year=="2018"]<-0.65

# ASR Women per province---------------------------------------------------------------

Women$per0.4<-(Women$X0.4/Women$X0.4Pop)*100000

Women$per5.9<-(Women$X5.9/Women$X5.9Pop)*100000

Women$per10.14<-(Women$X10.14/Women$X10.14Pop)*100000

Women$per15.19<-(Women$X15.19/Women$X15.19Pop)*100000

Women$per20.24<-(Women$X20.24/Women$X20.24Pop)*100000

Women$per25.29<-(Women$X25.29/Women$X25.29Pop)*100000

Women$per30.34<-(Women$X30.34/Women$X30.34Pop)*100000

Women$per35.39<-(Women$X35.39/Women$X35.39Pop)*100000

Women$per40.44<-(Women$X40.44/Women$X40.44Pop)*100000

Women$per45.49<-(Women$X45.49/Women$X45.49Pop)*100000

Women$per50.54<-(Women$X50.54/Women$X50.54Pop)*100000

Women$per55.59<-(Women$X55.59/Women$X55.59Pop)*100000

Women$per60.64<-(Women$X60.64/Women$X60.64Pop)*100000

Women$per65.69<-(Women$X65.69/Women$X65.69Pop)*100000

Women$per70.74<-(Women$X70.74/Women$X70.74Pop)*100000

Women$per75.79<-(Women$X75.79/Women$X75.79Pop)*100000

Women$per80.84<-(Women$X80.84/Women$X80.84Pop)*100000

Women$per85.89<-(Women$X85.89/Women$X85.89Pop)*100000

Women$per90.94<-(Women$X90.94/Women$X90.94Pop)*100000

Women$per95.99<-(Women$X95.99/Women$X95.99Pop)*100000

Women$per100<-(Women$X100./Women$X100.Pop)*100000

Women$per<-round(as.data.frame(epi.conf(as.matrix(cbind(Women$Number,Women$TotalPop)), ctype = "inc.rate", method = "exact", design = 1,

conf.level = 0.95) * 100000),1)

Women$asr0.4<-(Women$per0.4/100000)*8856.9

Women$asr5.9<-(Women$per5.9/100000)*8687

Women$asr10.14<-(Women$per10.14/100000)*8597

Women$asr15.19<-(Women$per15.19/100000)*8467

Women$asr20.24<-(Women$per20.24/100000)*8217.1

Women$asr25.29<-(Women$per25.29/100000)*7927.2

Women$asr30.34<-(Women$per30.34/100000)*7607.3

Women$asr35.39<-(Women$per35.39/100000)*7147.5

Women$asr40.44<-(Women$per40.44/100000)*6587.7

Women$asr45.49<-(Women$per45.49/100000)*6037.9

Women$asr50.54<-(Women$per50.54/100000)*5368.1

Women$asr55.59<-(Women$per55.59/100000)*4548.4

Women$asr60.64<-(Women$per60.64/100000)*3718.7

Women$asr65.69<-(Women$per65.69/100000)*2959

Women$asr70.74<-(Women$per70.74/100000)*2209.2

Women$asr75.79<-(Women$per75.79/100000)*1519.5

Women$asr80.84<-(Women$per80.84/100000)*909.7

Women$asr85.89<-(Women$per85.89/100000)*439.8

Women$asr90.94<-(Women$per90.94/100000)*150

Women$asr95.99<-(Women$per95.99/100000)*40

Women$asr100<-(Women$per100/100000)*5

Women$asr<-Women$asr0.4+Women$asr5.9+Women$asr10.14+Women$asr15.19+Women$asr20.24+Women$asr25.29+Women$asr30.34+

Women$asr35.39+Women$asr40.44+Women$asr45.49+Women$asr50.54+Women$asr55.59+Women$asr60.64+Women$asr65.69+

Women$asr70.74+Women$asr75.79+Women$asr80.84+Women$asr85.89+Women$asr90.94+Women$asr95.99+Women$asr100

Women$asr<-round(as.data.frame(epi.conf(as.matrix(cbind(Women$asr,100000)), ctype = "inc.rate", method = "exact", design = 1,

conf.level = 0.95) * 100000),1)

Women$asrT<-Women$asr$est

Women$asrL<-Women$asr$lower

Women$asrU<-Women$asr$upper

Women$asrT[Women$Province=="Golestan" & Women$Year=="2015"]<-35.76

Women$asrT[Women$Province=="Golestan" & Women$Year=="2016"]<-37.18

Women$asrT[Women$Province=="Golestan" & Women$Year=="2018"]<-40.64

Women$asrT[Women$Province=="Qom" & Women$Year=="2014"]<-45.6

Women$asrT[Women$Province=="Qom" & Women$Year=="2015"]<-45.6

Women$asrT[Women$Province=="Kurdistan" & Women$Year=="2018"]<-36.5

# Iran ASR Total per year-------------------------------------------------------------

dsa<-as.data.frame(Total %>%

group_by(Year) %>%

summarise(X0.4,X5.9,X10.14,X15.19,X20.24,X25.29,X30.34,X35.39,X40.44,X45.49,X50.54,X55.59,X60.64,

X65.69,X70.74,X75.79,X80.84,X85.89,X90.94,X95.99,X100.,X0.4Pop,X5.9Pop,X10.14Pop,X15.19Pop,

X20.24Pop,X25.29Pop,X30.34Pop,X35.39Pop,X40.44Pop,X45.49Pop,X50.54Pop,X55.59Pop,X60.64Pop,

X65.69Pop,X70.74Pop,X75.79Pop,X80.84Pop,X85.89Pop,X90.94Pop,X95.99Pop,X100.Pop))

ASR2014<-dsa[1:31,]

ASR2015<-dsa[32:62,]

ASR2016<-dsa[63:93,]

ASR2017<-dsa[94:124,]

ASR2018<-dsa[125:155,]

ASR2014[32,2:43]<-colSums(ASR2014[,2:43])

ASR2014[32,1]<-"Total2014"

ASR2014<-ASR2014[32,2:43]

ASR2014<-gather(ASR2014,"AgeGroup","Numbers2014")

ASR2014$Pop2014<-ASR2014[22:42,2]

ASR2014<-ASR2014[1:21,]

ASR2015[32,2:43]<-colSums(ASR2015[,2:43])

ASR2015[32,1]<-"Total2015"

ASR2015<-ASR2015[32,2:43]

ASR2015<-gather(ASR2015,"AgeGroup","Numbers2015")

ASR2015$Pop2015<-ASR2015[22:42,2]

ASR2015<-ASR2015[1:21,]

ASR2016[32,2:43]<-colSums(ASR2016[,2:43])

ASR2016[32,1]<-"Total2016"

ASR2016<-ASR2016[32,2:43]

ASR2016<-gather(ASR2016,"AgeGroup","Numbers2016")

ASR2016$Pop2016<-ASR2016[22:42,2]

ASR2016<-ASR2016[1:21,]

ASR2017[32,2:43]<-colSums(ASR2017[,2:43])

ASR2017[32,1]<-"Total2017"

ASR2017<-ASR2017[32,2:43]

ASR2017<-gather(ASR2017,"AgeGroup","Numbers2017")

ASR2017$Pop2017<-ASR2017[22:42,2]

ASR2017<-ASR2017[1:21,]

ASR2018[32,2:43]<-colSums(ASR2018[,2:43])

ASR2018[32,1]<-"Total2018"

ASR2018<-ASR2018[32,2:43]

ASR2018<-gather(ASR2018,"AgeGroup","Numbers2018")

ASR2018$Pop2018<-ASR2018[22:42,2]

ASR2018<-ASR2018[1:21,]

ASRperYear<-cbind(ASR2014,ASR2015[,c(2:3)],ASR2016[,c(2:3)],ASR2017[,c(2:3)],ASR2018[,c(2:3)])

TotalPeryear<-ASRperYear[,c(1,3,5,7,9,11)]

TotalPeryear$MeanPopTotal<-rowMeans(TotalPeryear[,c(2:6)])

TotalPeryear<-TotalPeryear[,c(1,7)]

ASRperYear$per2014<- ASRperYear$Numbers2014/ASRperYear$Pop2014

ASRperYear$per2015<- ASRperYear$Numbers2015/ASRperYear$Pop2015

ASRperYear$per2016<- ASRperYear$Numbers2016/ASRperYear$Pop2016

ASRperYear$per2017<- ASRperYear$Numbers2017/ASRperYear$Pop2017

ASRperYear$per2018<- ASRperYear$Numbers2018/ASRperYear$Pop2018

ASRperYear$WorldPop<-c(8856.9,8687,8597,8467,8217.1,7927.2,7607.3,7147.5,6587.7,6037.9,5368.1,

4548.4,3718.7,2959,2209.2,1519.5,909.7,439.8,150,40,5)

ASRperYear$asr2014<- (ASRperYear$per2014*ASRperYear$WorldPop)

ASRperYear$asr2015<- (ASRperYear$per2015*ASRperYear$WorldPop)

ASRperYear$asr2016<- (ASRperYear$per2016*ASRperYear$WorldPop)

ASRperYear$asr2017<- (ASRperYear$per2017*ASRperYear$WorldPop)

ASRperYear$asr2018<- (ASRperYear$per2018*ASRperYear$WorldPop)

as<-as.matrix(cbind(sum(ASRperYear$asr2014),sum(ASRperYear$WorldPop)))

IranASRPerYear<-data.frame("ASRTot"=round(as.data.frame(epi.conf(as, ctype = "inc.rate", method = "exact", design = 1,

conf.level = 0.95) * 100000),1))

as<-as.matrix(cbind(sum(ASRperYear$asr2015),sum(ASRperYear$WorldPop)))

IranASRPerYear[2,]<-round(as.data.frame(epi.conf(as, ctype = "inc.rate", method = "exact", design = 1,

conf.level = 0.95) * 100000),1)

as<-as.matrix(cbind(sum(ASRperYear$asr2016),sum(ASRperYear$WorldPop)))

IranASRPerYear[3,]<-round(as.data.frame(epi.conf(as, ctype = "inc.rate", method = "exact", design = 1,

conf.level = 0.95) * 100000),1)

as<-as.matrix(cbind(sum(ASRperYear$asr2017),sum(ASRperYear$WorldPop)))

IranASRPerYear[4,]<-round(as.data.frame(epi.conf(as, ctype = "inc.rate", method = "exact", design = 1,

conf.level = 0.95) * 100000),1)

as<-as.matrix(cbind(sum(ASRperYear$asr2018),sum(ASRperYear$WorldPop)))

IranASRPerYear[5,]<-round(as.data.frame(epi.conf(as, ctype = "inc.rate", method = "exact", design = 1,

conf.level = 0.95) * 100000),1)

dsa<-as.data.frame(Women %>%

group_by(Year) %>%

summarise(X0.4,X5.9,X10.14,X15.19,X20.24,X25.29,X30.34,X35.39,X40.44,X45.49,X50.54,X55.59,X60.64,

X65.69,X70.74,X75.79,X80.84,X85.89,X90.94,X95.99,X100.,X0.4Pop,X5.9Pop,X10.14Pop,X15.19Pop,

X20.24Pop,X25.29Pop,X30.34Pop,X35.39Pop,X40.44Pop,X45.49Pop,X50.54Pop,X55.59Pop,X60.64Pop,

X65.69Pop,X70.74Pop,X75.79Pop,X80.84Pop,X85.89Pop,X90.94Pop,X95.99Pop,X100.Pop))

ASR2014<-dsa[1:31,]

ASR2015<-dsa[32:62,]

ASR2016<-dsa[63:93,]

ASR2017<-dsa[94:124,]

ASR2018<-dsa[125:155,]

ASR2014[32,2:43]<-colSums(ASR2014[,2:43])

ASR2014[32,1]<-"Women2014"

ASR2014<-ASR2014[32,2:43]

ASR2014<-gather(ASR2014,"AgeGroup","Numbers2014")

ASR2014$Pop2014<-ASR2014[22:42,2]

ASR2014<-ASR2014[1:21,]

ASR2015[32,2:43]<-colSums(ASR2015[,2:43])

ASR2015[32,1]<-"Women2015"

ASR2015<-ASR2015[32,2:43]

ASR2015<-gather(ASR2015,"AgeGroup","Numbers2015")

ASR2015$Pop2015<-ASR2015[22:42,2]

ASR2015<-ASR2015[1:21,]

ASR2016[32,2:43]<-colSums(ASR2016[,2:43])

ASR2016[32,1]<-"Women2016"

ASR2016<-ASR2016[32,2:43]

ASR2016<-gather(ASR2016,"AgeGroup","Numbers2016")

ASR2016$Pop2016<-ASR2016[22:42,2]

ASR2016<-ASR2016[1:21,]

ASR2017[32,2:43]<-colSums(ASR2017[,2:43])

ASR2017[32,1]<-"Women2017"

ASR2017<-ASR2017[32,2:43]

ASR2017<-gather(ASR2017,"AgeGroup","Numbers2017")

ASR2017$Pop2017<-ASR2017[22:42,2]

ASR2017<-ASR2017[1:21,]

ASR2018[32,2:43]<-colSums(ASR2018[,2:43])

ASR2018[32,1]<-"Women2018"

ASR2018<-ASR2018[32,2:43]

ASR2018<-gather(ASR2018,"AgeGroup","Numbers2018")

ASR2018$Pop2018<-ASR2018[22:42,2]

ASR2018<-ASR2018[1:21,]

ASRperYear<-cbind(ASR2014,ASR2015[,c(2:3)],ASR2016[,c(2:3)],ASR2017[,c(2:3)],ASR2018[,c(2:3)])

WomenPeryear<-ASRperYear[,c(1,3,5,7,9,11)]

WomenPeryear$MeanPopWomen<-rowMeans(WomenPeryear[,c(2:6)])

WomenPeryear<-WomenPeryear[,c(1,7)]

ASRperYear$WorldPop<-c(8856.9,8687,8597,8467,8217.1,7927.2,7607.3,7147.5,6587.7,6037.9,5368.1,

4548.4,3718.7,2959,2209.2,1519.5,909.7,439.8,150,40,5)

ASRperYear$per2014<- ASRperYear$Numbers2014/ASRperYear$Pop2014

ASRperYear$per2015<- ASRperYear$Numbers2015/ASRperYear$Pop2015

ASRperYear$per2016<- ASRperYear$Numbers2016/ASRperYear$Pop2016

ASRperYear$per2017<- ASRperYear$Numbers2017/ASRperYear$Pop2017

ASRperYear$per2018<- ASRperYear$Numbers2018/ASRperYear$Pop2018

ASRperYear$asr2014<- (ASRperYear$per2014*ASRperYear$WorldPop)

ASRperYear$asr2015<- (ASRperYear$per2015*ASRperYear$WorldPop)

ASRperYear$asr2016<- (ASRperYear$per2016*ASRperYear$WorldPop)

ASRperYear$asr2017<- (ASRperYear$per2017*ASRperYear$WorldPop)

ASRperYear$asr2018<- (ASRperYear$per2018*ASRperYear$WorldPop)

as<-as.matrix(cbind(sum(ASRperYear$asr2014),sum(ASRperYear$WorldPop)))

IranASRPerYear[6,]<-data.frame("ASRTot"=round(as.data.frame(epi.conf(as, ctype = "inc.rate", method = "exact", design = 1,

conf.level = 0.95) * 100000),1))

as<-as.matrix(cbind(sum(ASRperYear$asr2015),sum(ASRperYear$WorldPop)))

IranASRPerYear[7,]<-round(as.data.frame(epi.conf(as, ctype = "inc.rate", method = "exact", design = 1,

conf.level = 0.95) * 100000),1)

as<-as.matrix(cbind(sum(ASRperYear$asr2016),sum(ASRperYear$WorldPop)))

IranASRPerYear[8,]<-round(as.data.frame(epi.conf(as, ctype = "inc.rate", method = "exact", design = 1,

conf.level = 0.95) * 100000),1)

as<-as.matrix(cbind(sum(ASRperYear$asr2017),sum(ASRperYear$WorldPop)))

IranASRPerYear[9,]<-round(as.data.frame(epi.conf(as, ctype = "inc.rate", method = "exact", design = 1,

conf.level = 0.95) * 100000),1)

as<-as.matrix(cbind(sum(ASRperYear$asr2018),sum(ASRperYear$WorldPop)))

IranASRPerYear[10,]<-round(as.data.frame(epi.conf(as, ctype = "inc.rate", method = "exact", design = 1,

conf.level = 0.95) * 100000),1)

dsa<-as.data.frame(Men %>%

group_by(Year) %>%

summarise(X0.4,X5.9,X10.14,X15.19,X20.24,X25.29,X30.34,X35.39,X40.44,X45.49,X50.54,X55.59,X60.64,

X65.69,X70.74,X75.79,X80.84,X85.89,X90.94,X95.99,X100.,X0.4Pop,X5.9Pop,X10.14Pop,X15.19Pop,

X20.24Pop,X25.29Pop,X30.34Pop,X35.39Pop,X40.44Pop,X45.49Pop,X50.54Pop,X55.59Pop,X60.64Pop,

X65.69Pop,X70.74Pop,X75.79Pop,X80.84Pop,X85.89Pop,X90.94Pop,X95.99Pop,X100.Pop))

ASR2014<-dsa[1:31,]

ASR2015<-dsa[32:62,]

ASR2016<-dsa[63:93,]

ASR2017<-dsa[94:124,]

ASR2018<-dsa[125:155,]

ASR2014[32,2:43]<-colSums(ASR2014[,2:43])

ASR2014[32,1]<-"Men2014"

ASR2014<-ASR2014[32,2:43]

ASR2014<-gather(ASR2014,"AgeGroup","Numbers2014")

ASR2014$Pop2014<-ASR2014[22:42,2]

ASR2014<-ASR2014[1:21,]

ASR2015[32,2:43]<-colSums(ASR2015[,2:43])

ASR2015[32,1]<-"Men2015"

ASR2015<-ASR2015[32,2:43]

ASR2015<-gather(ASR2015,"AgeGroup","Numbers2015")

ASR2015$Pop2015<-ASR2015[22:42,2]

ASR2015<-ASR2015[1:21,]

ASR2016[32,2:43]<-colSums(ASR2016[,2:43])

ASR2016[32,1]<-"Men2016"

ASR2016<-ASR2016[32,2:43]

ASR2016<-gather(ASR2016,"AgeGroup","Numbers2016")

ASR2016$Pop2016<-ASR2016[22:42,2]

ASR2016<-ASR2016[1:21,]

ASR2017[32,2:43]<-colSums(ASR2017[,2:43])

ASR2017[32,1]<-"Men2017"

ASR2017<-ASR2017[32,2:43]

ASR2017<-gather(ASR2017,"AgeGroup","Numbers2017")

ASR2017$Pop2017<-ASR2017[22:42,2]

ASR2017<-ASR2017[1:21,]

ASR2018[32,2:43]<-colSums(ASR2018[,2:43])

ASR2018[32,1]<-"Men2018"

ASR2018<-ASR2018[32,2:43]

ASR2018<-gather(ASR2018,"AgeGroup","Numbers2018")

ASR2018$Pop2018<-ASR2018[22:42,2]

ASR2018<-ASR2018[1:21,]

ASRperYear<-cbind(ASR2014,ASR2015[,c(2:3)],ASR2016[,c(2:3)],ASR2017[,c(2:3)],ASR2018[,c(2:3)])

MenPeryear<-ASRperYear[,c(1,3,5,7,9,11)]

MenPeryear$MeanPopMen<-rowMeans(MenPeryear[,c(2:6)])

MenPeryear<-MenPeryear[,c(1,7)]

TotalPeryear<-cbind(TotalPeryear,WomenPeryear[,2],MenPeryear[,2])

names(TotalPeryear)<-c("agegroup","PopTotal","PopWomen","PopMen")

ASRperYear$WorldPop<-c(8856.9,8687,8597,8467,8217.1,7927.2,7607.3,7147.5,6587.7,6037.9,5368.1,

4548.4,3718.7,2959,2209.2,1519.5,909.7,439.8,150,40,5)

ASRperYear$per2014<- ASRperYear$Numbers2014/ASRperYear$Pop2014

ASRperYear$per2015<- ASRperYear$Numbers2015/ASRperYear$Pop2015

ASRperYear$per2016<- ASRperYear$Numbers2016/ASRperYear$Pop2016

ASRperYear$per2017<- ASRperYear$Numbers2017/ASRperYear$Pop2017

ASRperYear$per2018<- ASRperYear$Numbers2018/ASRperYear$Pop2018

ASRperYear$asr2014<- (ASRperYear$per2014*ASRperYear$WorldPop)

ASRperYear$asr2015<- (ASRperYear$per2015*ASRperYear$WorldPop)

ASRperYear$asr2016<- (ASRperYear$per2016*ASRperYear$WorldPop)

ASRperYear$asr2017<- (ASRperYear$per2017*ASRperYear$WorldPop)

ASRperYear$asr2018<- (ASRperYear$per2018*ASRperYear$WorldPop)

as<-as.matrix(cbind(sum(ASRperYear$asr2014),sum(ASRperYear$WorldPop)))

IranASRPerYear[11,]<-data.frame("ASRTot"=round(as.data.frame(epi.conf(as, ctype = "inc.rate", method = "exact", design = 1,

conf.level = 0.95) * 100000),1))

as<-as.matrix(cbind(sum(ASRperYear$asr2015),sum(ASRperYear$WorldPop)))

IranASRPerYear[12,]<-round(as.data.frame(epi.conf(as, ctype = "inc.rate", method = "exact", design = 1,

conf.level = 0.95) * 100000),1)

as<-as.matrix(cbind(sum(ASRperYear$asr2016),sum(ASRperYear$WorldPop)))

IranASRPerYear[13,]<-round(as.data.frame(epi.conf(as, ctype = "inc.rate", method = "exact", design = 1,

conf.level = 0.95) * 100000),1)

as<-as.matrix(cbind(sum(ASRperYear$asr2017),sum(ASRperYear$WorldPop)))

IranASRPerYear[14,]<-round(as.data.frame(epi.conf(as, ctype = "inc.rate", method = "exact", design = 1,

conf.level = 0.95) * 100000),1)

as<-as.matrix(cbind(sum(ASRperYear$asr2018),sum(ASRperYear$WorldPop)))

IranASRPerYear[15,]<-round(as.data.frame(epi.conf(as, ctype = "inc.rate", method = "exact", design = 1,

conf.level = 0.95) * 100000),1)

IranASRPerYear$Year<-c("2014","2015","2016","2017","2018","2014","2015","2016","2017","2018","2014","2015","2016","2017","2018")

IranASRPerYear$Sex<-c(rep("T",5),rep("F",5),rep("M",5))

remove(ASR2014,ASR2015,ASR2016,ASR2017,ASR2018,as,ASRperYear,dsa,WomenPeryear,MenPeryear)

# Age groups --------------------------------------------------------------

agegroup<-data.frame(Agegroup=c("0-4","05-09","10-14","15-19","20-24","25-29","30-34","35-39","40-44","45-49","50-54",

"55-59","60-64","65-69","70-74","75-79","80-84","85-89","90-94","95-99","100+"),

Total=c(sum(Total$X0.4),sum(Total$X5.9),sum(Total$X10.14),sum(Total$X15.19),sum(Total$X20.24),

sum(Total$X25.29),sum(Total$X30.34),sum(Total$X35.39),sum(Total$X40.44),sum(Total$X45.49),

sum(Total$X50.54),sum(Total$X55.59),sum(Total$X60.64),sum(Total$X65.69),sum(Total$X70.74),

sum(Total$X75.79),sum(Total$X80.84),sum(Total$X85.89),sum(Total$X90.94),sum(Total$X95.99),

sum(Total$X100.)),

Women=c(sum(Women$X0.4),sum(Women$X5.9),sum(Women$X10.14),sum(Women$X15.19),sum(Women$X20.24),

sum(Women$X25.29),sum(Women$X30.34),sum(Women$X35.39),sum(Women$X40.44),sum(Women$X45.49),

sum(Women$X50.54),sum(Women$X55.59),sum(Women$X60.64),sum(Women$X65.69),sum(Women$X70.74),

sum(Women$X75.79),sum(Women$X80.84),sum(Women$X85.89),sum(Women$X90.94),sum(Women$X95.99),

sum(Women$X100.)),

Men=c(sum(Men$X0.4),sum(Men$X5.9),sum(Men$X10.14),sum(Men$X15.19),sum(Men$X20.24),

sum(Men$X25.29),sum(Men$X30.34),sum(Men$X35.39),sum(Men$X40.44),sum(Men$X45.49),

sum(Men$X50.54),sum(Men$X55.59),sum(Men$X60.64),sum(Men$X65.69),sum(Men$X70.74),

sum(Men$X75.79),sum(Men$X80.84),sum(Men$X85.89),sum(Men$X90.94),sum(Men$X95.99),

sum(Men$X100.)))

agegroup<-cbind(agegroup,TotalPeryear[,c(2:4)])

ds<-as.matrix(agegroup[,c("Total","PopTotal")])

agegroup$PerT<-round(as.data.frame(epi.conf(ds, ctype = "inc.rate", method = "exact", design = 1,

conf.level = 0.95) * 100000),1)

ds<-as.matrix(agegroup[,c("Women","PopWomen")])

agegroup$perF<-round(as.data.frame(epi.conf(ds, ctype = "inc.rate", method = "exact", design = 1,

conf.level = 0.95) * 100000),1)

ds<-as.matrix(agegroup[,c("Men","PopMen")])

agegroup$perM<-round(as.data.frame(epi.conf(ds, ctype = "inc.rate", method = "exact", design = 1,

conf.level = 0.95) * 100000),1)

# ASRtPlot ----------------------------------------------------------------

MeanASRTPerProv<-Total %>%

group_by(Province) %>%

summarise(asrt=mean(asrT))

names(MeanASRTPerProv)<-c("Province","asr")

MeanASRTPerProv$id<-seq(1:31)

label_MeanASRTPerProv <- MeanASRTPerProv

number_of_bar <- nrow(label_MeanASRTPerProv)

angle <- 90 - 360 * (label_MeanASRTPerProv$id-0.5) /number_of_bar

label_MeanASRTPerProv$hjust<-ifelse( angle < -90, 1, 0)

label_MeanASRTPerProv$angle<-ifelse(angle < -90, angle+180, angle)

ASRtPlot <- ggplot(MeanASRTPerProv, aes(x=as.factor(id), y=asr)) +

geom_bar(stat="identity", fill=factor(ifelse(MeanASRTPerProv$Province=="Isfahan","red",ifelse(MeanASRTPerProv$Province=="Sistan and Baluchestan","green","skyblue")))) +

ylim(-10,55) +

theme_minimal() +

theme(axis.text = element_blank(),

axis.title = element_blank(),

panel.grid = element_blank(),

plot.margin = unit(rep(-1,14), "cm")) +

coord_polar(start = 0) +

geom_text(data=label_MeanASRTPerProv, aes(x=id, y=asr+1, label=Province, hjust=hjust),color="black", fontface="bold",alpha=0.6, size=4, angle= label_MeanASRTPerProv$angle, inherit.aes = FALSE) +

geom_text(data=label_MeanASRTPerProv, aes(x=id, y=asr-8, label=asr, hjust=hjust),color="black", fontface="bold",alpha=0.6, size=4, angle= label_MeanASRTPerProv$angle, inherit.aes = FALSE)

MeanASRFPerProv<-Women %>%

group_by(Province) %>%

summarise(mean(asrT))

names(MeanASRFPerProv)<-c("Province","asr")

MeanASRFPerProv$id<-seq(1:31)

label_MeanASRFPerProv <- MeanASRFPerProv

number_of_bar <- nrow(label_MeanASRFPerProv)

angle <- 90 - 360 * (label_MeanASRFPerProv$id-0.5) /number_of_bar

label_MeanASRFPerProv$hjust<-ifelse( angle < -90, 1, 0)

label_MeanASRFPerProv$angle<-ifelse(angle < -90, angle+180, angle)

ASRfPlot <- ggplot(MeanASRFPerProv, aes(x=as.factor(id), y=asr)) +

geom_bar(stat="identity", fill=factor(ifelse(MeanASRFPerProv$Province=="Isfahan","red",ifelse(MeanASRFPerProv$Province=="Sistan and Baluchestan","green","skyblue")))) +

ylim(-10,55) +

theme_minimal() +

theme(axis.text = element_blank(),

axis.title = element_blank(),

panel.grid = element_blank(),

plot.margin = unit(rep(-1,4), "cm")) +

coord_polar(start = 0) +

geom_text(data=label_MeanASRFPerProv, aes(x=id, y=asr+1, label=Province, hjust=hjust),color="black", fontface="bold",alpha=0.6, size=4, angle= label_MeanASRFPerProv$angle, inherit.aes = FALSE) +

geom_text(data=label_MeanASRFPerProv, aes(x=id, y=asr-8, label=asr, hjust=hjust),color="black", fontface="bold",alpha=0.6, size=4, angle= label_MeanASRFPerProv$angle, inherit.aes = FALSE)

MeanASRMPerProv<-Men %>%

group_by(Province) %>%

summarise(mean(asrT))

names(MeanASRMPerProv)<-c("Province","asr")

MeanASRMPerProv$id<-seq(1:31)

label_MeanASRMPerProv <- MeanASRMPerProv

number_of_bar <- nrow(label_MeanASRMPerProv)

angle <- 90 - 360 * (label_MeanASRMPerProv$id-0.5) /number_of_bar

label_MeanASRMPerProv$hjust<-ifelse( angle < -90, 1, 0)

label_MeanASRMPerProv$angle<-ifelse(angle < -90, angle+180, angle)

ASRmPlot <- ggplot(MeanASRMPerProv, aes(x=as.factor(id), y=asr)) +

geom_bar(stat="identity", fill=factor(ifelse(MeanASRMPerProv$Province=="Bushehr","red",ifelse(MeanASRMPerProv$Province=="Ardabil","green","skyblue")))) +

ylim(-0.7,3) +

theme_minimal() +

theme(axis.text = element_blank(),

axis.title = element_blank(),

panel.grid = element_blank(),

plot.margin = unit(rep(-1,14), "cm")) +

coord_polar(start = 0) +

geom_text(data=label_MeanASRMPerProv, aes(x=id, y=asr+0.2, label=Province, hjust=hjust),color="black", fontface="bold",alpha=0.6, size=4, angle= label_MeanASRMPerProv$angle, inherit.aes = FALSE) +

geom_text(data=label_MeanASRMPerProv, aes(x=id, y=asr-0.5, label=asr, hjust=hjust),color="black", fontface="bold",alpha=0.6, size=4, angle= label_MeanASRMPerProv$angle, inherit.aes = FALSE)

ggsave("ASRtPlot.tiff", ASRtPlot, width=12, height=8, dpi=300)

ggsave("ASRfPlot.tiff", ASRfPlot, width=12, height=8, dpi=300)

ggsave("ASRmPlot.tiff", ASRmPlot, width=12, height=8, dpi=300)

MeanAsrPerProv<-cbind(MeanASRTPerProv[,1:2],MeanASRFPerProv[,2],MeanASRMPerProv[,2])

names(MeanAsrPerProv)<-c("Province","MeanTotalASR","MeanWomenASR","MeanMenASR")

remove(angle,number_of_bar,label_MeanASRFPerProv,label_MeanASRMPerProv,

label_MeanASRTPerProv,ds,MeanASRFPerProv,MeanASRMPerProv,MeanASRTPerProv)

# 5-years ASR Iran ----------------------------------------------------------------

Iran<-agegroup

Iran$WorldPop<-c(8856.9,8687,8597,8467,8217.1,7927.2,7607.3,7147.5,6587.7,6037.9,5368.1,

4548.4,3718.7,2959,2209.2,1519.5,909.7,439.8,150,40,5)

Iran$asrT<-((Iran$PerT$est/100000)*Iran$WorldPop)

Iran$asrF<-((Iran$perF$est/100000)*Iran$WorldPop)

Iran$asrM<-((Iran$perM$est/100000)*Iran$WorldPop)

ds<-as.matrix(cbind(sum(Iran$asrT),sum(Iran$WorldPop)))

IranASR<-data.frame("ASRTot"=round(as.data.frame(epi.conf(ds, ctype = "inc.rate", method = "exact", design = 1,

conf.level = 0.95) * 100000),1))

ds<-as.matrix(cbind(sum(Iran$asrF),sum(Iran$WorldPop)))

IranASR[2,]<-round(as.data.frame(epi.conf(ds, ctype = "inc.rate", method = "exact", design = 1,

conf.level = 0.95) * 100000),1)

ds<-as.matrix(cbind(sum(Iran$asrM),sum(Iran$WorldPop)))

IranASR[3,]<-round(as.data.frame(epi.conf(ds, ctype = "inc.rate", method = "exact", design = 1,

conf.level = 0.95) * 100000),1)

IranASR$Names=c("Tot","Women","Men")

remove(Iran,ds)

# Per Year ASR ------------------------------------------------------------

PerYear<- Total %>%

group_by(Province, Year) %>%

summarise(asrT)

PerYears<-subset(PerYear,Year=="2014",select =c(Province,asrT))

PerYears[,3]<-subset(PerYear,Year=="2015",select =asrT)

PerYears[,4]<-subset(PerYear,Year=="2016",select =asrT)

PerYears[,5]<-subset(PerYear,Year=="2017",select =asrT)

PerYears[,6]<-subset(PerYear,Year=="2018",select =asrT)

PerYear<- Women %>%

group_by(Province, Year) %>%

summarise(asrT)

PerYears[,7]<-subset(PerYear,Year=="2014",select =c(asrT))

PerYears[,8]<-subset(PerYear,Year=="2015",select =asrT)

PerYears[,9]<-subset(PerYear,Year=="2016",select =asrT)

PerYears[,10]<-subset(PerYear,Year=="2017",select =asrT)

PerYears[,11]<-subset(PerYear,Year=="2018",select =asrT)

PerYear<- Men %>%

group_by(Province, Year) %>%

summarise(asrT)

PerYears[,12]<-subset(PerYear,Year=="2014",select =asrT)

PerYears[,13]<-subset(PerYear,Year=="2015",select =asrT)

PerYears[,14]<-subset(PerYear,Year=="2016",select =asrT)

PerYears[,15]<-subset(PerYear,Year=="2017",select =asrT)

PerYears[,16]<-subset(PerYear,Year=="2018",select =asrT)

names(PerYears)<-c("Province","2014T","2015T","2016T","2017T","2018T",

"2014F","2015F","2016F","2017F","2018F",

"2014M","2015M","2016M","2017M","2018M")

remove(PerYear)

# 5Years Total ASR Iran ---------------------------------------------------

Tot5Years<-as.data.frame(Total %>%

group_by(Province,Year) %>%

summarise(Number,TotalPop,X0.4,X5.9,X10.14,X15.19,X20.24,X25.29,X30.34,X35.39,X40.44,X45.49,X50.54,X55.59,X60.64,

X65.69,X70.74,X75.79,X80.84,X85.89,X90.94,X95.99,X100.,X0.4Pop,X5.9Pop,X10.14Pop,X15.19Pop,

X20.24Pop,X25.29Pop,X30.34Pop,X35.39Pop,X40.44Pop,X45.49Pop,X50.54Pop,X55.59Pop,X60.64Pop,

X65.69Pop,X70.74Pop,X75.79Pop,X80.84Pop,X85.89Pop,X90.94Pop,X95.99Pop,X100.Pop))

Tot5Years<-Tot5Years %>%

group_by(Province) %>%

summarise(Number=sum(Number), TotalPop=mean(TotalPop),

X0.4=sum(X0.4),X5.9=sum(X5.9),X10.14=sum(X10.14),X15.19=sum(X15.19),X20.24=sum(X20.24),

X25.29=sum(X25.29),X30.34=sum(X30.34),X35.39=sum(X35.39),X40.44=sum(X40.44),X45.49=sum(X45.49),

X50.54=sum(X50.54),X55.59=sum(X55.59),X60.64=sum(X60.64), X65.69=sum( X65.69),X70.74=sum(X70.74),

X75.79=sum(X75.79),X80.84=sum(X80.84),X85.89=sum(X85.89),X90.94=sum(X90.94),X95.99=sum(X95.99),

X100.=sum(X100.),

X0.4Pop=mean(X0.4Pop),X5.9Pop=mean(X5.9Pop),X10.14Pop=mean(X10.14Pop),X15.19Pop=mean(X15.19Pop), X20.24Pop=mean(X20.24Pop),X25.29Pop=mean(X25.29Pop),X30.34Pop=mean(X30.34Pop),X35.39Pop=mean(X35.39Pop), X40.44Pop=mean(X40.44Pop),X45.49Pop=mean(X45.49Pop),X50.54Pop=mean(X50.54Pop),X55.59Pop=mean(X55.59Pop), X60.64Pop=mean(X60.64Pop),X65.69Pop=mean(X65.69Pop),X70.74Pop=mean(X70.74Pop),X75.79Pop=mean(X75.79Pop), X80.84Pop=mean(X80.84Pop),X85.89Pop=mean(X85.89Pop),X90.94Pop=mean(X90.94Pop),X95.99Pop=mean(X95.99Pop),

X100.Pop=mean(X100.Pop))

Tot5Years$per0.4<-(Tot5Years$X0.4/Tot5Years$X0.4Pop)*100000

Tot5Years$per5.9<-(Tot5Years$X5.9/Tot5Years$X5.9Pop)*100000

Tot5Years$per10.14<-(Tot5Years$X10.14/Tot5Years$X10.14Pop)*100000

Tot5Years$per15.19<-(Tot5Years$X15.19/Tot5Years$X15.19Pop)*100000

Tot5Years$per20.24<-(Tot5Years$X20.24/Tot5Years$X20.24Pop)*100000

Tot5Years$per25.29<-(Tot5Years$X25.29/Tot5Years$X25.29Pop)*100000

Tot5Years$per30.34<-(Tot5Years$X30.34/Tot5Years$X30.34Pop)*100000

Tot5Years$per35.39<-(Tot5Years$X35.39/Tot5Years$X35.39Pop)*100000

Tot5Years$per40.44<-(Tot5Years$X40.44/Tot5Years$X40.44Pop)*100000

Tot5Years$per45.49<-(Tot5Years$X45.49/Tot5Years$X45.49Pop)*100000

Tot5Years$per50.54<-(Tot5Years$X50.54/Tot5Years$X50.54Pop)*100000

Tot5Years$per55.59<-(Tot5Years$X55.59/Tot5Years$X55.59Pop)*100000

Tot5Years$per60.64<-(Tot5Years$X60.64/Tot5Years$X60.64Pop)*100000

Tot5Years$per65.69<-(Tot5Years$X65.69/Tot5Years$X65.69Pop)*100000

Tot5Years$per70.74<-(Tot5Years$X70.74/Tot5Years$X70.74Pop)*100000

Tot5Years$per75.79<-(Tot5Years$X75.79/Tot5Years$X75.79Pop)*100000

Tot5Years$per80.84<-(Tot5Years$X80.84/Tot5Years$X80.84Pop)*100000

Tot5Years$per85.89<-(Tot5Years$X85.89/Tot5Years$X85.89Pop)*100000

Tot5Years$per90.94<-(Tot5Years$X90.94/Tot5Years$X90.94Pop)*100000

Tot5Years$per95.99<-(Tot5Years$X95.99/Tot5Years$X95.99Pop)*100000

Tot5Years$per100<-(Tot5Years$X100./Tot5Years$X100.Pop)*100000

Tot5Years$per<-round(as.data.frame(epi.conf(as.matrix(cbind(Tot5Years$Number,Tot5Years$TotalPop)), ctype = "inc.rate",

method = "exact", design = 1, conf.level = 0.95) * 100000),1)

Tot5Years$asr0.4<-(Tot5Years$per0.4/100000)*8856.9

Tot5Years$asr5.9<-(Tot5Years$per5.9/100000)*8687

Tot5Years$asr10.14<-(Tot5Years$per10.14/100000)*8597

Tot5Years$asr15.19<-(Tot5Years$per15.19/100000)*8467

Tot5Years$asr20.24<-(Tot5Years$per20.24/100000)*8217.1

Tot5Years$asr25.29<-(Tot5Years$per25.29/100000)*7927.2

Tot5Years$asr30.34<-(Tot5Years$per30.34/100000)*7607.3

Tot5Years$asr35.39<-(Tot5Years$per35.39/100000)*7147.5

Tot5Years$asr40.44<-(Tot5Years$per40.44/100000)*6587.7

Tot5Years$asr45.49<-(Tot5Years$per45.49/100000)*6037.9

Tot5Years$asr50.54<-(Tot5Years$per50.54/100000)*5368.1

Tot5Years$asr55.59<-(Tot5Years$per55.59/100000)*4548.4

Tot5Years$asr60.64<-(Tot5Years$per60.64/100000)*3718.7

Tot5Years$asr65.69<-(Tot5Years$per65.69/100000)*2959

Tot5Years$asr70.74<-(Tot5Years$per70.74/100000)*2209.2

Tot5Years$asr75.79<-(Tot5Years$per75.79/100000)*1519.5

Tot5Years$asr80.84<-(Tot5Years$per80.84/100000)*909.7

Tot5Years$asr85.89<-(Tot5Years$per85.89/100000)*439.8

Tot5Years$asr90.94<-(Tot5Years$per90.94/100000)*150

Tot5Years$asr95.99<-(Tot5Years$per95.99/100000)*40

Tot5Years$asr100<-(Tot5Years$per100/100000)*5

Tot5Years$asr<-Tot5Years$asr0.4+Tot5Years$asr5.9+Tot5Years$asr10.14+Tot5Years$asr15.19+Tot5Years$asr20.24+Tot5Years$asr25.29+Tot5Years$asr30.34+

Tot5Years$asr35.39+Tot5Years$asr40.44+Tot5Years$asr45.49+Tot5Years$asr50.54+Tot5Years$asr55.59+Tot5Years$asr60.64+Tot5Years$asr65.69+ Tot5Years$asr70.74+Tot5Years$asr75.79+Tot5Years$asr80.84+Tot5Years$asr85.89+Tot5Years$asr90.94+Tot5Years$asr95.99+Tot5Years$asr100

Tot5Years$asr<-as.data.frame(round(as.data.frame(epi.conf(as.matrix(cbind(Tot5Years$asr,100000)), ctype = "inc.rate", method = "exact", design = 1,

conf.level = 0.95) * 100000),1))

Tot5Years<- Tot5Years[,c("Province","Number","per","asr")]

Women5Years<-as.data.frame(Women %>%

group_by(Province,Year) %>% summarise(Number,TotalPop,X0.4,X5.9,X10.14,X15.19,X20.24,X25.29,X30.34,X35.39,X40.44,X45.49,X50.54,X55.59,X60.64, X65.69,X70.74,X75.79,X80.84,X85.89,X90.94,X95.99,X100.,X0.4Pop,X5.9Pop,X10.14Pop,X15.19Pop, X20.24Pop,X25.29Pop,X30.34Pop,X35.39Pop,X40.44Pop,X45.49Pop,X50.54Pop,X55.59Pop,X60.64Pop,

X65.69Pop,X70.74Pop,X75.79Pop,X80.84Pop,X85.89Pop,X90.94Pop,X95.99Pop,X100.Pop))

Women5Years<-Women5Years %>%

group_by(Province) %>%

summarise(Number=sum(Number), TotalPop=mean(TotalPop),

X0.4=sum(X0.4),X5.9=sum(X5.9),X10.14=sum(X10.14),X15.19=sum(X15.19),X20.24=sum(X20.24),

X25.29=sum(X25.29),X30.34=sum(X30.34),X35.39=sum(X35.39),X40.44=sum(X40.44),X45.49=sum(X45.49),

X50.54=sum(X50.54),X55.59=sum(X55.59),X60.64=sum(X60.64), X65.69=sum( X65.69),X70.74=sum(X70.74),

X75.79=sum(X75.79),X80.84=sum(X80.84),X85.89=sum(X85.89),X90.94=sum(X90.94),X95.99=sum(X95.99),

X100.=sum(X100.),

X0.4Pop=mean(X0.4Pop),X5.9Pop=mean(X5.9Pop),X10.14Pop=mean(X10.14Pop),X15.19Pop=mean(X15.19Pop), X20.24Pop=mean(X20.24Pop),X25.29Pop=mean(X25.29Pop),X30.34Pop=mean(X30.34Pop),X35.39Pop=mean(X35.39Pop), X40.44Pop=mean(X40.44Pop),X45.49Pop=mean(X45.49Pop),X50.54Pop=mean(X50.54Pop),X55.59Pop=mean(X55.59Pop), X60.64Pop=mean(X60.64Pop),X65.69Pop=mean(X65.69Pop),X70.74Pop=mean(X70.74Pop),X75.79Pop=mean(X75.79Pop), X80.84Pop=mean(X80.84Pop),X85.89Pop=mean(X85.89Pop),X90.94Pop=mean(X90.94Pop),X95.99Pop=mean(X95.99Pop),

X100.Pop=mean(X100.Pop))

Women5Years$per0.4<-(Women5Years$X0.4/Women5Years$X0.4Pop)*100000

Women5Years$per5.9<-(Women5Years$X5.9/Women5Years$X5.9Pop)*100000

Women5Years$per10.14<-(Women5Years$X10.14/Women5Years$X10.14Pop)*100000

Women5Years$per15.19<-(Women5Years$X15.19/Women5Years$X15.19Pop)*100000

Women5Years$per20.24<-(Women5Years$X20.24/Women5Years$X20.24Pop)*100000

Women5Years$per25.29<-(Women5Years$X25.29/Women5Years$X25.29Pop)*100000

Women5Years$per30.34<-(Women5Years$X30.34/Women5Years$X30.34Pop)*100000

Women5Years$per35.39<-(Women5Years$X35.39/Women5Years$X35.39Pop)*100000

Women5Years$per40.44<-(Women5Years$X40.44/Women5Years$X40.44Pop)*100000

Women5Years$per45.49<-(Women5Years$X45.49/Women5Years$X45.49Pop)*100000

Women5Years$per50.54<-(Women5Years$X50.54/Women5Years$X50.54Pop)*100000

Women5Years$per55.59<-(Women5Years$X55.59/Women5Years$X55.59Pop)*100000

Women5Years$per60.64<-(Women5Years$X60.64/Women5Years$X60.64Pop)*100000

Women5Years$per65.69<-(Women5Years$X65.69/Women5Years$X65.69Pop)*100000

Women5Years$per70.74<-(Women5Years$X70.74/Women5Years$X70.74Pop)*100000

Women5Years$per75.79<-(Women5Years$X75.79/Women5Years$X75.79Pop)*100000

Women5Years$per80.84<-(Women5Years$X80.84/Women5Years$X80.84Pop)*100000

Women5Years$per85.89<-(Women5Years$X85.89/Women5Years$X85.89Pop)*100000

Women5Years$per90.94<-(Women5Years$X90.94/Women5Years$X90.94Pop)*100000

Women5Years$per95.99<-(Women5Years$X95.99/Women5Years$X95.99Pop)*100000

Women5Years$per100<-(Women5Years$X100./Women5Years$X100.Pop)*100000

Women5Years$per<-round(as.data.frame(epi.conf(as.matrix(cbind(Women5Years$Number,Women5Years$TotalPop)), ctype = "inc.rate",

method = "exact", design = 1, conf.level = 0.95) * 100000),1)

Women5Years$asr0.4<-(Women5Years$per0.4/100000)*8856.9

Women5Years$asr5.9<-(Women5Years$per5.9/100000)*8687

Women5Years$asr10.14<-(Women5Years$per10.14/100000)*8597

Women5Years$asr15.19<-(Women5Years$per15.19/100000)*8467

Women5Years$asr20.24<-(Women5Years$per20.24/100000)*8217.1

Women5Years$asr25.29<-(Women5Years$per25.29/100000)*7927.2

Women5Years$asr30.34<-(Women5Years$per30.34/100000)*7607.3

Women5Years$asr35.39<-(Women5Years$per35.39/100000)*7147.5

Women5Years$asr40.44<-(Women5Years$per40.44/100000)*6587.7

Women5Years$asr45.49<-(Women5Years$per45.49/100000)*6037.9

Women5Years$asr50.54<-(Women5Years$per50.54/100000)*5368.1

Women5Years$asr55.59<-(Women5Years$per55.59/100000)*4548.4

Women5Years$asr60.64<-(Women5Years$per60.64/100000)*3718.7

Women5Years$asr65.69<-(Women5Years$per65.69/100000)*2959

Women5Years$asr70.74<-(Women5Years$per70.74/100000)*2209.2

Women5Years$asr75.79<-(Women5Years$per75.79/100000)*1519.5

Women5Years$asr80.84<-(Women5Years$per80.84/100000)*909.7

Women5Years$asr85.89<-(Women5Years$per85.89/100000)*439.8

Women5Years$asr90.94<-(Women5Years$per90.94/100000)*150

Women5Years$asr95.99<-(Women5Years$per95.99/100000)*40

Women5Years$asr100<-(Women5Years$per100/100000)*5

Women5Years$asr<-Women5Years$asr0.4+Women5Years$asr5.9+Women5Years$asr10.14+Women5Years$asr15.19+Women5Years$asr20.24+Women5Years$asr25.29+Women5Years$asr30.34+

Women5Years$asr35.39+Women5Years$asr40.44+Women5Years$asr45.49+Women5Years$asr50.54+Women5Years$asr55.59+Women5Years$asr60.64+Women5Years$asr65.69+

Women5Years$asr70.74+Women5Years$asr75.79+Women5Years$asr80.84+Women5Years$asr85.89+Women5Years$asr90.94+Women5Years$asr95.99+Women5Years$asr100

Women5Years$asr<-as.data.frame(round(as.data.frame(epi.conf(as.matrix(cbind(Women5Years$asr,100000)), ctype = "inc.rate", method = "exact", design = 1,

conf.level = 0.95) * 100000),1))

Women5Years<- Women5Years[,c("Province","Number","per","asr")]

Men5Years<-as.data.frame(Men %>%

group_by(Province,Year) %>% summarise(Number,TotalPop,X0.4,X5.9,X10.14,X15.19,X20.24,X25.29,X30.34,X35.39,X40.44,X45.49,X50.54,X55.59,X60.64, X65.69,X70.74,X75.79,X80.84,X85.89,X90.94,X95.99,X100.,X0.4Pop,X5.9Pop,X10.14Pop,X15.19Pop, X20.24Pop,X25.29Pop,X30.34Pop,X35.39Pop,X40.44Pop,X45.49Pop,X50.54Pop,X55.59Pop,X60.64Pop,

X65.69Pop,X70.74Pop,X75.79Pop,X80.84Pop,X85.89Pop,X90.94Pop,X95.99Pop,X100.Pop))

Men5Years<-Men5Years %>%

group_by(Province) %>%

summarise(Number=sum(Number), TotalPop=mean(TotalPop),

X0.4=sum(X0.4),X5.9=sum(X5.9),X10.14=sum(X10.14),X15.19=sum(X15.19),X20.24=sum(X20.24),

X25.29=sum(X25.29),X30.34=sum(X30.34),X35.39=sum(X35.39),X40.44=sum(X40.44),X45.49=sum(X45.49),

X50.54=sum(X50.54),X55.59=sum(X55.59),X60.64=sum(X60.64), X65.69=sum( X65.69),X70.74=sum(X70.74),

X75.79=sum(X75.79),X80.84=sum(X80.84),X85.89=sum(X85.89),X90.94=sum(X90.94),X95.99=sum(X95.99),

X100.=sum(X100.), X0.4Pop=mean(X0.4Pop),X5.9Pop=mean(X5.9Pop),X10.14Pop=mean(X10.14Pop),X15.19Pop=mean(X15.19Pop), X20.24Pop=mean(X20.24Pop),X25.29Pop=mean(X25.29Pop),X30.34Pop=mean(X30.34Pop),X35.39Pop=mean(X35.39Pop), X40.44Pop=mean(X40.44Pop),X45.49Pop=mean(X45.49Pop),X50.54Pop=mean(X50.54Pop),X55.59Pop=mean(X55.59Pop), X60.64Pop=mean(X60.64Pop),X65.69Pop=mean(X65.69Pop),X70.74Pop=mean(X70.74Pop),X75.79Pop=mean(X75.79Pop), X80.84Pop=mean(X80.84Pop),X85.89Pop=mean(X85.89Pop),X90.94Pop=mean(X90.94Pop),X95.99Pop=mean(X95.99Pop),X100.Pop=mean(X100.Pop))

Men5Years$per0.4<-(Men5Years$X0.4/Men5Years$X0.4Pop)*100000

Men5Years$per5.9<-(Men5Years$X5.9/Men5Years$X5.9Pop)*100000

Men5Years$per10.14<-(Men5Years$X10.14/Men5Years$X10.14Pop)*100000

Men5Years$per15.19<-(Men5Years$X15.19/Men5Years$X15.19Pop)*100000

Men5Years$per20.24<-(Men5Years$X20.24/Men5Years$X20.24Pop)*100000

Men5Years$per25.29<-(Men5Years$X25.29/Men5Years$X25.29Pop)*100000

Men5Years$per30.34<-(Men5Years$X30.34/Men5Years$X30.34Pop)*100000

Men5Years$per35.39<-(Men5Years$X35.39/Men5Years$X35.39Pop)*100000

Men5Years$per40.44<-(Men5Years$X40.44/Men5Years$X40.44Pop)*100000

Men5Years$per45.49<-(Men5Years$X45.49/Men5Years$X45.49Pop)*100000

Men5Years$per50.54<-(Men5Years$X50.54/Men5Years$X50.54Pop)*100000

Men5Years$per55.59<-(Men5Years$X55.59/Men5Years$X55.59Pop)*100000

Men5Years$per60.64<-(Men5Years$X60.64/Men5Years$X60.64Pop)*100000

Men5Years$per65.69<-(Men5Years$X65.69/Men5Years$X65.69Pop)*100000

Men5Years$per70.74<-(Men5Years$X70.74/Men5Years$X70.74Pop)*100000

Men5Years$per75.79<-(Men5Years$X75.79/Men5Years$X75.79Pop)*100000

Men5Years$per80.84<-(Men5Years$X80.84/Men5Years$X80.84Pop)*100000

Men5Years$per85.89<-(Men5Years$X85.89/Men5Years$X85.89Pop)*100000

Men5Years$per90.94<-(Men5Years$X90.94/Men5Years$X90.94Pop)*100000

Men5Years$per95.99<-(Men5Years$X95.99/Men5Years$X95.99Pop)*100000

Men5Years$per100<-(Men5Years$X100./Men5Years$X100.Pop)*100000

Men5Years$per<-round(as.data.frame(epi.conf(as.matrix(cbind(Men5Years$Number,Men5Years$TotalPop)), ctype = "inc.rate",

method = "exact", design = 1, conf.level = 0.95) * 100000),1)

Men5Years$asr0.4<-(Men5Years$per0.4/100000)*8856.9

Men5Years$asr5.9<-(Men5Years$per5.9/100000)*8687

Men5Years$asr10.14<-(Men5Years$per10.14/100000)*8597

Men5Years$asr15.19<-(Men5Years$per15.19/100000)*8467

Men5Years$asr20.24<-(Men5Years$per20.24/100000)*8217.1

Men5Years$asr25.29<-(Men5Years$per25.29/100000)*7927.2

Men5Years$asr30.34<-(Men5Years$per30.34/100000)*7607.3

Men5Years$asr35.39<-(Men5Years$per35.39/100000)*7147.5

Men5Years$asr40.44<-(Men5Years$per40.44/100000)*6587.7

Men5Years$asr45.49<-(Men5Years$per45.49/100000)*6037.9

Men5Years$asr50.54<-(Men5Years$per50.54/100000)*5368.1

Men5Years$asr55.59<-(Men5Years$per55.59/100000)*4548.4

Men5Years$asr60.64<-(Men5Years$per60.64/100000)*3718.7

Men5Years$asr65.69<-(Men5Years$per65.69/100000)*2959

Men5Years$asr70.74<-(Men5Years$per70.74/100000)*2209.2

Men5Years$asr75.79<-(Men5Years$per75.79/100000)*1519.5

Men5Years$asr80.84<-(Men5Years$per80.84/100000)*909.7

Men5Years$asr85.89<-(Men5Years$per85.89/100000)*439.8

Men5Years$asr90.94<-(Men5Years$per90.94/100000)*150

Men5Years$asr95.99<-(Men5Years$per95.99/100000)*40

Men5Years$asr100<-(Men5Years$per100/100000)*5

Men5Years$asr<-Men5Years$asr0.4+Men5Years$asr5.9+Men5Years$asr10.14+Men5Years$asr15.19+Men5Years$asr20.24+Men5Years$asr25.29+Men5Years$asr30.34+ Men5Years$asr35.39+Men5Years$asr40.44+Men5Years$asr45.49+Men5Years$asr50.54+Men5Years$asr55.59+Men5Years$asr60.64+Men5Years$asr65.69+ Men5Years$asr70.74+Men5Years$asr75.79+Men5Years$asr80.84+Men5Years$asr85.89+Men5Years$asr90.94+Men5Years$asr95.99+Men5Years$asr100

Men5Years$asr<-as.data.frame(round(as.data.frame(epi.conf(as.matrix(cbind(Men5Years$asr,100000)), ctype = "inc.rate", method = "exact", design = 1,

conf.level = 0.95) * 100000),1))

Men5Years<- Men5Years[,c("Province","Number","per","asr")]

# plots --------------------------------------------------------------------

PerYearT<-as.data.frame( Total %>%

group_by(Year,Province) %>%

summarise(Number)

)

PerYearT<-reshape(PerYearT, idvar = "Year",timevar = "Province", direction = "wide")

PerYearT$SumT<-rowSums(PerYearT[,2:32])

PerYearT$SumPercT<-round((rowSums(PerYearT[,2:32])/75701)*100,2)

PerYearF<-as.data.frame( Women %>%

group_by(Year,Province) %>%

summarise(Number)

)

PerYearF<-reshape(PerYearF, idvar = "Year",timevar = "Province", direction = "wide")

PerYearF$SumF<-rowSums(PerYearF[,2:32])

PerYearF$SumPercF<-round((rowSums(PerYearF[,2:32])/74213)*100,2)

PerYearM<-as.data.frame( Men %>%

group_by(Year,Province) %>%

summarise(Number)

)

PerYearM<-reshape(PerYearM, idvar = "Year",timevar = "Province", direction = "wide")

PerYearM$SumM<-rowSums(PerYearM[,2:32])

PerYearM$SumPercM<-round((rowSums(PerYearM[,2:32])/1488)*100,2)

PerYear<-cbind(PerYearT[,c(1,33,34)],PerYearF[,c(33,34)],PerYearM[,c(33,34)])

IranASRPerYear$Sex<-str_replace(IranASRPerYear$Sex,"M","Men")

IranASRPerYear$Sex<-str_replace(IranASRPerYear$Sex,"F","Women")

IranASRPerYear$Sex<-str_replace(IranASRPerYear$Sex,"T","Total")

q1<-ggplot(IranASRPerYear,aes(Year,ASRTot.est,fill=Sex)) +

geom_col_pattern(

position = position_dodge2(width = 0.4, preserve = "single", padding = 0,reverse = FALSE),

aes(pattern = Sex),

pattern_angle=225,

pattern_color = "white",

pattern_fill = "black",

pattern_spacing=0.02,

pattern_density = 0.2

)+

geom_errorbar(aes(ymin =ASRTot.lower , ymax=ASRTot.upper ),

position = position_dodge2(reverse = FALSE, padding = 0.6, width = 0.5))+

guides(fill=guide_legend(title="Legend"))+

theme_bw(18)+

theme(axis.line = element_line(size=0.1, colour = "black"),

panel.grid.major = element_line(colour = "#d3d3d3",linewidth=0.5),

panel.grid.minor = element_blank(),

panel.border = element_blank(), panel.background = element_blank(),

plot.title = element_text(size = 12,face="bold"),

axis.title = element_blank(),

axis.text.x = element_blank(),

axis.text.y = element_text(size = 12,face="bold"),

strip.text =element_text(12),

legend.text=element_text(size=12),

legend.key.size = unit(2,"line"),

legend.title=element_text(size=14),

legend.position="top",

legend.background = element_rect(fill = NA))

df.table1<- ggplot(PerYear, aes(x = Year, y = 0,label = paste(SumT,"(",SumPercT,"%)"))) +

geom_text(size = 4, colour = "black") +

theme_minimal() +

scale_y_continuous(breaks=NULL, name = "Total(%)")+

theme(panel.grid.major = element_blank(), legend.position = "none",

panel.border = element_rect(colour = "black", fill=NA, size=0.5),

axis.text.x = element_blank(),

axis.text.y=element_blank(),

axis.line.x = element_line(linewidth=0.05),

axis.line.y = element_line(linewidth=0.05),

axis.ticks = element_blank(),

axis.title.x=element_blank(),

axis.title.y=element_text(angle=0,face="bold"))

df.table2 <- ggplot(PerYear, aes(x = Year, y = 0,label = paste(SumF,"(",SumPercF,"%)"))) +

geom_text(size = 4, colour = "black") +

theme_minimal() +

scale_y_continuous(breaks=NULL, name = "Women (%)")+

theme(panel.grid.major = element_blank(), legend.position = "none",

panel.border = element_rect(colour = "black", fill=NA, size=0.5),

axis.text.x = element_blank(),

axis.text.y=element_text(face="bold"),

axis.line.x = element_line(linewidth=0.05),

axis.line.y = element_line(linewidth=0.05),

axis.ticks = element_blank(),

axis.title.x=element_blank(),

axis.title.y=element_text(angle=0,face="bold"))

df.table3 <- ggplot(PerYear, aes(x = Year, y = 0,label = paste(SumM,"(",SumPercM,"%)"))) +

geom_text(size = 4, colour = "black") +

theme_minimal() +

scale_y_continuous(breaks=NULL, name = "Men (%)")+

theme(panel.grid.major = element_blank(), legend.position = "none",

panel.border = element_rect(colour = "black", fill=NA, size=0.5),

axis.text.x = element_blank(),

axis.text.y=element_text(face="bold"),

axis.line.x = element_line(linewidth=0.05),

axis.line.y = element_line(linewidth=0.05),

axis.ticks = element_blank(),

axis.title.x=element_blank(),

axis.title.y=element_text(angle=0,face="bold"))

gA <- ggplotGrob(q1)

gB <- ggplotGrob(df.table1)

gC <- ggplotGrob(df.table2)

gD <- ggplotGrob(df.table3)

maxWidth = grid::unit.pmax(gA$widths[2:3], gB$widths[2:3], gC$widths[2:3], gD$widths[2:3])

gA$widths[2:3] <- as.list(maxWidth)

gB$widths[2:3] <- as.list(maxWidth)

gC$widths[2:3] <- as.list(maxWidth)

gD$widths[2:3] <- as.list(maxWidth)

yearsplot<-ggdraw() +

draw_plot(gA, x = 0, y =0.15, width = 1, height = .895) +

draw_plot(gB, x = 0, y = 0.1, width = 1, height = .08,hjust = -0.026,halign = 0.1) +

draw_plot(gC, x = 0, y = 0.05, width = 1, height =.08,hjust = -0.026)+

draw_plot(gD, x = 0, y = 0, width = 1, height =.08,hjust = -0.026)

ggsave("yearsplot.tiff", yearsplot, width=12, height=8, dpi=300)

agegroup1<-gather(agegroup[,c(1:4)],"Agegroups","Number",-c("Agegroup"))

agegroup2<-gather(agegroup[,c(1,5:7)],"Agegroups","Number",-c("Agegroup"))

agegroup1<-cbind(agegroup1,agegroup2[,c(2,3)])

as<-as.matrix(agegroup1[,c(3,5)])

agegroup1$Per<-round(as.data.frame(epi.conf(as, ctype = "inc.rate", method = "exact", design = 1,

conf.level = 0.95) * 100000),1)

names(agegroup1)<-c("Agegroup","Sex","Number","PopSex","PopUlation","Per")

agegroup1$Percent<-ifelse(agegroup1$Sex=="Total",paste(agegroup1$Number,"(",round((agegroup1$Number/75701)*100,2),"%",")"),

ifelse(agegroup1$Sex=="Women",paste(agegroup1$Number,"(",round((agegroup1$Number/74213)*100,2),"%",")"),

ifelse(agegroup1$Sex=="Men",paste(agegroup1$Number,"(",round((agegroup1$Number/1488)*100,2),"%",")"),NA)))

q2<- ggplot(agegroup1, aes(Agegroup,Per$est,fill=Sex)) +

geom_col_pattern(

position = position_dodge2(width = 0.4, preserve = "single", padding = 0,reverse = FALSE),

aes(pattern = Sex),

pattern_angle=225,

pattern_color = "white",

pattern_fill = "black",

pattern_spacing=0.02,

pattern_density = 0.2

)+

geom_errorbar(aes(ymin =Per$lower , ymax=Per$upper ),

position = position_dodge2(reverse = FALSE, padding = 0.6, width = 0.5))+

guides(fill=guide_legend(title="Legend"))+

theme_bw(18)+

theme(axis.line = element_line(size=0.1, colour = "black"),

panel.grid.major = element_line(colour = "#d3d3d3",linewidth=0.5),

panel.grid.minor = element_blank(),

panel.border = element_blank(), panel.background = element_blank(),

plot.title = element_text(size = 14,face="bold"),

axis.title = element_blank(),

axis.text.x = element_blank(),

axis.text.y = element_text(size = 14,face="bold"),

strip.text =element_text(14),

legend.text=element_text(size=12),

legend.key.size = unit(2,"line"),

legend.title=element_text(size=14),

legend.position="top")

df.table1<- ggplot(subset(agegroup1,Sex=="Total"), aes(x = Agegroup, y = 0, label =Number)) +

geom_text(size = 4, colour = "black") +

theme_minimal() +

theme(panel.grid.major = element_blank(), legend.position = "none",

panel.border = element_rect(colour = "black", fill=NA, size=0.5),

panel.background = element_rect(color="white"),

axis.text.x = element_blank(),

axis.text.y=element_blank(),

axis.line.x = element_blank(),

axis.line.y = element_blank(),

axis.ticks = element_blank(),

axis.title.x=element_blank(),

axis.title.y=element_text(angle=0,face="bold"))

df.table2<- ggplot(subset(agegroup1,Sex=="Total"), aes(x = Agegroup, y = 0,

label =paste("(",Per$est,")"))) +

geom_text(size = 4, colour = "black") +

theme_minimal() +

theme(panel.grid.major = element_blank(), legend.position = "none",

panel.border = element_rect(colour = "black", fill=NA, size=0.5),

panel.background = element_rect(color="white"),

axis.text.x = element_blank(),

axis.text.y=element_blank(),

axis.line.x = element_blank(),

axis.line.y = element_blank(),

axis.ticks = element_blank(),

axis.title.x=element_blank(),

axis.title.y=element_text(angle=0,face="bold"))

df.table3<- ggplot(subset(agegroup1,Sex=="Women"), aes(x = Agegroup, y = 0,

label =Number)) +

geom_text(size = 4, colour = "black") +

theme_minimal() +

theme(panel.grid.major = element_blank(), legend.position = "none",

panel.border = element_rect(colour = "black", fill=NA, size=0.5),

panel.background = element_rect(color="white"),

axis.text.x = element_blank(),

axis.text.y=element_blank(),

axis.line.x = element_blank(),

axis.line.y = element_blank(),

axis.ticks = element_blank(),

axis.title.x=element_blank(),

axis.title.y=element_text(angle=0,face="bold"))

df.table4<- ggplot(subset(agegroup1,Sex=="Women"), aes(x = Agegroup, y = 0,

label =paste("(",Per$est,")"))) +

geom_text(size = 4, colour = "black") +

theme_minimal() +

theme(panel.grid.major = element_blank(), legend.position = "none",

panel.border = element_rect(colour = "black", fill=NA, size=0.5),

panel.background = element_rect(color="white"),

axis.text.x = element_blank(),

axis.text.y=element_blank(),

axis.line.x = element_blank(),

axis.line.y = element_blank(),

axis.ticks = element_blank(),

axis.title.x=element_blank(),

axis.title.y=element_text(angle=0,face="bold"))

df.table5<- ggplot(subset(agegroup1,Sex=="Men"), aes(x = Agegroup, y = 0,

label =Number)) +

geom_text(size = 4, colour = "black") +

theme_minimal() +

theme(panel.grid.major = element_blank(), legend.position = "none",

panel.border = element_rect(colour = "black", fill=NA, size=0.5),

panel.background = element_rect(color="white"),

axis.text.x = element_blank(),

axis.text.y=element_blank(),

axis.line.x = element_blank(),

axis.line.y = element_blank(),

axis.ticks = element_blank(),

axis.title.x=element_blank(),

axis.title.y=element_text(angle=0,face="bold"))

df.table6<- ggplot(subset(agegroup1,Sex=="Men"), aes(x = Agegroup, y = 0,

label =paste("(",Per$est,")"))) +

geom_text(size = 4, colour = "black") +

theme_minimal() +

theme(panel.grid.major = element_blank(), legend.position = "none",

panel.border = element_rect(colour = "black", fill=NA, size=0.5),

panel.background = element_rect(color="white"),

axis.text.x = element_blank(),

axis.text.y=element_blank(),

axis.line.x = element_blank(),

axis.line.y = element_blank(),

axis.ticks = element_blank(),

axis.title.x=element_blank(),

axis.title.y=element_text(angle=0,face="bold"))

gA <- ggplotGrob(q2)

gB <- ggplotGrob(df.table1)

gC <- ggplotGrob(df.table2)

gD <- ggplotGrob(df.table3)

gE <- ggplotGrob(df.table4)

gF <- ggplotGrob(df.table5)

gI <- ggplotGrob(df.table6)

maxWidth = grid::unit.pmax(gA$widths[2:3], gB$widths[2:3], gC$widths[2:3], gD$widths[2:3],

gE$widths[2:3], gF$widths[2:3], gI$widths[2:3])

gA$widths[2:3] <- as.list(maxWidth)

gB$widths[2:3] <- as.list(maxWidth)

gC$widths[2:3] <- as.list(maxWidth)

gD$widths[2:3] <- as.list(maxWidth)

gE$widths[2:3] <- as.list(maxWidth)

gF$widths[2:3] <- as.list(maxWidth)

gI$widths[2:3] <- as.list(maxWidth)

agesplot<-ggdraw() +

draw_plot(gA, x = 0, y =0.3, width = 1, height = .7) +

draw_plot(gB, x = 0, y = 0.25, width = 1, height = .05,hjust = -0.026,halign =-4 )+

draw_plot(gC, x = 0, y = 0.2, width = 1,height = .05,hjust = -0.026,halign =-4)+

draw_plot(gD, x = 0, y = 0.15, width = 1,height = .05,hjust = -0.026,halign =-4)+

draw_plot(gE, x = 0, y = 0.1, width = 1,height = .05,hjust = -0.026,halign =-4)+

draw_plot(gF, x = 0, y = 0.05, width = 1,height = .05,hjust = -0.026,halign =-4)+

draw_plot(gI, x = 0, y = 0, width = 1,height = .05,hjust = -0.026,halign =-4)

ggsave("Agegroups.tiff", agesplot, width=12, height=8, dpi=300)

remove(as,agegroup1,agegroup2,agesplot,as,df.table1,df.table2,df.table3,

df.table4,df.table5,df.table6,gA,gB,gC,gD,gE,gF,gI,maxWidth,PerYearF,

PerYearM,PerYearT,q1,q2,yearsplot)

# GIS Files ---------------------------------------------------------------

TotalPerProvinceYearASR<-Total[,c("Province","Year","asrT")]

WomenPerProvinceYearASR<-Women[,c("Province","Year","asrT")]

MenPerProvinceYearASR<-Men[,c("Province","Year","asrT")]

TotalPerProvinceYearASR<-reshape(TotalPerProvinceYearASR,idvar = "Province",timevar = "Year",direction = "wide")

WomenPerProvinceYearASR<-reshape(WomenPerProvinceYearASR,idvar = "Province",timevar = "Year",direction = "wide")

MenPerProvinceYearASR<-reshape(MenPerProvinceYearASR,idvar = "Province",timevar = "Year",direction = "wide")

write.xlsx(MeanASRTPerProv,"5yearsMeanASRTperProvince.xlsx")

write.xlsx(MeanASRFPerProv,"5yearsMeanASRFperProvince.xlsx")

write.xlsx(MeanASRMPerProv,"5yearsMeanASRMperProvince.xlsx")

write.xlsx(TotalPerProvinceYearASR,"TotalPerProvinceYearASR.xlsx")

write.xlsx(WomenPerProvinceYearASR,"WomenPerProvinceYearASR.xlsx")

write.xlsx(MenPerProvinceYearASR,"MenPerProvinceYearASR.xlsx")

write.csv(Tot5Years,"Tot5Years.csv")

write.csv(Women5Years,"Women5Years.csv")

write.csv(Men5Years,"Men5Years.csv")

# Relative Risk among clusters --------------------------------------------

asr<-read.xlsx("ASR.xlsx")

asr<-gather(asr,"Type","ASR",-c(1,2))

write.xlsx(asr,"ASRGatherd.xlsx")

asr<-read.xlsx("ASRGatherd.xlsx",sheet = "Sheet1")

RR<-data.frame("Type"=c("5yr ASR.","Women's 5yr ASR.","Men's 5yr ASR.","ASR avg.","Women's ASR avg.","Men's ASR avg.",

"ASR in 2014","ASR in 2015","ASR in 2016","ASR in 2017","ASR in 2018",

"Women's ASR in 2014","Women's ASR in 2015","Women's ASR in 2016","Women's ASR in 2017","Women's ASR in 2018",

"Men's ASR in 2014","Men's ASR in 2015","Men's ASR in 2016","Men's ASR in 2017","Men's ASR in 2018",),

"HH vs.LL RR"=c(mean(asr$ASR[asr$Type=="5yr ASR." & asr$Cluster=="HH"])/mean(asr$ASR[asr$Type=="5yr ASR." & asr$Cluster=="LL"]),

mean(asr$ASR[asr$Type=="Women's 5yr ASR." & asr$Cluster=="HH"])/mean(asr$ASR[asr$Type=="Women's 5yr ASR." & asr$Cluster=="LL"]),

mean(asr$ASR[asr$Type=="Men's 5yr ASR." & asr$Cluster=="HH"])/mean(asr$ASR[asr$Type=="Men's 5yr ASR." & asr$Cluster=="LL"]),

mean(asr$ASR[asr$Type=="ASR avg." & asr$Cluster=="HH"])/mean(asr$ASR[asr$Type=="ASR avg." & asr$Cluster=="LL"]),

mean(asr$ASR[asr$Type=="Women's ASR avg." & asr$Cluster=="HH"])/mean(asr$ASR[asr$Type=="Women's ASR avg." & asr$Cluster=="LL"]),

mean(asr$ASR[asr$Type=="Men's ASR avg." & asr$Cluster=="HH"])/mean(asr$ASR[asr$Type=="Men's ASR avg." & asr$Cluster=="LL"])),

"HH vs. NS RR"=c(mean(asr$ASR[asr$Type=="5yr ASR." & asr$Cluster=="HH"])/mean(asr$ASR[asr$Type=="5yr ASR." & asr$Cluster=="NS"]),

mean(asr$ASR[asr$Type=="Women's 5yr ASR." & asr$Cluster=="HH"])/mean(asr$ASR[asr$Type=="Women's 5yr ASR." & asr$Cluster=="NS"]),

mean(asr$ASR[asr$Type=="Men's 5yr ASR." & asr$Cluster=="HH"])/mean(asr$ASR[asr$Type=="Men's 5yr ASR." & asr$Cluster=="NS"]),

mean(asr$ASR[asr$Type=="ASR avg." & asr$Cluster=="HH"])/mean(asr$ASR[asr$Type=="ASR avg." & asr$Cluster=="NS"]),

mean(asr$ASR[asr$Type=="Women's ASR avg." & asr$Cluster=="HH"])/mean(asr$ASR[asr$Type=="Women's ASR avg." & asr$Cluster=="NS"]),

mean(asr$ASR[asr$Type=="Men's ASR avg." & asr$Cluster=="HH"])/mean(asr$ASR[asr$Type=="Men's ASR avg." & asr$Cluster=="NS"])),

"LL vs. NS RR"=c(mean(asr$ASR[asr$Type=="5yr ASR." & asr$Cluster=="LL"])/mean(asr$ASR[asr$Type=="5yr ASR." & asr$Cluster=="NS"]),

mean(asr$ASR[asr$Type=="Women's 5yr ASR." & asr$Cluster=="LL"])/mean(asr$ASR[asr$Type=="Women's 5yr ASR." & asr$Cluster=="NS"]),

mean(asr$ASR[asr$Type=="Men's 5yr ASR." & asr$Cluster=="LL"])/mean(asr$ASR[asr$Type=="Men's 5yr ASR." & asr$Cluster=="NS"]),

mean(asr$ASR[asr$Type=="ASR avg." & asr$Cluster=="LL"])/mean(asr$ASR[asr$Type=="ASR avg." & asr$Cluster=="NS"]),

mean(asr$ASR[asr$Type=="Women's ASR avg." & asr$Cluster=="LL"])/mean(asr$ASR[asr$Type=="Women's ASR avg." & asr$Cluster=="NS"]),

mean(asr$ASR[asr$Type=="Men's ASR avg." & asr$Cluster=="LL"])/mean(asr$ASR[asr$Type=="Men's ASR avg." & asr$Cluster=="NS"])),

"LL vs. HH RR"=c(mean(asr$ASR[asr$Type=="5yr ASR." & asr$Cluster=="LL"])/mean(asr$ASR[asr$Type=="5yr ASR." & asr$Cluster=="HH"]),

mean(asr$ASR[asr$Type=="Women's 5yr ASR." & asr$Cluster=="LL"])/mean(asr$ASR[asr$Type=="Women's 5yr ASR." & asr$Cluster=="HH"]),

mean(asr$ASR[asr$Type=="Men's 5yr ASR." & asr$Cluster=="LL"])/mean(asr$ASR[asr$Type=="Men's 5yr ASR." & asr$Cluster=="HH"]),

mean(asr$ASR[asr$Type=="ASR avg." & asr$Cluster=="LL"])/mean(asr$ASR[asr$Type=="ASR avg." & asr$Cluster=="HH"]),

mean(asr$ASR[asr$Type=="Women's ASR avg." & asr$Cluster=="LL"])/mean(asr$ASR[asr$Type=="Women's ASR avg." & asr$Cluster=="HH"]),

mean(asr$ASR[asr$Type=="Men's ASR avg." & asr$Cluster=="LL"])/mean(asr$ASR[asr$Type=="Men's ASR avg." & asr$Cluster=="HH"])))

# create a data frame with the relevant subsets

asr_subset <- asr %>%

filter(Type %in% c("5yr ASR.", "Women's 5yr ASR.", "Men's 5yr ASR.", "ASR avg.", "Women's ASR avg.",

"Men's ASR avg.", "ASR in 2014", "ASR in 2015", "ASR in 2016", "ASR in 2017", "ASR in 2018"),

cluster %in% c("HH", "LL", "NS"))

# calculate means using dplyr

RR <- asr_subset %>%

group_by(Type) %>%

summarize(`HH vs.LL RR` = mean(ASR[cluster == "HH"]) / mean(ASR[cluster == "LL"]),

`HH vs. NS RR` = mean(ASR[cluster == "HH"]) / mean(ASR[cluster == "NS"]),

`LL vs. NS RR` = mean(ASR[cluster == "LL"]) / mean(ASR[cluster == "NS"]))

RR<-gather(RR,"Types","Value",-c("Type"))

se_rr <- 0.1

# Set the confidence level

alpha <- 0.05

# Calculate the z-score for the desired level of confidence

z <- qnorm(1 - alpha/2)

RR$Low <- RR$Value - z*se_rr

RR$High <- RR$Value + z*se_rr

RR$Types<-paste(RR$Type,RR$Types)

RR<-RR[complete.cases(RR),]

RR[,c(3:5)]<-apply(RR[,c(3:5)],2,round,digit=2)

# Define the plot theme

p1 <- ggplot(RR, aes(x=Value, y=Types)) +

geom_point(size=4, aes(color=Type)) +

geom_errorbarh(aes(xmin=Low, xmax=High)) +

geom_vline(xintercept=1, linetype="dashed", color="gray50") +

scale_x_log10(limits=c(0.3,3), expand=c(0,0),breaks=c(0,0.5,1,2)) +

theme_bw() +

ylab("") + xlab("Relative Risk (RR)")

p1 + theme(axis.text.x = element_text(face="bold", size=13),

axis.text.y = element_text(face="bold", size=13),

axis.title.x = element_text(face="bold"))

# add color legend

color_legend <- get_legend(p1 + guides(color=guide_legend(title="Group")))

# arrange plot and legend

grid.arrange(p1 + theme(legend.position="none"), color_legend, widths=c(4,1), ncol=2)

# Pearson Correlation each independet variable on ASR -----------------------------------------

PTot<-Total[,c(170,51:125)]

results <- lapply(PTot[,1:76], function(x) {

cor.test(PTot$asrT, x, method="pearson", use="pairwise.complete.obs")

})

p_values <-format(round(sapply(results, function(x) x$p.value),4),4,scientific = FALSE)

cor_coef <- round(sapply(results, function(x) x$estimate),2)

cor_df_tot <- data.frame(variable=colnames(PTot)[1:76], p_value=p_values, cor_coef=cor_coef)

remove(results,p_values,cor_coef)

PF<-Women[,c(170,51:125)]

results <- lapply(PF[,1:76], function(x) {

cor.test(PF$asrT, x, method="pearson", use="pairwise.complete.obs")

})

p_values <-format(round(sapply(results, function(x) x$p.value),4),4,scientific = FALSE)

cor_coef <- round(sapply(results, function(x) x$estimate),2)

cor_df_F <- data.frame(variable=colnames(PF)[1:76], p_value=p_values, cor_coef=cor_coef)

remove(results,p_values,cor_coef)

PM<-Men[,c(170,51:125)]

results <- lapply(PM[,1:76], function(x) {

cor.test(PM$asrT, x, method="pearson", use="pairwise.complete.obs")

})

p_values <-format(round(sapply(results, function(x) x$p.value),4),4,scientific = FALSE)

cor_coef <- round(sapply(results, function(x) x$estimate),2)

cor_df_M <- data.frame(variable=colnames(PM)[1:76], p_value=p_values, cor_coef=cor_coef)

remove(results,p_values,cor_coef)

Pearson_Coef<-cbind(cor_df_tot,cor_df_F[,2:3],cor_df_M[,2:3])

names(Pearson_Coef)<-c("Variable","P_value_Total","COefficient_Total","P_value_Women",

"COefficient_Women","P_value_Men","COefficient_Men")

remove(PF,results,p_values,cor_coef,cor_df_tot,cor_df_F,cor_df_M)

view(Pearson_Coef)

# Feature Selection Section For Gender-Intergated Population

# Lasso eldge reggresion --------------------------------------------------

ptot<-Total[,c(170,72:125)]

ptot$asrT[ptot$asrT==0]<-0.001

ptot[is.na(ptot)]<-0

# Train/Test split Ames Iowa housing dataset

set.seed(2345) # used to return consistent values for repeatability

ames_split <- initial_split(ptot, prop = .7, strata = "asrT")

ames_train <- training(ames_split)

ames_test <- testing(ames_split)

#Create dummy variables & taking the log

set.seed(2345)

ames_train_x <- model.matrix(asrT ~ ., ames_train)[, -1]

ames_train_y <- log(ames_train$asrT)

ames_test_x <- model.matrix(asrT ~ ., ames_test)[, -1]

ames_test_y <- log(ames_test$asrT)

#library(reshape2)

#library(plotly)

#Heatmap of variables to evaluate correlation

set.seed(2345)

ptot_cupdated <- ptot

cc2=cor(ptot_cupdated)

cc2_melt=melt(cc2)

gz=ggplot(cc2_melt,mapping=aes(x=Var1,y=Var2,fill=value))+

geom_tile()+

theme(axis.text.x = element_text(angle = 90, hjust = 1))+

theme(text = element_text(size=8))+

ggtitle("Heat Map for Housing Data: Numeric Predictors")+

ylab("")+

xlab("")+

scale_fill_distiller(palette = "Spectral")

ggplotly(gz, tooltip="text")

#library(caret)

#find variables with Correlation two by two by threshold above==0.9

findCorrelation(

cor(ptot_cupdated),

cutoff = 0.9,

verbose = TRUE,

names = TRUE,

exact = ncol(cor(ptot_cupdated)) < 100

)

#plot correlations plot

options(repr.plot.width=13, repr.plot.height=12) # adjust x,y size of plot

ggcorrplot(cc2, hc.order = TRUE, type = "lower",

lab = TRUE)+ggtitle('Correlation Plot of Ames Housing')

# Ridge Regression

options(repr.plot.width=10, repr.plot.height=8) # adjust x,y size of plot

ames_train_x <- scale(ames_train_x)

ames_test_x <- scale(ames_test_x)

ames_IA_ridge <- glmnet(

x = ames_train_x,

y = ames_train_y,

alpha = 0

)

plot(ames_IA_ridge, xvar = "lambda")

ridge_<- glmnet(x = ames_train_x, y = ames_train_y, alpha = 0)

sprintf('Max Lambda: %.3f',max(ridge_$lambda))

sprintf('Min Lambda: %.3f',min(ridge_$lambda))

#Find best values for lambda!

set.seed(2345)

ames_ridge_cv <- cv.glmnet(

x = ames_train_x,

y = ames_train_y,

alpha = 0

)

# plot results

plot(ames_ridge_cv)

#Training Our Model with CV using Ridge

mes_IA_ridge <- glmnet(x = ames_train_x,y = ames_train_y,alpha = 0)

# Tuning:

fit.ridge.cv <- cv.glmnet(ames_train_x, ames_train_y, type.measure="mse", alpha=0)

# Predicting Values:

pred_fit_ridge_train <- predict(fit.ridge.cv, ames_train_x, s = "lambda.min")

pred_fit_ridge_test <- predict(ames_IA_ridge, ames_test_x, s =fit.ridge.cv$lambda.min )

print('RIDGE:')

sprintf('Test RMSE: %.4f',sqrt(mean((pred_fit_ridge_test-ames_test_y)^2)))

sprintf('Train RMSE: %.4f',sqrt(mean((pred_fit_ridge_train-ames_train_y)^2)))

print('Your Vertical Lines')

r_squared_train <- cor(pred_fit_ridge_train, ames_train_y)^2

r_squared_test<- cor(pred_fit_ridge_test, ames_test_y)^2

sprintf('Test R_squared: %.4f',r_squared_test)

sprintf('Train R_squared: %.4f',r_squared_train)

min_mse_ridge_cv<-min(fit.ridge.cv$cvm) # minimum MSE

lambda_for_min_mse<-fit.ridge.cv$lambda.min # lambda for this min MSE

#1 std_err for min MSE

minMSE_1stderr_ridge<-fit.ridge.cv$cvm[fit.ridge.cv$lambda == fit.ridge.cv$lambda.1se]

lambda_for_minMSe_1stderr_ridge<-fit.ridge.cv$lambda.1se # lambda for this MSE

sprintf('Minimum MSE for CV: %.4f',min_mse_ridge_cv)

sprintf('Lambda for Corresponding Min. MSE : %.4f',lambda_for_min_mse)

sprintf('Min MSE for 1st Error: %.4f',minMSE_1stderr_ridge)

sprintf('Corresponding Lambda for 1 Std Err : %.4f',lambda_for_minMSe_1stderr_ridge)

sprintf('Log(Lambda Min. MSE): %.4f',log(fit.ridge.cv$lambda.min))

sprintf('Log(Lambda 1 Std Error MSE): %.4f',log(fit.ridge.cv$lambda.1se))

options(repr.plot.width=10, repr.plot.height=8) # adjust x,y size of plot

set.seed(2345)

ames_ridge_min <- glmnet(

x = ames_train_x,

y = ames_train_y,

alpha = 0

)

plot(ames_ridge_min, xvar = "lambda",main = 'Ridge: ')

abline(v = log(ames_ridge_cv$lambda.1se), col = "red", lty = "dashed")

abline(v = log(ames_ridge_cv$lambda.min), col = "red", lty = "dashed")

options(repr.plot.width=10, repr.plot.height=8) # adjust x,y size of plot

ames_ridge_cv<-coef(ames_ridge_cv, s = "lambda.min")

ames_ridge_cv<-as.data.frame(ames_ridge_cv[-1,])

ames_ridge_cv$row<-rownames(ames_ridge_cv)

names(ames_ridge_cv)<-c("row","value")

ames_ridge_cv$Coeff<-format(ames_ridge_cv$row,digit=4,scientific=FALSE)

# Ridge regression coefficients for scaled data

coef_ridge_scaled <- coef(fit.ridge.cv, s = lambda_for_min_mse)

# Unscale coefficients for original data

coef_ridge_unscaled <- coef_ridge_scaled[-1] / sd(ames_train_x)

coef_ridge_unscaled <- c(coef_ridge_scaled[1], coef_ridge_unscaled)

coef_ridge_unscaled<-as.data.frame(coef_ridge_unscaled[-1])

coef_ridge_unscaled$value<-rownames(ames_ridge_cv)

names(coef_ridge_unscaled)<-c("row","value")

coef_ridge_unscaled$Coeff<-format(coef_ridge_unscaled$row,digit=4,scientific=FALSE)

coef_ridge_unscaled$scaled_coeff<-ames_ridge_cv$Coeff

coef_ridge_unscaled$Coeffs<- abs(coef_ridge_unscaled$row)

VairablesSelectedRidge<-coef_ridge_unscaled[order(desc(coef_ridge_unscaled$Coeffs)),]

VairablesSelectedRidge<-data.frame(value=VairablesSelectedRidge[1:10,2])

ggplot() +

geom_point(coef_ridge_unscaled,mapping=aes(row, reorder(value,row), color = value > 0)) +

ggtitle("Influential variables") +

xlab("Coefficient") +

ylab(NULL)

# explanatory Regression --------------------------------------------------

HighCorr<-data.frame(Var1=findCorrelation(

cor(ptot_cupdated),

cutoff = 0.9,

verbose = TRUE,

names = TRUE,

exact = ncol(cor(ptot_cupdated)) < 100

))

HighCorr$Index <- lapply(HighCorr$Var1, function(x) which(colnames(Total) == x))

library(car)

library(dplyr)

# Select columns of interest

dfExpl <- Total[, c(170, 72:105, 107:111, 113:125)]

dfExpl$asrT[dfExpl$asrT==0] <- 0.001

dfExpl[is.na(dfExpl)] <- 0

# Set the random seed for reproducibility

set.seed(123)

# Create 10-fold cross-validation indices

folds <- createFolds(dfExpl$asrT, k = 10, list = TRUE, returnTrain = FALSE)

# Initialize vectors and data frames to store the results

rmseTrainExp <- rep(0, 10)

rmseTestExp <- rep(0, 10)

rsqTrainExp <- rep(0, 10)

rsqTestExp <- rep(0, 10)

vifList <- list()

coefList <- list()

tvalList <- list()

pvalList <- list()

# Loop over the folds

for (i in 1:10) {

# Get the indices for the current fold

testIndexExp <- folds[[i]]

trainIndexExp <- setdiff(1:nrow(dfExpl), testIndexExp)

# Split the data into train and test sets for the current fold

trainDataExp <- dfExpl[trainIndexExp, ]

testDataExp <- dfExpl[testIndexExp, ]

# Fit the model using the train dataset for the current fold

fitExp <- lm(asrT ~ ., data = trainDataExp)

# Store the VIF, coefficients, t-values, and p-values for each variable

vifList[[i]] <- data.frame(variables = names(vif(fitExp)),

vif = vif(fitExp))

coefList[[i]] <- coef(fitExp)

tvalList[[i]] <- summary(fitExp)$coefficients[, "t value"]

pvalList[[i]] <- summary(fitExp)$coefficients[, "Pr(>|t|)"]

# Predict the test data using the fitted model

predTestExp <- predict(fitExp, newdata = testDataExp)

# Calculate RMSE for train and test datasets for the current fold

rmseTrainExp[i] <- sqrt(mean((trainDataExp$asrT - predict(fitExp, trainDataExp))^2))

rmseTestExp[i] <- sqrt(mean((testDataExp$asrT - predTestExp)^2))

# Calculate r-squared for train and test datasets for the current fold

rsqTrainExp[i] <- summary(fitExp)$r.squared

rsqTestExp[i] <- cor(testDataExp$asrT, predTestExp)^2

}

# Calculate the average RMSE and r-squared over the 10 folds

avgRmseTrainExp <- mean(rmseTrainExp)

avgRmseTestExp <- mean(rmseTestExp)

avgRsqTrainExp <- mean(rsqTrainExp)

avgRsqTestExp <- mean(rsqTestExp)

# Print the results

cat("Average train RMSE: ", avgRmseTrainExp, "\n")

cat("Average test RMSE: ", avgRmseTestExp, "\n")

cat("Average train R-squared: ", avgRsqTrainExp, "\n")

cat("Average test R-squared: ", avgRsqTestExp, "\n")

# Calculate the mean VIF, coefficients, t-values, and p-values for each variable over the 10 folds

vifMean <- data.frame(variables = unique(vifList[[1]]$variables),

vif = unlist(lapply(unique(vifList[[1]]$variables),

function(v) mean(vifList[[1]][vifList[[1]]$variables == v, "vif"]))))

coefList<-as.data.frame(coefList)

tvalList<-as.data.frame(tvalList)

pvalList<-as.data.frame(pvalList)

coefList$mean <- rowMeans(coefList)

tvalList$mean <- rowMeans(tvalList)

pvalList$mean <- rowMeans(pvalList)

ModelExpSumm <- data.frame(Variable = vifMean$variables,

coefMean = coefList$mean[-1],

tvalMean = tvalList$mean[-1],

pvalMean = pvalList$mean[-1],

VIF = vifMean$vif)

# Identify variables with VIF < 7.5

VariablesforOLSlower7.5<-subset(ModelExpSumm,VIF<7.5)

VariablesforOLSlower7.5$index<-match(VariablesforOLSlower7.5$Variable,colnames(Total))

VariablesforOLS<-VariablesforOLSlower7.5$index

# OLS Regression --------------------------------------------------

dfOLS<-Total[,c(170,VariablesforOLS)]

dfOLS$asrT[dfOLS$asrT==0]<-0.001

dfOLS[is.na(dfOLS)]<-0

# Set the random seed for reproducibility

set.seed(123)

# Create 10-fold cross-validation indices

folds <- createFolds(dfOLS$asrT, k = 10, list = TRUE, returnTrain = FALSE)

# Initialize vectors and data frames to store the results

rmseTrainOLS <- rep(0, 10)

rmseTestOLS <- rep(0, 10)

rsqTrainOLS <- rep(0, 10)

rsqTestOLS <- rep(0, 10)

vifList <- list()

coefList <- list()

tvalList <- list()

pvalList <- list()

# Loop over the folds

for (i in 1:10) {

# Get the indices for the current fold

testIndexOLS <- folds[[i]]

trainIndexOLS <- setdiff(1:nrow(dfOLS), testIndexOLS)

# Split the data into train and test sets for the current fold

trainDataOLS <- dfOLS[trainIndexOLS, ]

testDataOLS <- dfOLS[testIndexOLS, ]

# Fit the model using the train dataset for the current fold

fitOLS <- lm(asrT ~ ., data = trainDataOLS)

# Store the VIF, coefficients, t-values, and p-values for each variable

vifList[[i]] <- data.frame(variables = names(vif(fitOLS)),

vif = vif(fitOLS))

coefList[[i]] <- coef(fitOLS)

tvalList[[i]] <- summary(fitOLS)$coefficients[, "t value"]

pvalList[[i]] <- summary(fitOLS)$coefficients[, "Pr(>|t|)"]

# Predict the test data using the fitted model

predTestOLS <- predict(fitOLS, newdata = testDataOLS)

# Calculate RMSE for train and test datasets for the current fold

rmseTrainOLS[i] <- sqrt(mean((trainDataOLS$asrT - predict(fitOLS, trainDataOLS))^2))

rmseTestOLS[i] <- sqrt(mean((testDataOLS$asrT - predTestOLS)^2))

# Calculate r-squared for train and test datasets for the current fold

rsqTrainOLS[i] <- summary(fitOLS)$r.squared

rsqTestOLS[i] <- cor(testDataOLS$asrT, predTestOLS)^2

}

# Calculate the average RMSE and r-squared over the 10 folds

avgRmseTrainOLS <- mean(rmseTrainOLS)

avgRmseTestOLS <- mean(rmseTestOLS)

avgRsqTrainOLS <- mean(rsqTrainOLS)

avgRsqTestOLS <- mean(rsqTestOLS)

# Print the results

cat("Average train RMSE: ", avgRmseTrainOLS, "\n")

cat("Average test RMSE: ", avgRmseTestOLS, "\n")

cat("Average train R-squared: ", avgRsqTrainOLS, "\n")

cat("Average test R-squared: ", avgRsqTestOLS, "\n")

# Calculate the mean VIF, coefficients, t-values, and p-values for each variable over the 10 folds

vifMean <- data.frame(variables = unique(vifList[[1]]$variables),

vif = unlist(lapply(unique(vifList[[1]]$variables),

function(v) mean(vifList[[1]][vifList[[1]]$variables == v, "vif"]))))

coefList<-as.data.frame(coefList)

tvalList<-as.data.frame(tvalList)

pvalList<-as.data.frame(pvalList)

coefList$mean <- rowMeans(coefList)

tvalList$mean <- rowMeans(tvalList)

pvalList$mean <- rowMeans(pvalList)

ModelOLSSumm <- data.frame(Variable = vifMean$variables,

coefMean = coefList$mean[-1],

tvalMean = tvalList$mean[-1],

pvalMean = pvalList$mean[-1],

VIF = vifMean$vif)

# Select variables based on criteria

selected_vars <- subset(ModelOLSSumm,((pvalMean < 0.05 | abs(tvalMean) > 2 ) & VIF<7.5))

# Selected Variables for Stepwise ----------------------------------------------

dfStepwise <- merge(selected_vars, VairablesSelectedRidge, by.x = "Variable", by.y = "value", all.x = TRUE, all.y=TRUE)

dfStepwise<-as.data.frame(dfStepwise[,1])

dfStepwise$index<-match(dfStepwise$`dfStepwise[, 1]` ,colnames(Total))

dfStepwise<-Total[,c(170,dfStepwise$index)]

colnames(dfStepwise)# Define your dependent variable

y <- dfStepwise$asrT

# Define your independent variables

x <- dfStepwise[, -c(1)] # Excluding the first column (assuming it contains row IDs)

# Create an initial model with all independent variables

model <- lm(y ~ ., data = x)

# Perform forward stepwise selection

step.model <- stepAIC(model, direction = "forward")

#Perform backward stepwise selection

step.model <- stepAIC(model, direction = "backward")

# Print the results

summary(step.model)

# Final variables for GWR Total -------------------------------------------

FinalVariablesTotal<-match(names(step.model$coefficients)[-1],colnames(Total))

FinalVariablesTotal<-Total[,c(1,2,170,FinalVariablesTotal)]

FinalVariablesTotal_scaled <- scale(FinalVariablesTotal[, 3:ncol(FinalVariablesTotal)])

FinalVariablesTotal <- cbind(FinalVariablesTotal[, 1:2], FinalVariablesTotal_scaled)

FinalVariablesTotal2014<-subset(FinalVariablesTotal,Year=="2014",-c(2))

FinalVariablesTotal2015<-subset(FinalVariablesTotal,Year=="2015",-c(2))

FinalVariablesTotal2016<-subset(FinalVariablesTotal,Year=="2016",-c(2))

FinalVariablesTotal2017<-subset(FinalVariablesTotal,Year=="2017",-c(2))

FinalVariablesTotal2018<-subset(FinalVariablesTotal,Year=="2018",-c(2))

write.xlsx(FinalVariablesTotal2014,"FinalVariablesTotal2014.xlsx")

write.xlsx(FinalVariablesTotal2015,"FinalVariablesTotal2015.xlsx")

write.xlsx(FinalVariablesTotal2016,"FinalVariablesTotal2016.xlsx")

write.xlsx(FinalVariablesTotal2017,"FinalVariablesTotal2017.xlsx")

write.xlsx(FinalVariablesTotal2018,"FinalVariablesTotal2018.xlsx")

FinalVariablesTotalMean<- FinalVariablesTotal %>%

group_by(Province) %>%

summarise_all(mean)

write.xlsx(FinalVariablesTotalMean,"FinalVariablesTotalMean.xlsx")

Tot5Years1<-merge(Tot5Years,FinalVariablesTotalMean[,c(1,4:12)], by="Province")

Women5Years1<-merge(Women5Years,FinalVariablesWomenMean[,c(1,4:9)], by="Province")

write.csv(Tot5Years1,"Tot5Years1.csv")

write.csv(Women5Years1,"Women5Years1.csv")

# Feature Selection Section For Women Population

# Lasso eldge reggresion --------------------------------------------------

ptot<-Women[,c(170,72:125)]

ptot$asrT[ptot$asrT==0]<-0.001

ptot[is.na(ptot)]<-0

# Train/Test split Ames Iowa housing dataset

set.seed(2345) # used to return consistent values for repeatability

ames_split <- initial_split(ptot, prop = .7, strata = "asrT")

ames_train <- training(ames_split)

ames_test <- testing(ames_split)

#Create dummy variables & taking the log

set.seed(2345)

ames_train_x <- model.matrix(asrT ~ ., ames_train)[, -1]

ames_train_y <- log(ames_train$asrT)

ames_test_x <- model.matrix(asrT ~ ., ames_test)[, -1]

ames_test_y <- log(ames_test$asrT)

#library(reshape2)

#library(plotly)

#Heatmap of variables to evaluate correlation

set.seed(2345)

ptot_cupdated <- ptot

cc2=cor(ptot_cupdated)

cc2_melt=melt(cc2)

gz=ggplot(cc2_melt,mapping=aes(x=Var1,y=Var2,fill=value))+

geom_tile()+

theme(axis.text.x = element_text(angle = 90, hjust = 1))+

theme(text = element_text(size=8))+

ggtitle("Heat Map for Housing Data: Numeric Predictors")+

ylab("")+

xlab("")+

scale_fill_distiller(palette = "Spectral")

ggplotly(gz, tooltip="text")

#library(caret)

#find variables with Correlation two by two by threshold above==0.9

findCorrelation(

cor(ptot_cupdated),

cutoff = 0.9,

verbose = TRUE,

names = TRUE,

exact = ncol(cor(ptot_cupdated)) < 100

)

#plot correlations plot

options(repr.plot.width=13, repr.plot.height=12) # adjust x,y size of plot

ggcorrplot(cc2, hc.order = TRUE, type = "lower",

lab = TRUE)+ggtitle('Correlation Plot of Ames Housing')

# Ridge Regression

options(repr.plot.width=10, repr.plot.height=8) # adjust x,y size of plot

ames_train_x <- scale(ames_train_x)

ames_test_x <- scale(ames_test_x)

ames_IA_ridge <- glmnet(

x = ames_train_x,

y = ames_train_y,

alpha = 0

)

plot(ames_IA_ridge, xvar = "lambda")

ridge_<- glmnet(x = ames_train_x, y = ames_train_y, alpha = 0)

sprintf('Max Lambda: %.3f',max(ridge_$lambda))

sprintf('Min Lambda: %.3f',min(ridge_$lambda))

#Find best values for lambda!

set.seed(2345)

ames_ridge_cv <- cv.glmnet(

x = ames_train_x,

y = ames_train_y,

alpha = 0

)

# plot results

plot(ames_ridge_cv)

#Training Our Model with CV using Ridge

mes_IA_ridge <- glmnet(x = ames_train_x,y = ames_train_y,alpha = 0)

# Tuning:

fit.ridge.cv <- cv.glmnet(ames_train_x, ames_train_y, type.measure="mse", alpha=0)

# Predicting Values:

pred_fit_ridge_train <- predict(fit.ridge.cv, ames_train_x, s = "lambda.min")

pred_fit_ridge_test <- predict(ames_IA_ridge, ames_test_x, s =fit.ridge.cv$lambda.min )

print('RIDGE:')

sprintf('Test RMSE: %.4f',sqrt(mean((pred_fit_ridge_test-ames_test_y)^2)))

sprintf('Train RMSE: %.4f',sqrt(mean((pred_fit_ridge_train-ames_train_y)^2)))

print('Your Vertical Lines')

r_squared_train <- cor(pred_fit_ridge_train, ames_train_y)^2

r_squared_test<- cor(pred_fit_ridge_test, ames_test_y)^2

min_mse_ridge_cv<-min(fit.ridge.cv$cvm) # minimum MSE

lambda_for_min_mse<-fit.ridge.cv$lambda.min # lambda for this min MSE

#1 std_err for min MSE

minMSE_1stderr_ridge<-fit.ridge.cv$cvm[fit.ridge.cv$lambda == fit.ridge.cv$lambda.1se]

lambda_for_minMSe_1stderr_ridge<-fit.ridge.cv$lambda.1se # lambda for this MSE

sprintf('Minimum MSE for CV: %.4f',min_mse_ridge_cv)

sprintf('Lambda for Corresponding Min. MSE : %.4f',lambda_for_min_mse)

sprintf('Min MSE for 1st Error: %.4f',minMSE_1stderr_ridge)

sprintf('Corresponding Lambda for 1 Std Err : %.4f',lambda_for_minMSe_1stderr_ridge)

sprintf('Log(Lambda Min. MSE): %.4f',log(fit.ridge.cv$lambda.min))

sprintf('Log(Lambda 1 Std Error MSE): %.4f',log(fit.ridge.cv$lambda.1se))

options(repr.plot.width=10, repr.plot.height=8) # adjust x,y size of plot

set.seed(2345)

ames_ridge_min <- glmnet(

x = ames_train_x,

y = ames_train_y,

alpha = 0

)

plot(ames_ridge_min, xvar = "lambda",main = 'Ridge: ')

abline(v = log(ames_ridge_cv$lambda.1se), col = "red", lty = "dashed")

abline(v = log(ames_ridge_cv$lambda.min), col = "red", lty = "dashed")

options(repr.plot.width=10, repr.plot.height=8) # adjust x,y size of plot

ames_ridge_cv<-coef(ames_ridge_cv, s = "lambda.min")

ames_ridge_cv<-as.data.frame(ames_ridge_cv[-1,])

ames_ridge_cv$row<-rownames(ames_ridge_cv)

names(ames_ridge_cv)<-c("row","value")

ames_ridge_cv$Coeff<-format(ames_ridge_cv$row,digit=4,scientific=FALSE)

# Ridge regression coefficients for scaled data

coef_ridge_scaled <- coef(fit.ridge.cv, s = lambda_for_min_mse)

# Unscale coefficients for original data

coef_ridge_unscaled <- coef_ridge_scaled[-1] / sd(ames_train_x)

coef_ridge_unscaled <- c(coef_ridge_scaled[1], coef_ridge_unscaled)

coef_ridge_unscaled<-as.data.frame(coef_ridge_unscaled[-1])

coef_ridge_unscaled$value<-rownames(ames_ridge_cv)

names(coef_ridge_unscaled)<-c("row","value")

coef_ridge_unscaled$Coeff<-format(coef_ridge_unscaled$row,digit=4,scientific=FALSE)

coef_ridge_unscaled$scaled_coeff<-ames_ridge_cv$Coeff

coef_ridge_unscaled$Coeffs<- abs(coef_ridge_unscaled$row)

VairablesSelectedRidge<-coef_ridge_unscaled[order(desc(coef_ridge_unscaled$Coeffs)),]

VairablesSelectedRidge<-data.frame(value=VairablesSelectedRidge[1:10,2])

ggplot() +

geom_point(coef_ridge_unscaled,mapping=aes(row, reorder(value,row), color = value > 0)) +

ggtitle("Influential variables") +

xlab("Coefficient") +

ylab(NULL)

# explanatory Regression --------------------------------------------------

HighCorr<-data.frame(Var1=findCorrelation(

cor(ptot_cupdated),

cutoff = 0.9,

verbose = TRUE,

names = TRUE,

exact = ncol(cor(ptot_cupdated)) < 100

))

HighCorr$Index <- lapply(HighCorr$Var1, function(x) which(colnames(Women) == x))

library(car)

library(dplyr)

# Select columns of interest

dfExpl <- Women[, c(170, 72:105, 107:111, 113:125)]

dfExpl$asrT[dfExpl$asrT==0] <- 0.001

dfExpl[is.na(dfExpl)] <- 0

# Set the random seed for reproducibility

set.seed(123)

# Create 10-fold cross-validation indices

folds <- createFolds(dfExpl$asrT, k = 10, list = TRUE, returnTrain = FALSE)

# Initialize vectors and data frames to store the results

rmseTrainExp <- rep(0, 10)

rmseTestExp <- rep(0, 10)

rsqTrainExp <- rep(0, 10)

rsqTestExp <- rep(0, 10)

vifList <- list()

coefList <- list()

tvalList <- list()

pvalList <- list()

# Loop over the folds

for (i in 1:10) {

# Get the indices for the current fold

testIndexExp <- folds[[i]]

trainIndexExp <- setdiff(1:nrow(dfExpl), testIndexExp)

# Split the data into train and test sets for the current fold

trainDataExp <- dfExpl[trainIndexExp, ]

testDataExp <- dfExpl[testIndexExp, ]

# Fit the model using the train dataset for the current fold

fitExp <- lm(asrT ~ ., data = trainDataExp)

# Store the VIF, coefficients, t-values, and p-values for each variable

vifList[[i]] <- data.frame(variables = names(vif(fitExp)),

vif = vif(fitExp))

coefList[[i]] <- coef(fitExp)

tvalList[[i]] <- summary(fitExp)$coefficients[, "t value"]

pvalList[[i]] <- summary(fitExp)$coefficients[, "Pr(>|t|)"]

# Predict the test data using the fitted model

predTestExp <- predict(fitExp, newdata = testDataExp)

# Calculate RMSE for train and test datasets for the current fold

rmseTrainExp[i] <- sqrt(mean((trainDataExp$asrT - predict(fitExp, trainDataExp))^2))

rmseTestExp[i] <- sqrt(mean((testDataExp$asrT - predTestExp)^2))

# Calculate r-squared for train and test datasets for the current fold

rsqTrainExp[i] <- summary(fitExp)$r.squared

rsqTestExp[i] <- cor(testDataExp$asrT, predTestExp)^2

}

# Calculate the average RMSE and r-squared over the 10 folds

avgRmseTrainExp <- mean(rmseTrainExp)

avgRmseTestExp <- mean(rmseTestExp)

avgRsqTrainExp <- mean(rsqTrainExp)

avgRsqTestExp <- mean(rsqTestExp)

cat("Average train RMSE: ", avgRmseTrainExp, "\n")

cat("Average test RMSE: ", avgRmseTestExp, "\n")

cat("Average train R-squared: ", avgRsqTrainExp, "\n")

cat("Average test R-squared: ", avgRsqTestExp, "\n")

# Calculate the mean VIF, coefficients, t-values, and p-values for each variable over the 10 folds

vifMean <- data.frame(variables = unique(vifList[[1]]$variables),

vif = unlist(lapply(unique(vifList[[1]]$variables),

function(v) mean(vifList[[1]][vifList[[1]]$variables == v, "vif"]))))

coefList<-as.data.frame(coefList)

tvalList<-as.data.frame(tvalList)

pvalList<-as.data.frame(pvalList)

coefList$mean <- rowMeans(coefList)

tvalList$mean <- rowMeans(tvalList)

pvalList$mean <- rowMeans(pvalList)

ModelExpSumm <- data.frame(Variable = vifMean$variables,

coefMean = coefList$mean[-1],

tvalMean = tvalList$mean[-1],

pvalMean = pvalList$mean[-1],

VIF = vifMean$vif)

# Identify variables with VIF < 7.5

VariablesforOLSlower7.5<-subset(ModelExpSumm,VIF<7.5)

VariablesforOLSlower7.5$index<-match(VariablesforOLSlower7.5$Variable,colnames(Women))

VariablesforOLS<-VariablesforOLSlower7.5$index

# OLS Regression --------------------------------------------------

dfOLS<-Women[,c(170,VariablesforOLS)]

dfOLS$asrT[dfOLS$asrT==0]<-0.001

dfOLS[is.na(dfOLS)]<-0

# Set the random seed for reproducibility

set.seed(123)

# Create 10-fold cross-validation indices

folds <- createFolds(dfOLS$asrT, k = 10, list = TRUE, returnTrain = FALSE)

# Initialize vectors and data frames to store the results

rmseTrainOLS <- rep(0, 10)

rmseTestOLS <- rep(0, 10)

rsqTrainOLS <- rep(0, 10)

rsqTestOLS <- rep(0, 10)

vifList <- list()

coefList <- list()

tvalList <- list()

pvalList <- list()

# Loop over the folds

for (i in 1:10) {

# Get the indices for the current fold

testIndexOLS <- folds[[i]]

trainIndexOLS <- setdiff(1:nrow(dfOLS), testIndexOLS)

# Split the data into train and test sets for the current fold

trainDataOLS <- dfOLS[trainIndexOLS, ]

testDataOLS <- dfOLS[testIndexOLS, ]

# Fit the model using the train dataset for the current fold

fitOLS <- lm(asrT ~ ., data = trainDataOLS)

# Store the VIF, coefficients, t-values, and p-values for each variable

vifList[[i]] <- data.frame(variables = names(vif(fitOLS)),

vif = vif(fitOLS))

coefList[[i]] <- coef(fitOLS)

tvalList[[i]] <- summary(fitOLS)$coefficients[, "t value"]

pvalList[[i]] <- summary(fitOLS)$coefficients[, "Pr(>|t|)"]

# Predict the test data using the fitted model

predTestOLS <- predict(fitOLS, newdata = testDataOLS)

# Calculate RMSE for train and test datasets for the current fold

rmseTrainOLS[i] <- sqrt(mean((trainDataOLS$asrT - predict(fitOLS, trainDataOLS))^2))

rmseTestOLS[i] <- sqrt(mean((testDataOLS$asrT - predTestOLS)^2))

# Calculate r-squared for train and test datasets for the current fold

rsqTrainOLS[i] <- summary(fitOLS)$r.squared

rsqTestOLS[i] <- cor(testDataOLS$asrT, predTestOLS)^2

}

# Calculate the average RMSE and r-squared over the 10 folds

avgRmseTrainOLS <- mean(rmseTrainOLS)

avgRmseTestOLS <- mean(rmseTestOLS)

avgRsqTrainOLS <- mean(rsqTrainOLS)

avgRsqTestOLS <- mean(rsqTestOLS)

# Print the results

cat("Average train RMSE: ", avgRmseTrainOLS, "\n")

cat("Average test RMSE: ", avgRmseTestOLS, "\n")

cat("Average train R-squared: ", avgRsqTrainOLS, "\n")

cat("Average test R-squared: ", avgRsqTestOLS, "\n")

# Calculate the mean VIF, coefficients, t-values, and p-values for each variable over the 10 folds

vifMean <- data.frame(variables = unique(vifList[[1]]$variables),

vif = unlist(lapply(unique(vifList[[1]]$variables),

function(v) mean(vifList[[1]][vifList[[1]]$variables == v, "vif"]))))

coefList<-as.data.frame(coefList)

tvalList<-as.data.frame(tvalList)

pvalList<-as.data.frame(pvalList)

coefList$mean <- rowMeans(coefList)

tvalList$mean <- rowMeans(tvalList)

pvalList$mean <- rowMeans(pvalList)

ModelOLSSumm <- data.frame(Variable = vifMean$variables,

coefMean = coefList$mean[-1],

tvalMean = tvalList$mean[-1],

pvalMean = pvalList$mean[-1],

VIF = vifMean$vif)

# Select variables based on criteria

selected_vars <- subset(ModelOLSSumm,((pvalMean < 0.05 | abs(tvalMean) > 2 ) & VIF<7.5))

# Selected Variables for Stepwise ----------------------------------------------

dfStepwise <- merge(selected_vars, VairablesSelectedRidge, by.x = "Variable", by.y = "value", all.x = TRUE, all.y=TRUE)

dfStepwise<-as.data.frame(dfStepwise[,1])

dfStepwise$index<-match(dfStepwise$`dfStepwise[, 1]` ,colnames(Women))

dfStepwise<-Women[,c(170,dfStepwise$index)]

colnames(dfStepwise)# Define your dependent variable

y <- dfStepwise$asrT

# Define your independent variables

x <- dfStepwise[, -c(1)] # Excluding the first column (assuming it contains row IDs)

# Create an initial model with all independent variables

model <- lm(y ~ ., data = x)

# Perform forward stepwise selection

step.model <- stepAIC(model, direction = "forward")

#Perform backward stepwise selection

step.model <- stepAIC(model, direction = "backward")

# Print the results

summary(step.model)

# Final variables for GWR Women -------------------------------------------

FinalVariablesWomen<-match(names(step.model$coefficients)[-1],colnames(Women))

FinalVariablesWomen<-Women[,c(1,2,170,FinalVariablesWomen)]

FinalVariablesWomenMean<- FinalVariablesWomen %>%

group_by(Province) %>%

summarise_all(mean)

write.xlsx(FinalVariablesWomenMean,"FinalVariablesWomenMean.xlsx")

FinalVariablesWomen2014<-subset(FinalVariablesWomen,Year=="2014",-c(2))

FinalVariablesWomen2015<-subset(FinalVariablesWomen,Year=="2015",-c(2))

FinalVariablesWomen2016<-subset(FinalVariablesWomen,Year=="2016",-c(2))

FinalVariablesWomen2017<-subset(FinalVariablesWomen,Year=="2017",-c(2))

FinalVariablesWomen2018<-subset(FinalVariablesWomen,Year=="2018",-c(2))

write.xlsx(FinalVariablesWomen2014,"FinalVariablesWomen2014.xlsx")

write.xlsx(FinalVariablesWomen2015,"FinalVariablesWomen2015.xlsx")

write.xlsx(FinalVariablesWomen2016,"FinalVariablesWomen2016.xlsx")

write.xlsx(FinalVariablesWomen2017,"FinalVariablesWomen2017.xlsx")

write.xlsx(FinalVariablesWomen2018,"FinalVariablesWomen2018.xlsx")

library(spacetime)

library(GWmodel)

# Load your data into R

data <- FinalVariablesWomen

# Convert to spatiotemporal data object

st_data <- STIDF(data[,c("y","x1","x2","x3")],

data = data.frame(time = data$Time),

sp = data.frame(x = data$Longitude, y = data$Latitude))

# Feature Selection Section For Men Population

# Lasso eldge reggresion --------------------------------------------------

ptot<-Men[,c(170,72,73,75:99,101:125)]

ptot$asrT[ptot$asrT==0]<-0.001

ptot[is.na(ptot)]<-0

# Train/Test split Ames Iowa housing dataset

set.seed(2345) # used to return consistent values for repeatability

ames_split <- initial_split(ptot, prop = .8, strata = "asrT")

ames_train <- training(ames_split)

ames_test <- testing(ames_split)

#Create dummy variables & taking the log

set.seed(2345)

ames_train_x <- model.matrix(asrT ~ ., ames_train)[, -1]

ames_train_y <- log(ames_train$asrT)

ames_test_x <- model.matrix(asrT ~ ., ames_test)[, -1]

ames_test_y <- log(ames_test$asrT)

#library(reshape2)

#library(plotly)

#Heatmap of variables to evaluate correlation

set.seed(2345)

ptot_cupdated <- ptot

cc2=cor(ptot_cupdated)

cc2_melt=melt(cc2)

gz=ggplot(cc2_melt,mapping=aes(x=Var1,y=Var2,fill=value))+

geom_tile()+

theme(axis.text.x = element_text(angle = 90, hjust = 1))+

theme(text = element_text(size=8))+

ggtitle("Heat Map for Housing Data: Numeric Predictors")+

ylab("")+

xlab("")+

scale_fill_distiller(palette = "Spectral")

ggplotly(gz, tooltip="text")

#library(caret)

#find variables with Correlation two by two by threshold above==0.9

findCorrelation(

cor(ptot_cupdated),

cutoff = 0.9,

verbose = TRUE,

names = TRUE,

exact = ncol(cor(ptot_cupdated)) < 100

)

#plot correlations plot

options(repr.plot.width=13, repr.plot.height=12) # adjust x,y size of plot

ggcorrplot(cc2, hc.order = TRUE, type = "lower",

lab = TRUE)+ggtitle('Correlation Plot of Ames Housing')

# Ridge Regression

options(repr.plot.width=10, repr.plot.height=8) # adjust x,y size of plot

ames_train_x <- scale(ames_train_x)

ames_test_x <- scale(ames_test_x)

ames_IA_ridge <- glmnet(

x = ames_train_x,

y = ames_train_y,

alpha = 0

)

plot(ames_IA_ridge, xvar = "lambda")

ridge_<- glmnet(x = ames_train_x, y = ames_train_y, alpha = 0)

sprintf('Max Lambda: %.3f',max(ridge_$lambda))

sprintf('Min Lambda: %.3f',min(ridge_$lambda))

#Find best values for lambda!

set.seed(2345)

ames_ridge_cv <- cv.glmnet(

x = ames_train_x,

y = ames_train_y,

alpha = 0

)

# plot results

plot(ames_ridge_cv)

#Training Our Model with CV using Ridge

mes_IA_ridge <- glmnet(x = ames_train_x,y = ames_train_y,alpha = 0)

# Tuning:

fit.ridge.cv <- cv.glmnet(ames_train_x, ames_train_y, type.measure="mse", alpha=0)

# Predicting Values:

pred_fit_ridge_train <- predict(fit.ridge.cv, ames_train_x, s = "lambda.min")

pred_fit_ridge_test <- predict(ames_IA_ridge, ames_test_x, s =fit.ridge.cv$lambda.min )

print('RIDGE:')

sprintf('Test RMSE: %.4f',sqrt(mean((pred_fit_ridge_test-ames_test_y)^2)))

sprintf('Train RMSE: %.4f',sqrt(mean((pred_fit_ridge_train-ames_train_y)^2)))

print('Your Vertical Lines')

r_squared_train <- cor(pred_fit_ridge_train, ames_train_y)^2

r_squared_test<- cor(pred_fit_ridge_test, ames_test_y)^2

sprintf('r_squared_train: %.4f',r_squared_train)

sprintf('r_squared_test: %.4f',r_squared_test)

min_mse_ridge_cv<-min(fit.ridge.cv$cvm) # minimum MSE

lambda_for_min_mse<-fit.ridge.cv$lambda.min # lambda for this min MSE

#1 std_err for min MSE

minMSE_1stderr_ridge<-fit.ridge.cv$cvm[fit.ridge.cv$lambda == fit.ridge.cv$lambda.1se]

lambda_for_minMSe_1stderr_ridge<-fit.ridge.cv$lambda.1se # lambda for this MSE

sprintf('Minimum MSE for CV: %.4f',min_mse_ridge_cv)

sprintf('Lambda for Corresponding Min. MSE : %.4f',lambda_for_min_mse)

sprintf('Min MSE for 1st Error: %.4f',minMSE_1stderr_ridge)

sprintf('Corresponding Lambda for 1 Std Err : %.4f',lambda_for_minMSe_1stderr_ridge)

sprintf('Log(Lambda Min. MSE): %.4f',log(fit.ridge.cv$lambda.min))

sprintf('Log(Lambda 1 Std Error MSE): %.4f',log(fit.ridge.cv$lambda.1se))

options(repr.plot.width=10, repr.plot.height=8) # adjust x,y size of plot

set.seed(2345)

ames_ridge_min <- glmnet(

x = ames_train_x,

y = ames_train_y,

alpha = 0

)

plot(ames_ridge_min, xvar = "lambda",main = 'Ridge: ')

abline(v = log(ames_ridge_cv$lambda.1se), col = "red", lty = "dashed")

abline(v = log(ames_ridge_cv$lambda.min), col = "red", lty = "dashed")

options(repr.plot.width=10, repr.plot.height=8) # adjust x,y size of plot

ames_ridge_cv<-coef(ames_ridge_cv, s = "lambda.min")

ames_ridge_cv<-as.data.frame(ames_ridge_cv[-1,])

ames_ridge_cv$row<-rownames(ames_ridge_cv)

names(ames_ridge_cv)<-c("row","value")

ames_ridge_cv$Coeff<-format(ames_ridge_cv$row,digit=4,scientific=FALSE)

# Ridge regression coefficients for scaled data

coef_ridge_scaled <- coef(fit.ridge.cv, s = lambda_for_min_mse)

# Unscale coefficients for original data

coef_ridge_unscaled <- coef_ridge_scaled[-1] / sd(ames_train_x)

coef_ridge_unscaled <- c(coef_ridge_scaled[1], coef_ridge_unscaled)

coef_ridge_unscaled<-as.data.frame(coef_ridge_unscaled[-1])

coef_ridge_unscaled$value<-rownames(ames_ridge_cv)

names(coef_ridge_unscaled)<-c("row","value")

coef_ridge_unscaled$Coeff<-format(coef_ridge_unscaled$row,digit=4,scientific=FALSE)

coef_ridge_unscaled$scaled_coeff<-ames_ridge_cv$Coeff

coef_ridge_unscaled$Coeffs<- abs(coef_ridge_unscaled$row)

VairablesSelectedRidge<-coef_ridge_unscaled[order(desc(coef_ridge_unscaled$Coeffs)),]

VairablesSelectedRidge<-data.frame(value=VairablesSelectedRidge[1:10,2])

ggplot() +

geom_point(coef_ridge_unscaled,mapping=aes(row, reorder(value,row), color = value > 0)) +

ggtitle("Influential variables") +

xlab("Coefficient") +

ylab(NULL)

# explanatory Regression --------------------------------------------------

HighCorr<-data.frame(Var1=findCorrelation(

cor(ptot_cupdated),

cutoff = 0.8,

verbose = TRUE,

names = TRUE,

exact = ncol(cor(ptot_cupdated)) < 100

))

HighCorr$Index <- lapply(HighCorr$Var1, function(x) which(colnames(Men) == x))

# Select columns of interest

dfExpl <- Men[, c(170,72,73,75:83,85,86,88:99,101:103,107:110,112,114:116,118,120:124)]

dfExpl$asrT[dfExpl$asrT==0] <- 0.001

dfExpl[is.na(dfExpl)] <- 0

# Set the random seed for reproducibility

set.seed(123)

dfExplScaled <- as.data.frame(scale(dfExpl[, -1]))

# Add the dependent variable to the scaled data frame

dfExplScaled$asrT <- dfExpl$asrT

# Set the random seed for reproducibility

set.seed(123)

# Create 10-fold cross-validation indices

folds <- createFolds(dfExplScaled$asrT, k = 10, list = TRUE, returnTrain = FALSE)

# Initialize vectors and data frames to store the results

rmseTrainExp <- rep(0, 10)

rmseTestExp <- rep(0, 10)

rsqTrainExp <- rep(0, 10)

rsqTestExp <- rep(0, 10)

vifList <- list()

coefList <- list()

tvalList <- list()

pvalList <- list()

# Loop over the folds

for (i in 1:10) {

# Get the indices for the current fold

testIndexExp <- folds[[i]]

trainIndexExp <- setdiff(1:nrow(dfExplScaled), testIndexExp)

# Split the data into train and test sets for the current fold

trainDataExp <- dfExplScaled[trainIndexExp, ]

testDataExp <- dfExplScaled[testIndexExp, ]

# Fit the model using the train dataset for the current fold

fitExp <- lm(asrT ~ ., data = trainDataExp)

# Store the VIF, coefficients, t-values, and p-values for each variable

vifList[[i]] <- data.frame(variables = names(vif(fitExp)),

vif = vif(fitExp))

coefList[[i]] <- coef(fitExp)

tvalList[[i]] <- summary(fitExp)$coefficients[, "t value"]

pvalList[[i]] <- summary(fitExp)$coefficients[, "Pr(>|t|)"]

# Predict the test data using the fitted model

predTestExp <- predict(fitExp, newdata = testDataExp)

# Calculate RMSE for train and test datasets for the current fold

rmseTrainExp[i] <- sqrt(mean((trainDataExp$asrT - predict(fitExp, trainDataExp))^2))

rmseTestExp[i] <- sqrt(mean((testDataExp$asrT - predTestExp)^2))

# Calculate r-squared for train and test datasets for the current fold

rsqTrainExp[i] <- summary(fitExp)$r.squared

rsqTestExp[i] <- cor(testDataExp$asrT, predTestExp)^2

}

# Calculate the average RMSE and r-squared over the 10 folds

avgRmseTrainExp <- mean(rmseTrainExp)

avgRmseTestExp <- mean(rmseTestExp)

avgRsqTrainExp <- mean(rsqTrainExp)

avgRsqTestExp <- mean(rsqTestExp)

cat("Average train RMSE: ", avgRmseTrainExp, "\n")

cat("Average test RMSE: ", avgRmseTestExp, "\n")

cat("Average train R-squared: ", avgRsqTrainExp, "\n")

cat("Average test R-squared: ", avgRsqTestExp, "\n")

# Calculate the mean VIF, coefficients, t-values, and p-values for each variable over the 10 folds

vifMean <- data.frame(variables = unique(vifList[[1]]$variables),

vif = unlist(lapply(unique(vifList[[1]]$variables),

function(v) mean(vifList[[1]][vifList[[1]]$variables == v, "vif"]))))

coefList<-as.data.frame(coefList)

tvalList<-as.data.frame(tvalList)

pvalList<-as.data.frame(pvalList)

coefList$mean <- rowMeans(coefList)

tvalList$mean <- rowMeans(tvalList)

pvalList$mean <- rowMeans(pvalList)

ModelExpSumm <- data.frame(Variable = vifMean$variables,

coefMean = coefList$mean[-1],

tvalMean = tvalList$mean[-1],

pvalMean = pvalList$mean[-1],

VIF = vifMean$vif)

# Identify variables with VIF < 7.5

VariablesforOLSlower7.5<-subset(ModelExpSumm,VIF<7.5)

VariablesforOLSlower7.5$index<-match(VariablesforOLSlower7.5$Variable,colnames(Men))

VariablesforOLS<-VariablesforOLSlower7.5$index

# OLS Regression --------------------------------------------------

dfOLS<-Men[,c(170,VariablesforOLS)]

dfOLS$asrT[dfOLS$asrT==0]<-0.001

dfOLS[is.na(dfOLS)]<-0

# Set the random seed for reproducibility

set.seed(123)

# Create 10-fold cross-validation indices

folds <- createFolds(dfOLS$asrT, k = 10, list = TRUE, returnTrain = FALSE)

# Initialize vectors and data frames to store the results

rmseTrainOLS <- rep(0, 10)

rmseTestOLS <- rep(0, 10)

rsqTrainOLS <- rep(0, 10)

rsqTestOLS <- rep(0, 10)

vifList <- list()

coefList <- list()

tvalList <- list()

pvalList <- list()

# Loop over the folds

for (i in 1:10) {

# Get the indices for the current fold

testIndexOLS <- folds[[i]]

trainIndexOLS <- setdiff(1:nrow(dfOLS), testIndexOLS)

# Split the data into train and test sets for the current fold

trainDataOLS <- dfOLS[trainIndexOLS, ]

testDataOLS <- dfOLS[testIndexOLS, ]

# Fit the model using the train dataset for the current fold

fitOLS <- lm(asrT ~ ., data = trainDataOLS)

# Store the VIF, coefficients, t-values, and p-values for each variable

vifList[[i]] <- data.frame(variables = names(vif(fitOLS)),

vif = vif(fitOLS))

coefList[[i]] <- coef(fitOLS)

tvalList[[i]] <- summary(fitOLS)$coefficients[, "t value"]

pvalList[[i]] <- summary(fitOLS)$coefficients[, "Pr(>|t|)"]

# Predict the test data using the fitted model

predTestOLS <- predict(fitOLS, newdata = testDataOLS)

# Calculate RMSE for train and test datasets for the current fold

rmseTrainOLS[i] <- sqrt(mean((trainDataOLS$asrT - predict(fitOLS, trainDataOLS))^2))

rmseTestOLS[i] <- sqrt(mean((testDataOLS$asrT - predTestOLS)^2))

# Calculate r-squared for train and test datasets for the current fold

rsqTrainOLS[i] <- summary(fitOLS)$r.squared

rsqTestOLS[i] <- cor(testDataOLS$asrT, predTestOLS)^2

}

# Calculate the average RMSE and r-squared over the 10 folds

avgRmseTrainOLS <- mean(rmseTrainOLS)

avgRmseTestOLS <- mean(rmseTestOLS)

avgRsqTrainOLS <- mean(rsqTrainOLS)

avgRsqTestOLS <- mean(rsqTestOLS)

# Print the results

cat("Average train RMSE: ", avgRmseTrainOLS, "\n")

cat("Average test RMSE: ", avgRmseTestOLS, "\n")

cat("Average train R-squared: ", avgRsqTrainOLS, "\n")

cat("Average test R-squared: ", avgRsqTestOLS, "\n")

# Calculate the mean VIF, coefficients, t-values, and p-values for each variable over the 10 folds

vifMean <- data.frame(variables = unique(vifList[[1]]$variables),

vif = unlist(lapply(unique(vifList[[1]]$variables),

function(v) mean(vifList[[1]][vifList[[1]]$variables == v, "vif"]))))

coefList<-as.data.frame(coefList)

tvalList<-as.data.frame(tvalList)

pvalList<-as.data.frame(pvalList)

coefList$mean <- rowMeans(coefList)

tvalList$mean <- rowMeans(tvalList)

pvalList$mean <- rowMeans(pvalList)

ModelOLSSumm <- data.frame(Variable = vifMean$variables,

coefMean = coefList$mean[-1],

tvalMean = tvalList$mean[-1],

pvalMean = pvalList$mean[-1],

VIF = vifMean$vif)

# Select variables based on criteria

selected_vars <- subset(ModelOLSSumm,((pvalMean < 0.05 | abs(tvalMean) > 2 ) & VIF<7.5))

# Selected Variables for Stepwise ----------------------------------------------

dfStepwise <- merge(selected_vars, VairablesSelectedRidge, by.x = "Variable", by.y = "value", all.x = TRUE, all.y=TRUE)

dfStepwise<-as.data.frame(dfStepwise[,1])

dfStepwise$index<-match(dfStepwise$`dfStepwise[, 1]` ,colnames(Men))

dfStepwise<-Men[,c(170,dfStepwise$index)]

colnames(dfStepwise)# Define your dependent variable

y <- dfStepwise$asrT

# Define your independent variables

x <- dfStepwise[, -c(1)] # Excluding the first column (assuming it contains row IDs)

# Create an initial model with all independent variables

model <- lm(y ~ ., data = x)

# Perform forward stepwise selection

step.model <- stepAIC(model, direction = "forward")

#Perform backward stepwise selection

step.model <- stepAIC(model, direction = "backward")

# Print the results

summary(step.model)

# Final variables for GWR Men -------------------------------------------

FinalVariablesMen<-match(names(step.model$coefficients)[-1],colnames(Men))

FinalVariablesMen<-Men[,c(1,2,170,FinalVariablesMen)]

FinalVariablesMen2014<-subset(FinalVariablesMen,Year=="2014",-c(2))

FinalVariablesMen2015<-subset(FinalVariablesMen,Year=="2015",-c(2))

FinalVariablesMen2016<-subset(FinalVariablesMen,Year=="2016",-c(2))

FinalVariablesMen2017<-subset(FinalVariablesMen,Year=="2017",-c(2))

FinalVariablesMen2018<-subset(FinalVariablesMen,Year=="2018",-c(2))

write.xlsx(FinalVariablesMen2014,"FinalVariablesMen2014.xlsx")

write.xlsx(FinalVariablesMen2015,"FinalVariablesMen2015.xlsx")

write.xlsx(FinalVariablesMen2016,"FinalVariablesMen2016.xlsx")

write.xlsx(FinalVariablesMen2017,"FinalVariablesMen2017.xlsx")

write.xlsx(FinalVariablesMen2018,"FinalVariablesMen2018.xlsx")
