# Supplementary material for: Exploring the geospatial epidemiology of breast cancer in Iran: identifying significant risk factors and spatial patterns for evidence-based prevention strategies
Source: BMC Cancer. 2023 Dec 11;23:1219. doi: 10.1186/s12885-023-11555-1 (PMC10712175; doi:10.1186/s12885-023-11555-1)
Supplement: Supplementary file 4 — Additional file 4. [file 12885_2023_11555_MOESM4_ESM.docx]

**Detailed results of the average of Breast cancer ASR in Geospatial Epidemiological Analysis of Breast Cancer in Iran**


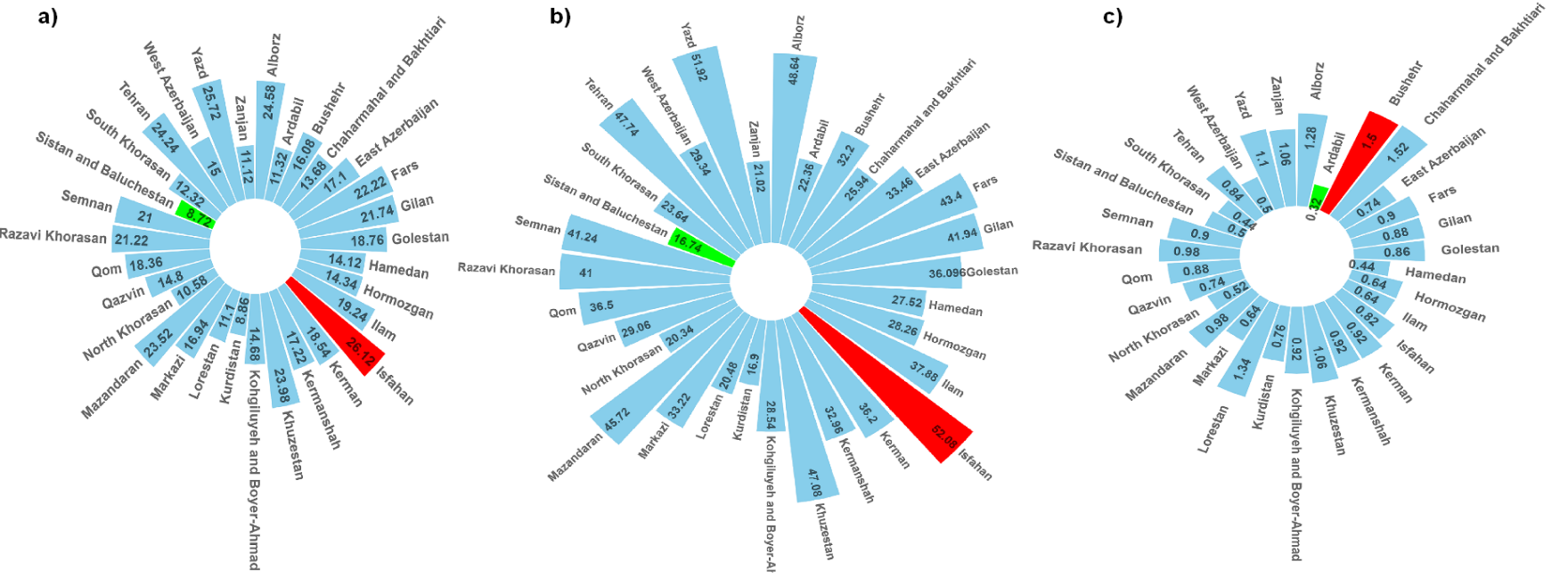
The average of Age-Standardized Incidence Rates (ASR) of Breast Cancer per 100,000 Population in Iran, Stratified by Sex and Province, for the Period 2014-2018.

**a)** The average of Breast Cancer ASR in gender-integrated population, **b)** The average of Breast Cancer ASR in Women, **c)** The average of Breast Cancer ASR in Men
