# Supplementary material for: Exploring the geospatial epidemiology of breast cancer in Iran: identifying significant risk factors and spatial patterns for evidence-based prevention strategies
Source: BMC Cancer. 2023 Dec 11;23:1219. doi: 10.1186/s12885-023-11555-1 (PMC10712175; doi:10.1186/s12885-023-11555-1)
Supplement: Supplementary file 5 — Additional file 5. [file 12885_2023_11555_MOESM5_ESM.docx]

**Spatial Autocorrelation Reports for the average of ASR in gender-integrated, Women and Men population**

**
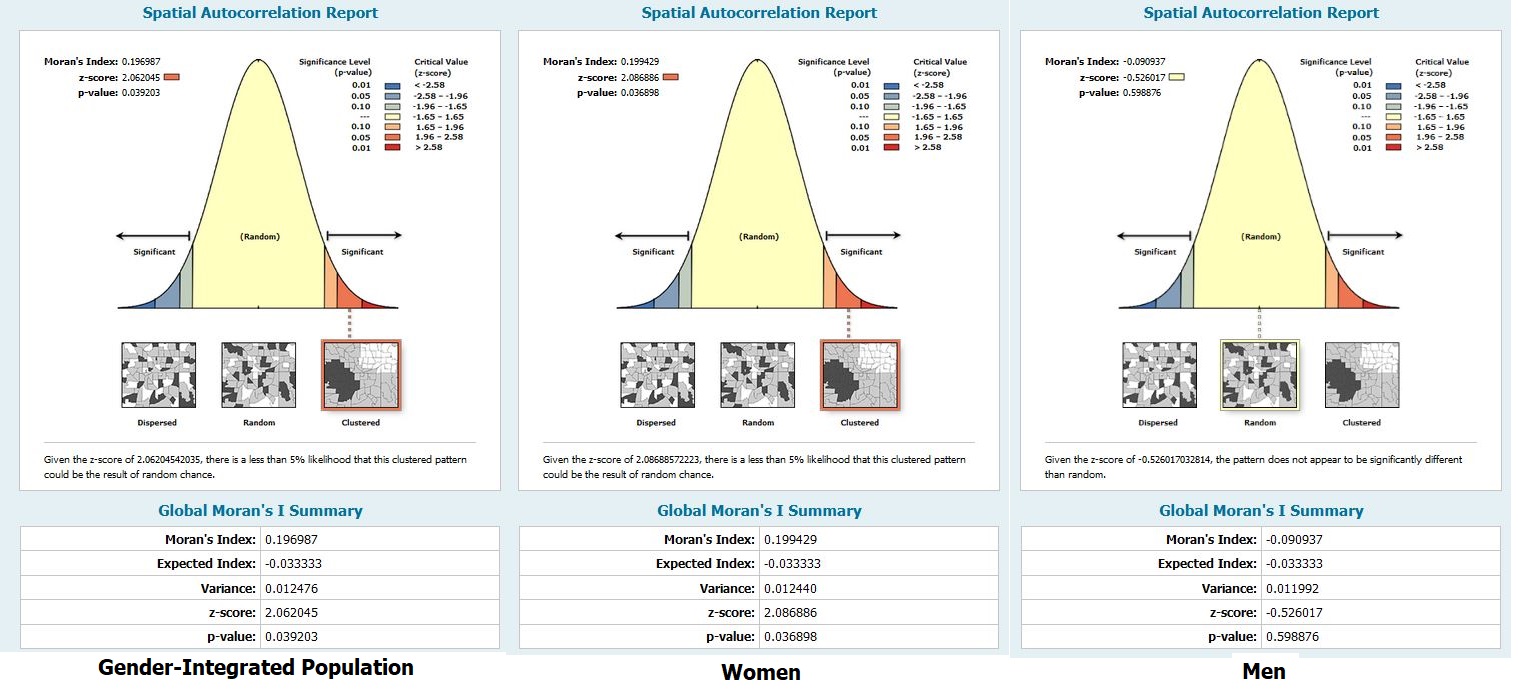
**
